# HLA-DR expression in cytotoxic T lymphocytes: a key to boost the therapeutic potential of T cell-based strategies for breast cancer

**DOI:** 10.3389/fimmu.2025.1653970

**Published:** 2025-09-19

**Authors:** Rute Salvador, Bruna Filipa Correia, Daniela Grosa, Telma Martins, Suelen Cristina Soares Baal, Diana Pereira Saraiva, Sofia Cristóvão-Ferreira, Isabel Lopes Pereira, Cátia Rebelo de Almeida, Rita Fior, Antonio Jacinto, Carolina Mathias, Sofia Braga, M. Guadalupe Cabral

**Affiliations:** ^1^ iNOVA4Health, NOVA Medical School, Faculdade de Ciências Médicas, NMS, FCM, Universidade NOVA de Lisboa, Lisboa, Portugal; ^2^ Hospital Professor Doutor Fernando Fonseca, EPE, Unidade Local de Saúde Amadora/Sintra, Amadora, Portugal; ^3^ Departamento de Genética, Universidade Federal do Paraná, Curitiba, PR, Brazil; ^4^ Instituto Português de Oncologia de Lisboa Francisco Gentil, Lisboa, Portugal; ^5^ Instituto CUF de Oncologia, Lisboa, Portugal; ^6^ Champalimaud Centre for the Unknown, Champalimaud Foundation, Lisboa, Portugal

**Keywords:** 3D co-cultures, immunomodulation, immunotherapy, cytotoxicity, adoptive T cell therapy, breast cancer, cytotoxic T lymphocytes

## Abstract

T cell-based therapies, involving *ex vivo* expansion of patients’ T lymphocytes, hold significant promise for chemotherapy resistant cases of breast cancer (BC), although their effectiveness remains challenging. Building on our previous findings that the expression of the antigen presenting molecule, HLA-DR, is crucial on tumor-infiltrating cytotoxic T lymphocytes (CTLs) for a favorable response to neoadjuvant chemotherapy, we further investigated the role of HLA-DR-expressing CTLs in anti-tumor responses and evaluated strategies to amplify these cells. Through *in vitro* and *in vivo* experiments, we demonstrated that HLA-DR expression on CTLs is important for effective tumor cell elimination. Notably, blocking HLA-DR or depleting CD4+ T cells impaired CTLs activation, suggesting a critical role for antigen presentation by CTLs to CD4+ T cells through HLA-DR in promoting robust anti-tumor responses. Based on these findings we optimized an *ex vivo* stimulation protocol that increases the proportion of HLA-DR+CTLs with improved cytotoxicity, prioritizing cell quality over yield. Moreover, we showed that adding anti-PD-1 to the stimulation, further upregulated HLA-DR expression, and intensified CTLs’ cytotoxic ability. This aligns with our *in silico* analysis suggesting a potential regulatory link between PD-1 and HLA-DR via non-coding RNAs. Overall, our findings open new avenues for advancing T cell-based therapies and improve the outcomes of chemotherapy-resistant-BC.

## 1 Introduction

BC is the most frequent type of cancer in women worldwide, accounting for up to 2 million new cases per year (1, 2).

One of the foremost challenges in addressing BC is its inherent heterogeneity, which influences the therapeutic options. Through specific biomarkers, including hormone receptor (HR) status and levels of the human epidermal growth factor receptor 2 (HER2), BC can be categorized into four subtypes: luminal A (HR+/HER2−), HER2+ (HR-/HER2+), luminal B (HR+/HER2+) and triple-negative (TNBC; HR−/HER2−). Each of these subtypes presents distinct risk factors for incidence, therapeutic response and disease progression (3, 4).

Despite the advancements in surgery, chemotherapy, radiation and more recently, immunotherapy, the limitations of BC treatment persist (5, 6).

Neoadjuvant chemotherapy (NACT) stands as a widely accepted approach for high-risk, locally advanced, or inoperable BC tumors (7), though less than 50% of patients achieve a pathological complete response (8, 9). Previously, we proposed CTLs expressing high levels of HLA-DR as a robust and independent biomarker for predicting the likelihood of BC patients successfully responding to NACT (10, 11). Nonetheless, the lack of effective options for BC patients with chemotherapy-resistant tumors poses a substantial clinical challenge, particularly for those ineligibles for prompt surgical intervention.

Recognizing the constraints posed by immune escape, immunotherapies offer a promising avenue for cancer treatment, although their effectiveness can vary due to differences in patients’ immune competency and heterogeneity of tumors (12). The long-standing view that breast tumors exhibit limited immunogenicity (13), has been challenged by the introduction of immune checkpoint inhibitors (ICIs). ICIs targeting programmed death-1 (PD-1) or its ligand (PD-L1) are now established as a standard first-line of therapy for advanced or metastatic PD-L1 positive TNBC (14). Furthermore, ICIs have also recently gained approval for high-risk early-stage TNBC (15). Nowadays, several clinical trials are trying to expand immunotherapy to all BC subtypes, with a particular focus on early-stage cases, where it may be more effective, primarily due to a less immunosuppressive tumor microenvironment (16).

CAR-T cell therapy is also being explored as an alternative immune-based treatment for BC. This innovative approach involves engineering patient’s T cells to express chimeric antigen receptors (CARs) that specifically target cancer cells. Recent studies have shown promising results in preclinical models, demonstrating the therapy’s ability to effectively recognize and eliminate BC cells. While CAR-T cell therapy has been primarily successful in treating hematologic malignancies, ongoing clinical trials are trying to optimize its use for solid tumors like BC. However, currently, there are no FDA-approved CAR-T cell therapies for solid tumors, including BC (17–19).

Adoptive T cell therapy (ATC) has also emerged as a significant therapeutic strategy in the fight against cancer. This approach is continuously evolving and under evaluation, both as a standalone treatment or in combination with other immunotherapies through ongoing clinical trials (20). Highlighting its potential in BC, a recent case demonstrated that the infusion of autologous T lymphocytes, which have been specifically designed to target neo-antigens and propagated *ex vivo*, was successfully employed in combination with ICIs, in a patient with chemo-refractory metastatic BC (21).

Despite the potential of ATC therapies in BC treatment, their integration into clinical practice poses significant challenges due to associated high costs, technical complexities and the toxicities related to excessive cytokine release (22). Furthermore, while ICIs have made significant advances in BC, they are predominantly focused on addressing TNBC, leaving limited options for other cancer subtypes (16). Nonetheless, the modulation of the immune system in BC patients appears to be an encouraging strategy for developing more personalized therapies.

Following the idea that HLA-DR-expressing CTLs are required in the tumor microenvironment to assist NACT in cancer elimination and exhibit anti-tumor properties, we previously suggested that the upsurge of CTLs with increased levels of HLA-DR expression might be considered in novel therapeutic approaches for BC (11). Thus, this paper further explores the therapeutic potential of HLA-DR-expressing CTLs and reports strategies to augment them. Specifically, our work underscores the relevance of HLA-DR expression levels in CTLs to monitor the quality of *ex vivo* manipulated cells, for the development of adoptive T cell transfer protocols. Furthermore, it demonstrates the synergistic effect of nivolumab, a monoclonal antibody targeting PD-1, and CTLs’ *ex vivo* short-term stimulation to elevate their HLA-DR expression and consequently enhance the cytotoxicity of these cells.

This synergy may be related to the fact that PD-1 in CTLs exhibits common correlated genes with HLA-DR, such as some known gene expression regulators, as revealed by our *in silico* analysis of BC scRNAseq databases.

This combined strategy offers a promising avenue for integration into future T lymphocyte-based approaches, to amplify immune responses and achieve more favorable outcomes for BC patients.

## 2 Methods

### 2.1 Human samples

This study was primarily performed using immune cells isolated from the buffy coats of 32 healthy donors, provided by Instituto Português do Sangue e da Transplantação (IPST). Additionally, 209 samples were used from a cohort of patients diagnosed with BC sourced from Hospital CUF Descobertas, Hospital CUF Tejo, Instituto Português de Oncologia de Lisboa Francisco Gentil, and Hospital Prof. Doutor Fernando Fonseca. Specifically, blood samples from 14 BC patients with advanced/metastatic disease and biopsies from 195 BC patients selected for NACT were utilized. Written informed consent from all individuals and approval from the Ethical Committees of the hospitals, IPST, and NOVA Medical School, were obtained. The study is in compliance with the Declaration of Helsinki.

Patients’ main characteristics are detailed in **Table 1**.

**Table 1:** Overview of participant characteristics enrolled in the study – biopsy and blood donors.

	Patients		Healthy donors
	Neoadjuvant breast cancer	Advanced breast cancer	
Number of subjects	195	14	32
Sample type	Biopsies	Blood	Blood
Age	Median: 57 (Range: 29 - 90)	Median: 58 (range: 47 - 84)	range: 18–65
Body Mass Index (BMI)	Median: 26.45 (range: 19.14 - 46.64)	Median: 26.06 (range: 18.37 - 35.56)	NA
Subtype: • ER+ (PR –/+) • HER2+ • TNBC	48.72% 37.95% 13.33%	71.42% 14.29% 14.29%	NA NA NA
Tumor dimension (mm)	Median: 32.91 (range: 6 - 200)	Median: 57.92 (range: 2.5 - 160)	NA
Ki67	Median: 35 (range: 1 - 97)	Median: 40 (range: 19 - 90)	NA
Axillary lymph node invasion status	Negative – 62.57% Positive – 37.43%	Negative – 50.00% Positive – 50.00%	NA
Grade	G1 - 20.29% G2 - 51.49% G3 - 28.22%	G1 – 0.00% G2 – 35.72% G3 - 28.57% Not known – 35.71%	NA
Treatment: • NACT • Other chemotherapeutic treatments	100% NA	35.71% 64.29%	NA

*NA, non-applicable.

Median values for age and body mass index (BMI) are included, along with key tumor characteristics such as dimension and Ki67 levels (related to tumor proliferation rate). Additionally, other clinical data including breast cancer subtype, node status, and grade are summarized. Healthy donors were recruited from the IPST and were recognized as healthy individuals with no known comorbidities.

Patients’ blood samples were collected using Vacutainer tubes with ethylenediaminetetraacetic acid (EDTA, Vacutest). Fresh biopsies were collected in Transfix (Cytomark) to preserve cellular antigens, allowing flow cytometry analysis.

Peripheral blood mononuclear cells (PBMCs) were isolated from whole blood or buffy coats using a Ficoll gradient (Sigma Aldrich) and cryopreserved in a solution containing 90% fetal bovine serum (FBS, Biowest) and 10% dimethyl sulfoxide (DMSO, Sigma Aldrich) for later use.

The biopsies underwent mechanical dissociation using a BD Medicon (BD Biosciences), filtered through a 30 µm nylon mesh (Sysmex), washed with 1X Phosphate-Buffered Saline (PBS), and then stained for flow cytometry.

### 2.2 Breast cancer cell lines

*In vitro* experiments were primarily carried out using the triple negative breast cancer (TNBC)-derived cell line MDA-MB-231. Additionally, some experiments were conducted with various BC cell lines, covering different BC subtypes, namely the BC cell lines BT-474 (HER-2+ subtype), HCC1806 (TNBC subtype), Hs578T (TNBC subtype) and MCF-7 (ER+ subtype). Hs578T, MCF-7, and MDA-MB-231 cell lines were cultured in Dulbecco’s Modified Eagle Medium (DMEM, Gibco) supplemented with 10% FBS (Biowest) and 1% Penicillin/Streptomycin (GE Healthcare). BT-474 and HCC1806 cell lines were cultured in RPMI 1640 (Gibco) supplemented with 10% FBS and 1% Penicillin/Streptomycin. Insulin (Sigma Aldrich) at 10µg/mL was additionally used in the culture of BT-474, Hs578T and MCF-7 cell lines. The cells were grown in monolayer cultures in T75 flasks under humidified conditions (37°C, 5% CO_2_) until they achieved 80-90% confluency. Subsequent cell passages were carried out by detaching the cells using TrypLE (Gibco).

Particularly, for *in vivo* experiments, Hs578T cancer cells were fluorescently labelled with TdTomato via lentiviral transduction. Cells were cultured in T175 flasks until 70-80% confluence and detached with EDTA 1 mM (diluted in DPBS 1X) and cell scrapers.

Cell lines were tested routinely for mycoplasma contamination.

### 2.3 Flow cytometry

For the flow cytometry analysis, biopsies, blood samples, or BC cells-PBMCs from co-cultures, were stained with a cocktail of monoclonal mouse anti-human conjugated antibodies (mAbs) after being processed to a single cell suspension. In the case of blood samples, the protocol included an additional step of red blood cell lysis using RBC lysis buffer (Biolegend) in the dark for 20 minutes at 4°C. Briefly, the staining protocol involved incubating cells with mAbs for 15 minutes at room temperature, followed by washing with 1 mL of PBS 1X and centrifugation at 300g for 5 minutes. Whenever intracellular markers were analyzed, cells were fixed and permeabilized with Fix/Perm kit (eBioscience) for 30 minutes at room temperature. Intracellular mAbs were added for 30 minutes, followed by a wash step with 1 mL of PBS 1X and centrifugation at 300g for 5 minutes.

The mAbs employed were: anti-CD45-PercP (clone HI30), anti-CD3-PercP (clone HIT3a), anti-CD3-APC (clone UCHT1), anti-CD25-PE (clone BC96), anti-CD4-FITC (clone OKT4), anti-CD8-PE (clone HIT8a), anti-CD8-PacificBlue (clone HIT8a), anti-HLA-DR-APC (clone L243), anti-Granzyme B-FITC (clone QA16A02), anti-CD69-PercP (clone FN50), anti-CD45-PercP (clone HI30), all from Biolegend. Cell viability was determined with BD Horizon™ Fixable Viability Stain 450 (BD Biosciences) incubated with the cell suspension for 20 minutes in the dark at 4°C.

Data acquisition was conducted using the BD Fluorescence Activated Cell Sorting (FACS) Canto II with FACSDiva Software v8 (BD Biosciences), and the results were analyzed using FlowJo software v10.

CTLs were identified as CD3+CD8+ events, based on sequential gating of single cells. CD4+ T cells, presented in the **Supplementary Material**, were defined as CD3+CD4+ cells. Representative gating strategies are shown in **Supplementary Figure S1**.

In general, data are presented as a percentage of the positive population with respect to the single cells’ gate or the positive population for a given marker within the CD8+ cells or the CD4+ cells in **Supplementary Material**.

### 2.4 Fluorescence activated cell sorting

To deplete CD4+ cells, PBMCs were stained with anti-CD45-PercP (clone HI30) and anti-CD4-FITC (clone OKT4). These cells were subsequently sorted into two distinct populations: CD45+/CD4+ and CD45+/CD4negative. For another experiment, PBMCs were sorted to isolate CD25+/HLA-DRnegative cells and CD25+/HLA-DR+ cells, after staining with anti-CD25-PE (clone BC96) and anti-HLA-DR-APC (clone L243). These sorted populations were then reintegrated into the remaining PBMCs. Sorting was performed using the FACS Aria III system from BD Biosciences with an efficiency above 90%.

### 2.5 Establishment of 3D co-cultures

A 3D co-culture involving BC cell lines and allogeneic PBMCs in a 1:3 ratio on agarose-coated plates was implemented, as previously established (23). Before co-culture, PBMCs underwent stimulation, for 48h, with mouse anti-human anti-CD3 (5 μg/mL), anti-CD28 (1 μg/mL), and rat anti-mouse IgG1 (5 μg/mL) as the crosslinking antibody (Biolegend). Additionally, interleukin 2 (IL-2) at 100 IU/mL and interleukin 12 (IL-12) at 20 ng/mL (PeproTech) were added to the PBMCs monoculture during the last 24h of the stimulation protocol.

After 24h of co-culturing PBMCs with the BC cell lines, in some experiments (drug screening), therapeutic antibodies were introduced in the 3D co-culture. The spheroids were removed from the plate after 48h, dissociated by pipetting and stained with the Fixable Viability Dye to evaluate, by flow cytometry, the percentage of viable cancer cells in the co-culture. A pan-leukocyte marker, anti-CD45 (clone HI30, Biolegend) was also used to distinguish between tumor and immune cells.

### 2.6 Expansion of HLA-DR+ cytotoxic T lymphocytes

PBMCs were initially seeded in 24 multi-well plates at a density of 1x10^6^ cells/mL in 1 mL of RPMI 1640 Medium supplemented with 10% FBS and 1% Penicillin/Streptomycin. For T lymphocytes’ activation and expansion, 5 mg/mL of mouse anti-human anti-CD3, 1µg/mL of mouse anti-human anti-CD28 and 5μg/mL of rat anti-mouse IgG1 (Biolegend) were added to the PBMCs culture at day 0. After 24h of incubation, various cocktails of interleukins were added to the culture. IL-2 was consistently utilized at a concentration of 100 IU/mL, while IL-7, IL-12, and IL-15 were used at concentrations of 20 ng/mL (PeproTech). The cells were incubated at 37 °C in a humidified atmosphere containing 5% CO_2_ for 14 days. To maintain optimal cell growth, every 2-3 days, half of the culture medium was replaced with fresh medium containing the interleukins. Throughout the incubation, the cells were monitored and counted at seven time points (i.e., days 0, 3, 5, 7, 9, 11, 14), using trypan blue exclusion dye (GE Healthcare). When the wells became confluent, the culture was divided into new wells. To assess the final cell numbers for each condition, the cell counts from all the wells in that condition were summed after counting.

For each condition, the frequency of HLA-DR+CTLs was monitored at the same time points, by flow cytometry.

### 2.7 HLA-DR blocking assay

Blockade of HLA-DR was performed using PBMCs obtained from healthy donors, cultured for 72h under different experimental conditions: without stimulation, with stimulation, and with stimulation along with an HLA-DR-blocking antibody. Stimulation was performed as described above, and for the HLA-DR blockade, 10µg/mL of purified anti-human HLA-DR (clone L243, Biolegend) was used. Following the 72h culture period, cells were harvested and labeled with anti-CD25 (clone BC96, Biolegend), anti-CD69 (clone FN50, Biolegend), and anti-Granzyme B (clone QA16A02, Biolegend), to assess the activation status of CTLs. Marker expression was analyzed specifically within the gated CD8+ T cell population, as defined in the gating strategy (**Supplementary Figure S2**). Similar analyses for CD4+ T cells were performed and are presented in the **Supplementary Material**.

### 2.8 Agents screening

Agents screening experiments were conducted using our established 3D co-culture platform (23). Briefly, the BC cell lines BT-474, HCC1806, Hs578T, MCF-7, and MDA-MB-231 were challenged with PBMCs treated as described above, plus the addition of the following candidates: Nivolumab (Anti-PD-1, Bristol-Myers Squibb), Bevacizumab (Anti-VEGF, Roche), provided by the Champalimaud Foundation, 4-1BB (anti-137, clone 4B4-1, Biolegend), and anti-OX40 (Anti-134, clone Ber-ACT35, Biolegend).

All the compounds were tested at a concentration of 50 µg/mL. The effect of the compounds on the cancer cells’ viability was assessed by flow cytometry, as referred above.

### 2.9 Breast cancer zebrafish xenograft experiments

*In vivo* experiments were performed at Champalimaud Foundation, using zebrafish model (*Danio rerio*) *Tg(fli1:GFP)*, which were maintained and handled in accordance with European Animal Welfare Legislation and Champalimaud Fish Platform Program. Embryos were maintained under standard conditions in E3 medium at 28.5°C and staged according to hours post-fertilization (hpf).

At 48 hpf, zebrafish larvae were anesthetized with Tricaine 1X (160 mg/L) and Hs578T TdTomato cells, alone or mixed with PBMCs at a ratio of 1:1, were microinjected into the perivitelline space (PVS) (24). For injection, PBMCs were previously resuspended in CellTracker^(TM)^ Deep Red (1:1000 from the stock, Thermo Scientific) and incubated for 37°C for 10min, followed by 15min at 4°C.

After injection, xenografts were left on embryonic medium (E3) at 34°C. 24h post injection, successfully injected xenografts were screened under fluorescence microscopy and stratified into classes based on tumor size. E3 media renewed daily, as well as the removal of dead xenografts. At the end of the assay, xenografts were sacrificed and fixed in 4% (v/v) formaldehyde (FA) (Thermo Scientific) overnight at 4°C.

In the day after fixation, FA was removed, and the xenografts were permeabilized with PBS-Triton 0.1%. Nuclei were counterstained with DAPI (0.05mg/mL) overnight at 4°C. In the next day, DAPI was removed by sequential washes with PBS-Triton 0.1% and xenografts were mounted between two coverslips with MOWIOL mounting medium. All images were obtained using a Zeiss LSM710 fluorescence confocal microscope. Tumor size quantification was performed as previously described (25).

### 2.10 Bioinformatic analysis

To investigate potential regulatory networks involving the *HLA-DR* and *PD1* genes, we used scRNA-seq data from BC patients, specifically focusing on the gene expression of CTLs. The data were extracted from the TIGER database (available at http://tiger.canceromics.org/#/), which serves as a repository for them. The analysis was performed using R version 4.4.0. The searching strategy for genes correlated with *HLA-DR* and *PD1* was divided into three stages: a) search for genes positively/negatively correlated with the *HLA-DR gene*; b) search for genes positively/negatively correlated with the *PD1 gene*; and finally, c) search for commonly observed genes. We adopted the correlation value cutoff |>0.5|, *p-value <*0.05 as a search criterion. To narrow down the search for shared targets between *HLA-DR* and *PD1*, only genes detected in the largest datasets were considered. Thus, for the genes correlated with *HLA-DR* and *PD1*, we used the genes that were significant in at least 3 datasets.

### 2.11 Statistical analysis

Statistical analysis was conducted using GraphPad Prism v8. Comparisons between samples were conducted using appropriate statistical tests, including the nonparametric Mann-Whitney test, paired t-test, one-way ANOVA, or two-way ANOVA with multiple comparisons, depending on the experimental design and data distribution. All experiments were carried out with at least three independent biological replicates. For experiments involving PBMCs, each biological replicate was obtained from a different donor to account for inter-donor variability. Statistical significance was determined with p-values less than 0.05 considered significant. The levels of significance were denoted as follows: one symbol (*) for p<0.05, two symbols (**) for p<0.01, three symbols (***) for p<0.001, and four symbols (****) for p<0.0001.

## 3 Results

### 3.1 HLA-DR expression on CTLs allows breast cancer cells’ elimination

Previously, we reported that HLA-DR levels in CTLs (analyzed within CD8+ population) assessed in biopsies is a robust biomarker for predicting BC response to NACT (10, 11). Additionally, we demonstrated a positive correlation between HLA-DR levels in tumor-infiltrating CTLs and HLA-DR levels in systemic CTLs (10).

**Figure 1:**
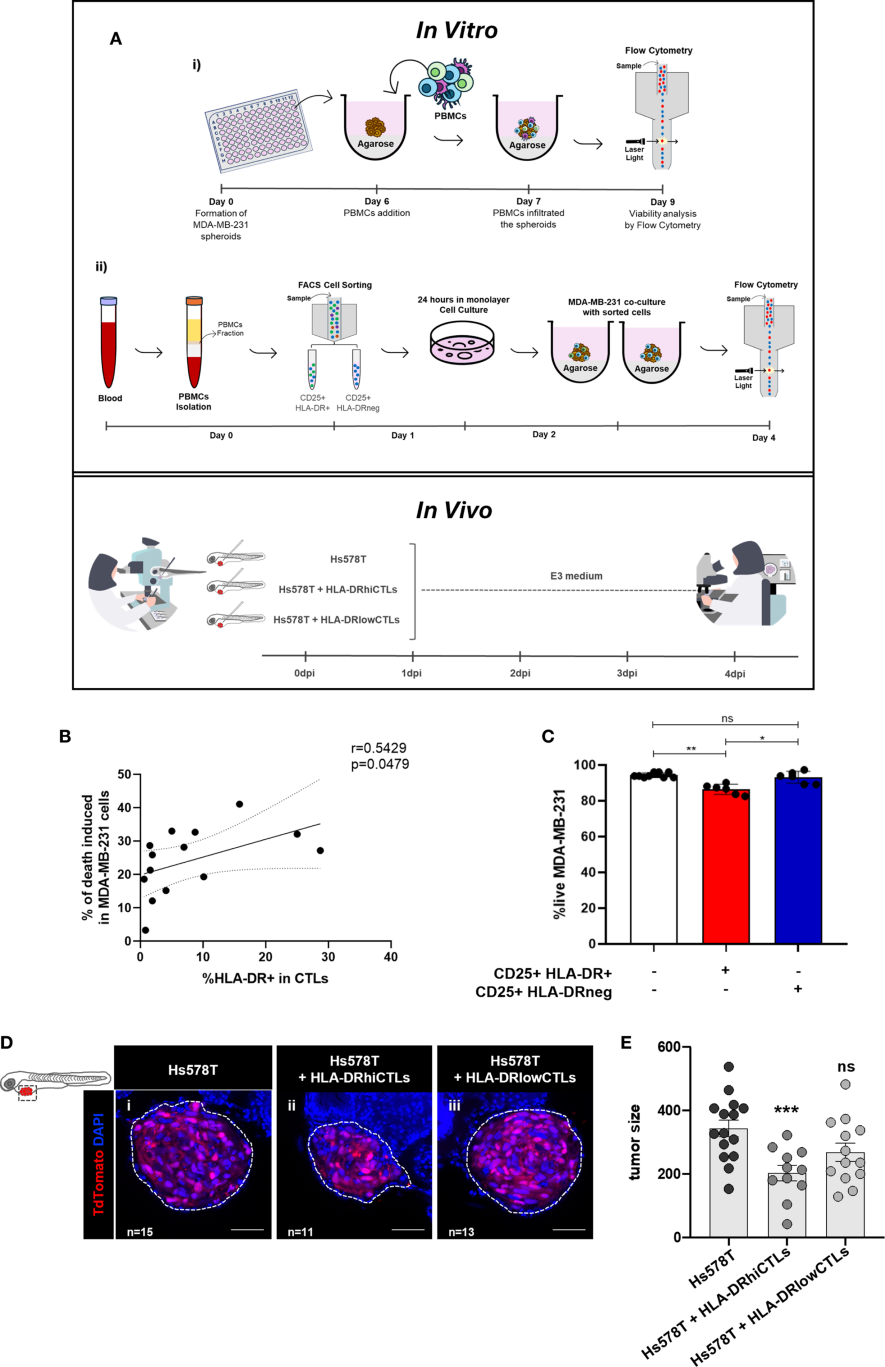
BC cells elimination requires HLA-DR expression on CTLs. **(A)** Schematic representation of the methodology employed. In the *in vitro* experiments: (i) the viability of BC cell lines was analyzed via flow cytometry after the addition of PBMCs derived from BC patients, which were previously stimulated. (ii) Fluorescence-activated cell sorting (FACS) was performed to separate two populations of activated immune cells (CD25+), one expressing HLA-DR (HLA-DR+) and the other lacking this molecule (HLA-DRneg). These sorted populations were then reintegrated into the remaining PBMCs. The cytotoxic capacity of both populations was assessed via flow cytometry after co-culturing with spheroids of BC cells (23). In the *in vivo* experiments, xenografts were established in Tg(*fli1:GFP*) zebrafish embryos, which express GFP, allowing tumor visualization by confocal microscopy. At 48 h post-fertilization, zebrafish were injected into the perivitelline space with Hs578T BC cells, either alone or mixed with PBMCs. Immune cells were obtained from donors with high frequency of HLA-DR in CTLs (HLA-DRhiCTLs) or with CTLs expressing low HLA-DR levels (HLA-DRlowCTLs). Four days post-injection, the cytotoxic capacity of the immune cells was evaluated by analyzing tumor size in the zebrafish via confocal microscopy. **(B)** The relationship between the percentage of CTLs (CD8+ T cells’ population) expressing HLA-DR in BC patients’ blood and the efficacy of their PBMCs in eliminating tumor cells was analyzed (Spearman r=0.5429, p=0.0479, n=14). **(C)** The viability of the MDA-MB-231 BC cell line (white bars, n=9) was examined following the addition of sorted CD25+HLA-DR+CTLs to the spheroids (red bar, p=0.0031, n=6), or the addition of CD25+HLA-DRnegCTLs (blue bar, ns, n=6). **(D)** Representative confocal microscopy images from the zebrafish xenografted model illustrate the impact of PBMCs enriched in HLA-DRhiCTLs compared to HLA-DRlowCTLs on tumor size. Scale bars represent 50 µm. **(E)** Quantification of tumor size was performed based on confocal microscopy analysis of zebrafish injected with PBMCs from donors enriched in either HLA-DRhiCTLs (p<0.001, n=11) or HLA-DRlowCTLs (ns, n=13). For tumor size evaluation, the ImageJ Cell Counter plugin was applied: the total number of DAPI+ cells was calculated as AVG (3 slices: Zfirst, Zmiddle, Zlast) × total number of slices/1.5. Data are represented as mean ± SD, *p < 0.05, **p < 0.01, ***p < 0.001, ns, non-statistical.

In this paper, to further investigate the anti-tumor effect of HLA-DR-expressing CTLs from the blood of BC patients and compare the tumor cell-elimination capacity of a PBMC population enriched in HLA-DR-expressing CTLs with that of a less enriched population, we conducted *ex vivo* and *in vivo* experiments (**Figure 1A**). Specifically, we employed an *in vitro* system that leverages the allogeneicity between a BC cell line and patient-derived PBMCs, enabling the assessment of T cell cytotoxicity (22). This setup allowed us to observed that when CD3/CD28-stimulated PBMCs were added to spheroids of the BC cell line MDA-MB-231 (23), led to a decrease in tumor cell viability, an effect dependent on the frequency of HLA-DR-expressing CTLs within the PBMCs (**Figure 1B**). Indeed, a positive correlation was established between the percentage of HLA-DR-expressing CTLs in the blood of BC patients and the ability of PBMCs to kill tumor cells (Spearman r=0.5429, p=0.0479, **Figure 1B**). Moreover, the addition of sorted HLA-DR-expressing CTLs to the BC spheroid showed an enhanced cytotoxic capacity in comparison to the addition of sorted CTLs without HLA-DR (p=0.0323, **Figure 1C**). Notably, both sorted populations exhibited equivalent levels of the classical activation marker CD25, highlighting the pivotal role of HLA-DR in augmenting CTLs-mediated tumor cell elimination.

These observations were further corroborated *in vivo*, through experiments with a zebrafish avatar model that yielded comparable results to the spheroids. A key advantage of this model is that adaptive immunity fully matures only at 2–3 weeks post-fertilization, providing a unique window to study human tumor-immune interactions in a controlled environment. This competence, provided a complementary and accurate assessment of the role of HLA-DR-expressing CTLs in tumor reduction *in vivo* (26–28). Specifically, xenografted zebrafish bearing the human BC cell line Hs578T, injected with PBMCs from BC patients with a high frequency of CTLs expressing high levels of HLA-DR showed a significant reduction in tumor size (p<0.001) compared to those injected with PBMCs from donors with low frequency of these cells (**Figures 1D, E**).

These results showed that HLA-DR-expressing CTLs are not only a biomarker for NACT response, as we previously reported (10, 11) but also possess more competence in promoting tumor cell elimination both *in vitro* and *in vivo*.

### 3.2 HLA-DR in CTLs and CD4+ T cells are essential for effective CTLs’ activation and mount an anti-tumor immune response

To explore the therapeutic potential of HLA-DR in CTLs, we investigated the activation and cytotoxic profile of CD3/CD28-stimulated CTLs when HLA-DR was blocked by a specific antibody. For this, we assessed the expression of diverse markers that reflect different stages of CTLs activation and Granzyme B, an effector molecule associated with cytotoxic function.

**Figure 2:**
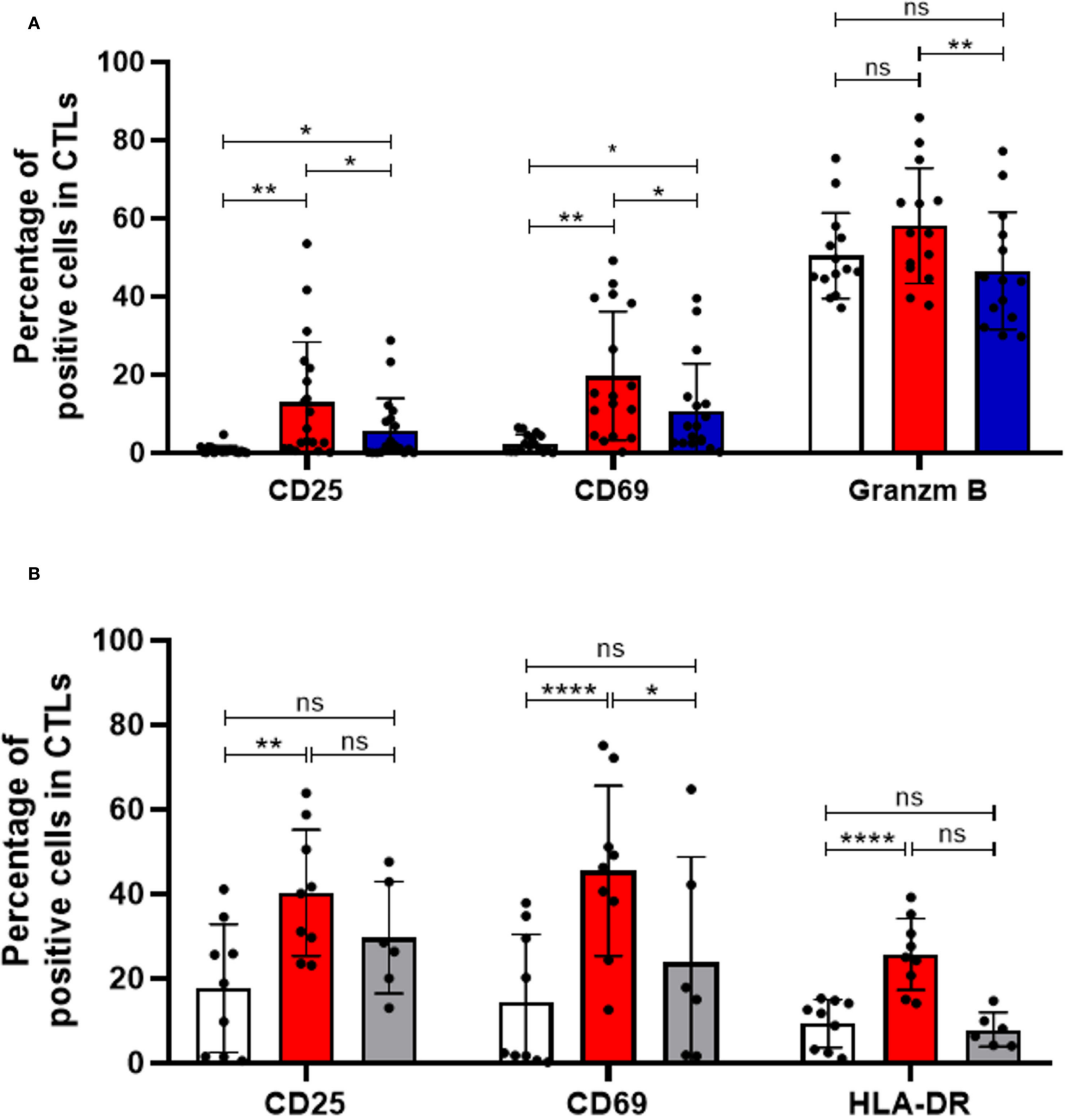
HLA-DR-expressing CTLs and CD4+ T cells are essential for effective CTLs activation and immune response. **(A)** CTLs activation following PBMCs stimulation was assessed by flow cytometry, measuring the frequency of CD69+ (n=17, p=0.0114), CD25+ (n=19, p=0.0262), and Granzyme B+ cells (n=14, p=0.0029) within the CTLs population. PBMCs were either left unstimulated (white bars) or stimulated via CD3 and CD28 co-stimulation (red bars), in the presence or absence of an anti-HLA-DR blocking antibody (blue bars). **(B)** Additional experiments assessed CTLs activation in the presence (red bars, n=9) or absence (grey bars, n=6) of CD4+ T cells, compared with unstimulated controls (white bars, n=9). CTLs activation was evaluated based on the expression of activation markers under these different conditions. Data are represented as mean ± SD, *p < 0.05, **p < 0.01, ***p < 0.001, ****p < 0.0001, ns, non-statistical.

Notably, blocking HLA-DR led to a reduction in the capacity of CTLs to become activated and cytotoxic, upon stimulation, as evidenced by a decreased frequency of CD25+, CD69+ and Granzyme B+ cells within the CTLs population (**Figure 2A**). In contrast, HLA-DR blockade had no significant impact on the activation of CD4+ T cells, which were analyzed separately (**Supplementary Figure S3**). As expected, the blockade of HLA-DR significantly decreased the frequency of HLA-DR+CTLs, similarly to those observed in non-stimulated conditions (**Supplementary Figure S4**). Even if the total PBMCs were used in our experiments, and the blockade may affect HLA-DR expressed by other cells besides CTLs, these findings underscore the critical importance of HLA-DR expression within CTLs themselves, in facilitating their activation and cytotoxic ability.

Importantly, to ensure the specificity of our results, we included isotype controls in parallel experiments. The addition of an isotype control antibody, at the same concentration as the HLA-DR blocking antibody, did not impact T cell activation or cytotoxicity, confirmed that the observed effects were specific to HLA-DR blockade (**Supplementary Figure S5**).

Additionally, considering that HLA-DR is the antigen-presenting molecule that enables antigen presentation to CD4+ T cells, we conducted experiments with PBMCs depleted of CD4+ T cells. Interestingly, stimulation of PBMCs depleted of CD4+ T cells resulted in minimal effect on CTL activation (**Figure 2B**), as evidenced by the largely unchanged percentage of cells positive for various activation markers (grey bars), contrary to the conditions where CD4+ T cells were present (red bars, p < 0.05, **Figure 2B**). Interestingly, among the markers tested, only CD69 - an earlier marker of T cell activation - showed a modest increase upon stimulation in the absence of CD4^+^ T cells, possibly indicating a transient, initial activation signal (**Figure 2B**). These observations underscore the importance of CD4+ T cells for robust and prolonged CTL activation.

Disrupting the interaction between CTLs and CD4+ T cells through HLA-DR blockade or CD4+ T cells depletion may compromise anti-tumor immune responses, suggesting a critical role for both HLA-DR and CD4+ T cells in CTLs activation and cytotoxic capabilities.

### 3.3 Therapeutic potential of CTLs could be boosted through short-term expansion

Recent efforts have focused on refining the *ex vivo* expansion of T cells for therapeutic purposes. This pursuit aims to bolster their quantity for adoptive T cell transfer protocols and enhance their ability to recognize and destroy malignant cells, thereby improving treatment efficacy (29, 30).

**Figure 3:**
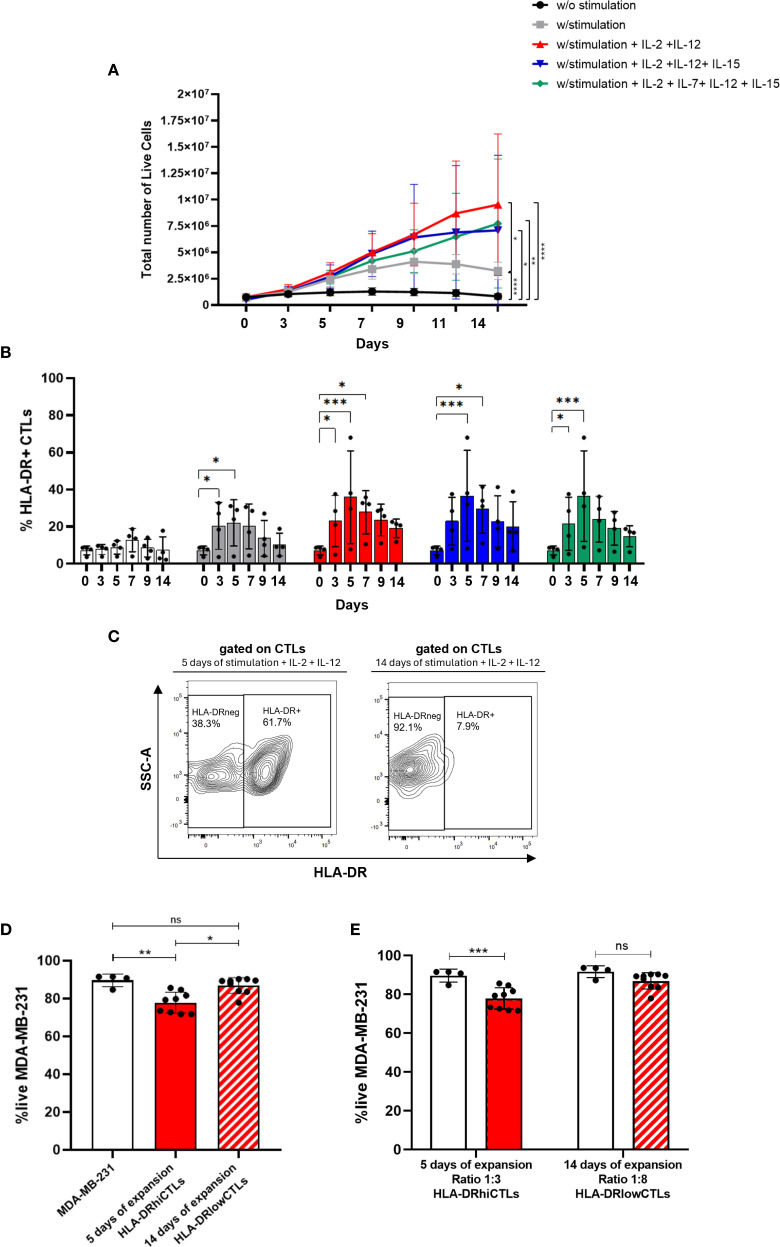
Therapeutic potential of CTLs could be boosted through short-term expansion. **(A)** PBMCs were cultured *ex vivo* for up to 14 days under different stimulation protocols. Conditions included: unstimulated cells (black line, n=6); stimulation with anti-CD3 (5 μg/mL) and anti-CD28 (1 μg/mL) (grey line, n=6); stimulation combined with cytokines IL-2, IL-7, IL-12, and IL-15 (green line, n=4); stimulation with IL-2, IL-12, and IL-15 (blue line, n=4); and stimulation with IL-2 and IL-12 (red line, n=6). **(B)** The percentage of HLA-DR-expressing CTLs was evaluated across these same stimulation conditions over a 14-day period (n=4). White bars: unstimulated condition; Grey bars: anti-CD3 and anti-CD28 PBMCs stimulation; Green bars: stimulation in combination with IL-2, IL-7, IL-12, and IL-15; Blue bars: stimulation plus IL-2, IL-12, and IL-15; Red bars: stimulation plus IL-2 and IL-12. **(C)** Contour plot illustrating HLA-DR expression on CTLs following stimulation with IL-2 and IL-12 for 5 days (left side) and 14 days (right side). **(D)** *In vitro* 3D co-cultures were performed using MDA-MB-231 BC spheroids cultured alone (white bar, n=4), with PBMCs stimulated for 5 days (red bar, n=9), or for 14 days (red striped bar, n=9). The experimental setup aimed to compare cytotoxic potential, and statistical analysis yielded p = 0.0036. **(E)** *In vitro* 3D co-cultures of BC cell line MDA-MB-231 in a spheroid structure alone (white bars, n=4), with PBMCs short-term stimulated (5 days), added to the culture at a ratio of 3 PBMCs:1BC cell (red bars, n=9) and with PBMCs long-period stimulated (14 days), added to the culture at a ratio of 8 PBMCs:1BC cell (red striped bar, n=9). Experiments were performed using PBMCs isolated from healthy donors. Data are represented as mean ± SD, *p < 0.05, **p < 0.01, ***p < 0.001, ****p < 0.0001, ns, non-statistical.

In this context, we explored the dynamics of T cell expansion with a specific focus on HLA-DR-expressing CTLs, using PBMCs isolated from healthy donors. We evaluated various expansion protocols for immune cells, aiming to optimize their quantity and functionality. Our findings revealed that immune cells can be successfully expanded *ex vivo* for up to 14 days using T cell receptor stimulation combined with cytokine cocktails (**Figure 3A**). The most effective expansion protocol utilized anti-CD3 (5 μg/mL) and anti-CD28 (1 μg/mL) antibodies, along with IL-2 and IL-12 (**Figure 3A**). Therefore, we opted for this method in the subsequent experiments.

However, while total numbers of PBMCs increased during the expansion protocol, we observed that specifically HLA-DR-expressing CTLs increased during the first days but suffered a decline after 4 to 5 days, regardless of the cytokine cocktail employed (**Figure 3B**). Furthermore, the expression level of HLA-DR in CTLs, follows the same tendency (**Supplementary Figure S6**), with a peak of expression around 4–5 days. Interestingly, our results also demonstrated that a short-term stimulation protocol (5 days) led to an upregulation of HLA-DR expression on CTLs compared to prolonged expansion method (14 days) (**Figure 3C**). Additionally, experiments employing 3D co-culture models showed increased viability of tumor cells when co-cultured with lymphocytes expanded for 14 days, indicating reduced cytotoxic activity of these cells compared to short-term stimulated cells (5 days; p=0.0036) (**Figure 3D**). Notably, we also demonstrated that cells expanded for a longer period exhibited diminished cytotoxic capacity even when added to BC cells in a higher ratio (8 PBMCs to 1 BC cell), compared to cells expanded for a shorter duration, which were added to BC cells only at a ratio of 3 PBMCs to 1 BC cell. Although PBMCs expanded for 14 days, even when used at a higher effector-to-target (E:T) ratio (8000 cells), did not induce a significant reduction in MDA-MB-231 cell viability (**Figure 3E**), in contrast to short-term expanded PBMCs (3000 cells), which were more effective despite being present at a lower E:T ratio. This higher cytotoxic capacity exhibited by the less expanded PBMCs is most likely because short stimulation led to more HLA-DR-expressing CTLs than long stimulation, as shown in **Figure 3C**.

These results emphasize the importance of prioritizing expansion protocols that guarantee the effective cytotoxic capabilities of CTLs, which we demonstrated to be strongly dependent on HLA-DR expression, achieved through short-term simulation.

### 3.4 Anti-PD-1 treatment contributes to increment HLA-DR in CTLs and amplifies their anti-tumor activity

Using the 3D co-culture model we established (23) as a screening platform, we tested potential agents aimed at augmenting CTLs’ HLA-DR expression and consequently their cytotoxicity against BC cells, in line with the results previously shown. Our screening encompassed several promising agents and different BC cell lines (**Supplementary Table S1**).

Following our previous characterization of the immune profile of HLA-DR+CTLs compared to HLA-DRneg CTLs, which revealed that several stimulatory co-receptors are more highly expressed in HLA-DR+CTLs (**Supplementary Figure S7**), we hypothesized that targeting these co-receptors with agonists could promote the conversion of HLA-DRneg CTLs into an HLA-DR+CTLs phenotype. To explore this possibility, we included in our screening, two of these agonists, the anti-CD137 and the anti-CD134/OX40, both of which are currently being investigated in several clinical trials.

Additionally, we included Nivolumab, an immune checkpoint inhibitor that targets PD-1, boosting the immune response against cancer cells, via PD-1/PD-L1 axis blockade, and Bevacizumab, a monoclonal antibody that targets and neutralizes vascular endothelial growth factor (VEGF), which has also been reported as having immunostimulatory properties (31, 32). Both have been used extensively in clinical practice for different solid cancers.

**Figure 4:**
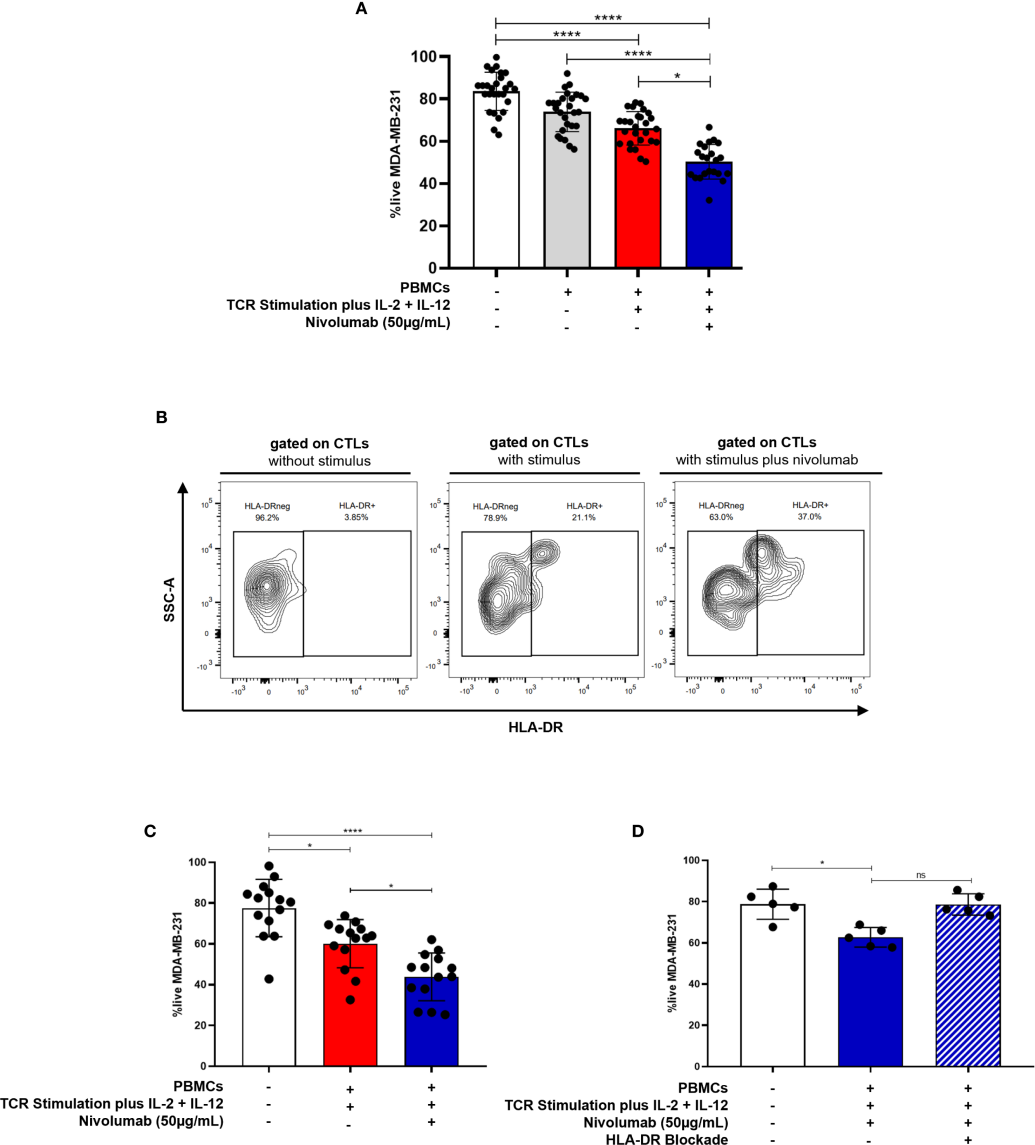
Anti-PD-1 treatment potentiates the anti-tumor capacity of CTLs against BC. **(A)** The viability of the MDA-MB-231 BC cell line in the 3D co-culture alone (white bar, n=27), incubated in the presence of unstimulated PBMCs (grey bar, n=27), incubated in the presence of stimulated PBMCs (red bar, n=27) and incubated in the presence of stimulated PBMCs plus the addition of the anti-PD-1, Nivolumab (blue bar, n=22). **(B)** Representative contour plot showing the frequency of HLA-DR+CTLs within PBMCs from healthy donors following stimulation with or without Nivolumab. **(C)** The viability of the MDA-MB-231 BC cell line alone (white bar, n=14), incubated in the presence of stimulated PBMCs which have been isolated from metastatic BC patients (red bar, n=14) and incubated in the presence of those stimulated PBMCs plus Nivolumab (blue bar, n=14, p=0.0487). **(D)** The viability of the MDA-MB-231 BC cell line alone (white bar, n=5), incubated in the presence of stimulated PBMCs from healthy donors plus Nivolumab (blue bar, n=5, p=0.0159), and incubated in the presence of stimulated PBMCs plus Nivolumab and an anti-HLA-DR blocking antibody (blue striped bar, n=5, non-statistical). Data are represented as mean ± SD, *p < 0.05, ****p < 0.0001, ns, non-statistical.

Nivolumab significantly potentiated the anti-tumor properties of activated CTLs against MDA-MB-231 and HCC1806, while the other tested drugs did not show any effect on improving cytotoxicity against these BC cell lines (p=0.0163, **Figure 4A**, **Supplementary Figure S8**, **Supplementary Table S1**). These experiments were performed using PBMCs obtained from healthy donors and stimulated as previously referred.

Notably, our results also indicated that Nivolumab treatment *in vitro* increases the percentage of HLA-DR+CTLs within PBMCs from healthy donors (**Figure 4B**).

Importantly, consistent with the enhanced cytotoxicity observed in **Figure 4A**, PBMCs derived from patients with metastatic BC, who naturally exhibit low or null basal levels of HLA-DR expression in CTLs also showed increased cytotoxic capacity following stimulation plus *ex vivo* Nivolumab treatment (*p* = 0.0487, **Figure 4C**).

Supporting our finding that Nivolumab boosts CTL cytotoxicity, through HLA-DR increment, blockade of HLA-DR in 3D co-cultures, also performed using PBMCs from healthy donors, dampened the effect of Nivolumab, resulting in a diminished cytotoxic capacity of CTLs (**Figure 4D**). Notably, unstimulated PBMCs exposed to Nivolumab did not significantly impact tumor cells viability (**Supplementary Figure S9**). This suggests that Nivolumab acts more as an adjuvant, improving T cell-mediated tumor cell killing only in the presence of additional microenvironmental stimuli.

In line with this idea, only a subset of BC patients undergoing anti-PD-1 therapy exhibited an increased frequency of CTLs expressing HLA-DR in their blood (**Supplementary Figure S10A**) along with elevated HLA-DR expression levels on CTLs (**Supplementary Figure S10B**), four months after treatment initiation. This observed heterogeneity may reflect differences in tumor microenvironment factors, which can variably support or impair effective CTLs activation and effector function.

Overall, our findings suggest that anti-PD-1 treatment holds considerable potential in boosting CTLs’ cytotoxicity through HLA-DR, beyond mere stimulation, especially against BC subtypes with elevated PD-L1 expression (**Figure 4A**, **Supplementary Figure S8**, **Supplementary Table S1**).

Although immunotherapy with anti-PD-1 in BC has primarily been used for TNBC cases, considered the most immunogenic subtype, our analysis of immune features in patients’ biopsies from TNBC, ER+ and HER2+ BC subtypes, revealed comparable frequencies of infiltrating CTLs and similar PD-L1 expression across all subtypes (**Supplementary Figure S11**), at least at the biopsy level. These findings suggest that the proposed approach could potentially be applied to all BC subtypes. Interestingly, since HER2+ tumors exhibited lower frequencies of HLA-DR-expressing infiltrating CTLs, this subtype might derive greater benefit from interventions like the ones here proposed.

### 3.5 Several ncRNAs co-correlated with the expression of HLA-DR and PD1 genes in CTLs

To investigate potential regulatory networks involving the *HLA-DR* and *PD1* genes, we used scRNA-seq data from BC patients extracted from the TIGER database. We focused on gene expression data from CTLs. Detailed gene lists for each dataset can be found in the **Supplementary Material**. We did not find significant correlation values regarding negatively correlated genes for both *HLA-DR* and *PD1*, therefore, our analysis only considered positively correlated genes with both genes.

**Figure 5:**
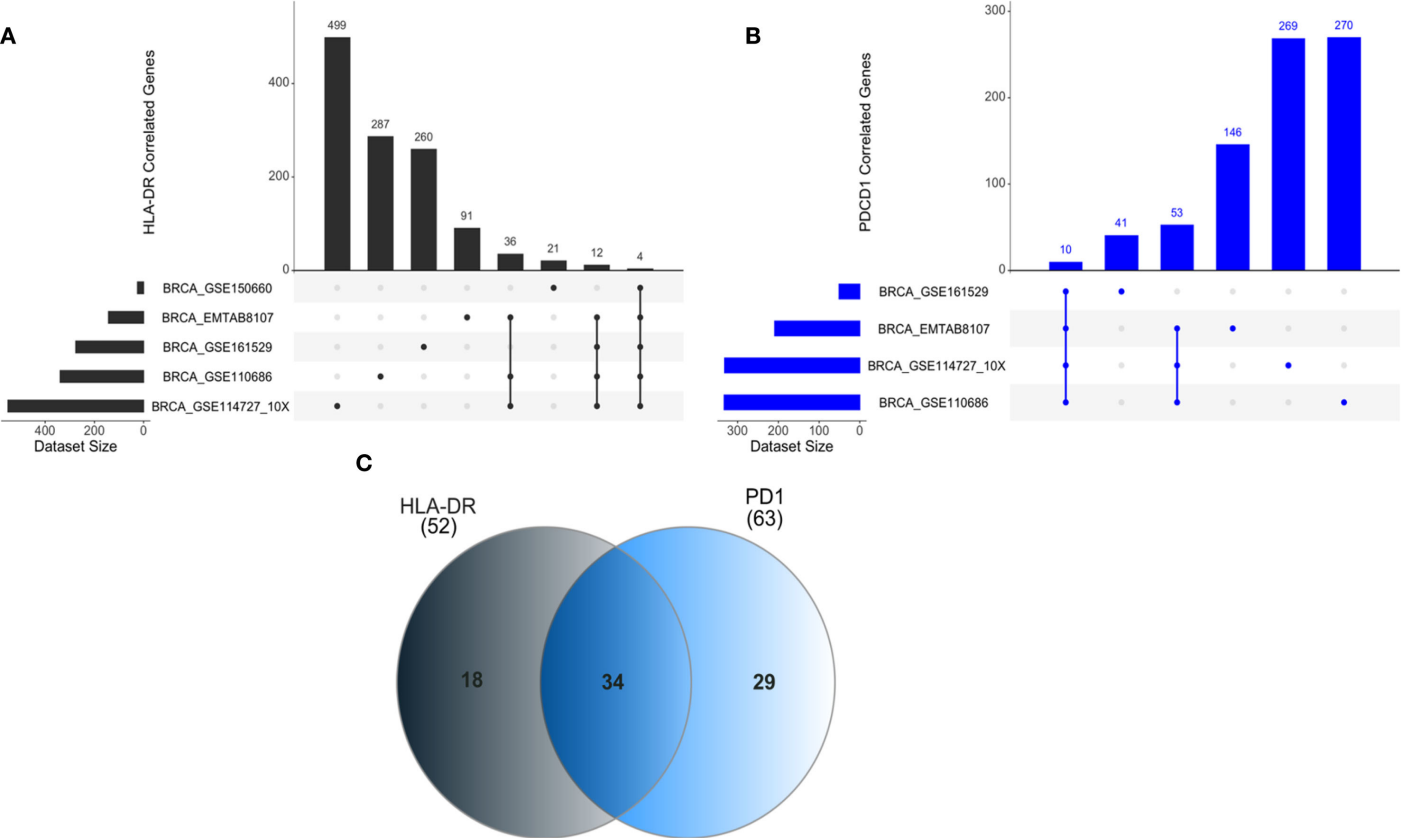
ncRNAs positively correlated with *HLA-DR* and *PD1* gene expression in CTLs. **(A)** Correlation analyses for HLA-DRA were performed using multiple breast cancer single-cell RNA-seq datasets, including BRCA_GSE114727_10x (n=499 genes correlated) and BRCA_GSE150660 (n=21 genes correlated), among others. Common genes shared across all datasets were identified. **(B)** For PD1 (PDCD1), datasets such as BRCA_GSE110686 (n=270 genes correlated) and BRCA_GSE114727_10x (n=269 genes correlated) were analyzed, with overlapping genes highlighted across datasets. **(C)** A Venn diagram summarizes exclusive and shared genes positively correlated with HLA-DR and PD1 expression, including 34 genes common to both.

For the *HLA-DR* gene, we found five scRNA-seq datasets from CTLs, named BRCA_GSE150660, BRCA_GSE110686, BRCA_GSE114727_10X, BRCA_GSE161529 and BRCA_EMTAB8107 (**Figure 5A**). Considering these datasets, we identified genes significantly correlated with *HLA-DR* (r > 0.5; p < 0.05) across the largest number of datasets, highlighting those with potential biological significance within the regulatory network of interest. The same analysis logic was applied to the *PD1 (PDCD1)* gene, based on four scRNA-seq datasets, namely: BRCA_GSE110686, BRCA_GSE114727_10X, BRCA_GSE161529 and BRCA_EMTAB8107 (**Figure 5B**). The information about genes and their correlation value is present in supplementary **Supplementary Tables S2**–**S10**.

Our analysis revealed 34 genes positively correlated with both *HLA-DR* and *PD1* (**Figure 5C**).

Notably, several non-coding RNAs (ncRNAs) were among these correlated genes. Specifically, four of the five datasets that include 52 genes for *HLA-DR*, found a positive correlation with ncRNAs, specifically with three long non-coding RNA (lncRNAs). For *PD1*, considering the 63 genes shared at least in three datasets, we also detected a positive correlation with lncRNAs. In **Table 2** we present all lncRNAs that have a positive correlation with *HLA-DR* or *PD1*. In bold, we highlight two lncRNAs, MIR155HG and KCNQ1OT1, that were consistently shared across different datasets and may play a role in the regulatory axis of HLA-DR expression in response to anti-PD1 treatment.

**Table 2:** lncRNAs positively correlate with HLA-DR and PD1 in each dataset.

Dataset	Gene 1	Gene 2	Correlation value
BRCA_GSE110686	**HLA-DR**	**MIR155HG**	0.79
		**KCNQ1OT1**	0.81
		LINC01480	0.54
	**PD1**	**MIR155HG**	0.79
		**KCNQ1OT1**	0.79
		AC133644.2	0.79
		LINC01480	0.60
BRCA_GSE114727_10X	**HLA-DR**	**MIR155HG**	0.68
		**KCNQ1OT1**	0.63
	**PD1**	**MIR155HG**	0.85
		**KCNQ1OT1**	0.78
		AC133644.2	0.74
		AC017002.1	0.67
		LINC01480	0.61
BRCA_GSE161529	**HLA-DR**	**MIR155HG**	0.52
		LINC01857	0.55
	**PD1**	LINC01943	0.56
BRCA_EMTAB8107	**HLA-DR**	**KCNQ1OT1**	0.67
		**MIR155HG**	0.59
	**PD1**	**MIR155HG**	0.76
		**KCNQ1OT1**	0.74
		LINC01480	0.70
		AC133644.2	0.66

The lncRNAs MIR155HG and KCNQ1OT1 are highlighted in bold since they are genes shared between HLA-DR and PD1.

## 4 Discussion

Projections indicate a substantial increase in BC incidence and mortality rates in the coming years, underscoring the urgent need for collective efforts to mitigate this growing burden (33).

Immunotherapy emerged as a promising approach to treat BC. However, challenges such as tumor heterogeneity and immune evasion persist, hindering the advancement of this therapeutic avenue (34–36). The success of the KEYNOTE-355 trial (37) and the subsequent regulatory approval of pembrolizumab for TNBC have spurred additional clinical trials exploring alternative immunotherapy modalities, such as adoptive T cell therapies, across all BC subtypes (38, 39).

Despite recent advancements, a significant portion of BC patients, especially those with advanced diseases, fail to respond to conventional and immunotherapy treatments, often due to the suboptimal effectiveness of CTLs.

Previously, we demonstrated that the presence of HLA-DR-expressing CTLs in the tumor microenvironment is crucial for a favorable response to NACT in BC patients (10, 11), likely because this molecule enhances the anti-tumor activity of CTLs. Notably, our findings are consistent with other studies indicating the importance of HLA-DR expression for effective response to NACT in BC patients, associated with increased IL-12 and IFN-γ plasma levels (10, 11, 40).

In the current study, by employing 3D allogeneic co-cultures and zebrafish xenograft models, we established that PBMCs enriched with HLA-DR-expressing CTLs exhibited superior cytotoxic against BC cells compared to PBMCs with few or no HLA-DR-expressing CTLs. These models have been widely recognized as valuable tools for uncovering fundamental aspects of T cell biology or as platforms for drug screening. Specifically, the co-culture model takes advantage of alloreactivity to investigate the cytotoxic ability of T cells under certain stimuli (41–45), while the zebrafish avatar model serves as a complement in cancer research due to their transparency, which allows real-time monitoring of tumor progression and cancer-immune interactions in a physiologically relevant context (27, 28).

Importantly, in the *in vivo* zebrafish system, immune cell populations enriched for HLA-DRhiCTLs demonstrated an increased ability to reduce tumor size, thereby corroborating our *in vitro* findings. This consistency across models supports the relevance of HLA-DR expression in enhancing CTLs function and validates the utility of these complementary experimental systems in dissecting immune responses to cancer. Moreover, it highlights the potential translational significance of enhancing HLA-DR+CTLs in future therapeutic strategies.

Our experiments also revealed that HLA-DR blockade reduces CTLs’ activation and cytotoxicity. However, we recognize that other immune cell populations, including antigen-presenting cells (APCs) and CD4+ T cells, may also be affected by the blockade, potentially contributing to the observed impairment of CTLs activation. Dissecting the specific contributions of individual immune subsets under HLA-DR blockade remains an important but challenging task, requiring carefully designed experimental approaches.

Additionally, we observed that depletion of CD4^+^ T cells reduced CTL activation and cytotoxicity, highlighting a functional interplay between these subsets. HLA-DR is a classical MHC class II molecule typically expressed on professional APCs such as dendritic cells, macrophages, and B cells. Remarkably, however, activated CTLs can upregulate HLA-DR on their surface, an atypical feature that enables them to present exogenous antigens in a manner similar to APCs. Indeed, HLA-DR^+^ CTLs possess the machinery for antigen processing and peptide loading onto HLA-DR molecules (46). When these cells display peptide fragments, they may directly stimulate CD4^+^ helper T cells through TCR recognition of the HLA-DR–peptide complex. This establishes a phenomenon known as T–T antigen presentation (47), in which one T cell functions as an antigen-presenting cell for another.

During such interactions, CTLs could provide antigen to CD4^+^ T cells, which in turn deliver essential help signals—including interleukin-2, interferon-γ, and CD40L—that reinforce CTL proliferation, survival, and differentiation into effector and long-lived memory cells (48–51). These reciprocal interactions may generate a positive feedback loop that enhances both the strength and durability of CTL responses.

**Figure 6:**
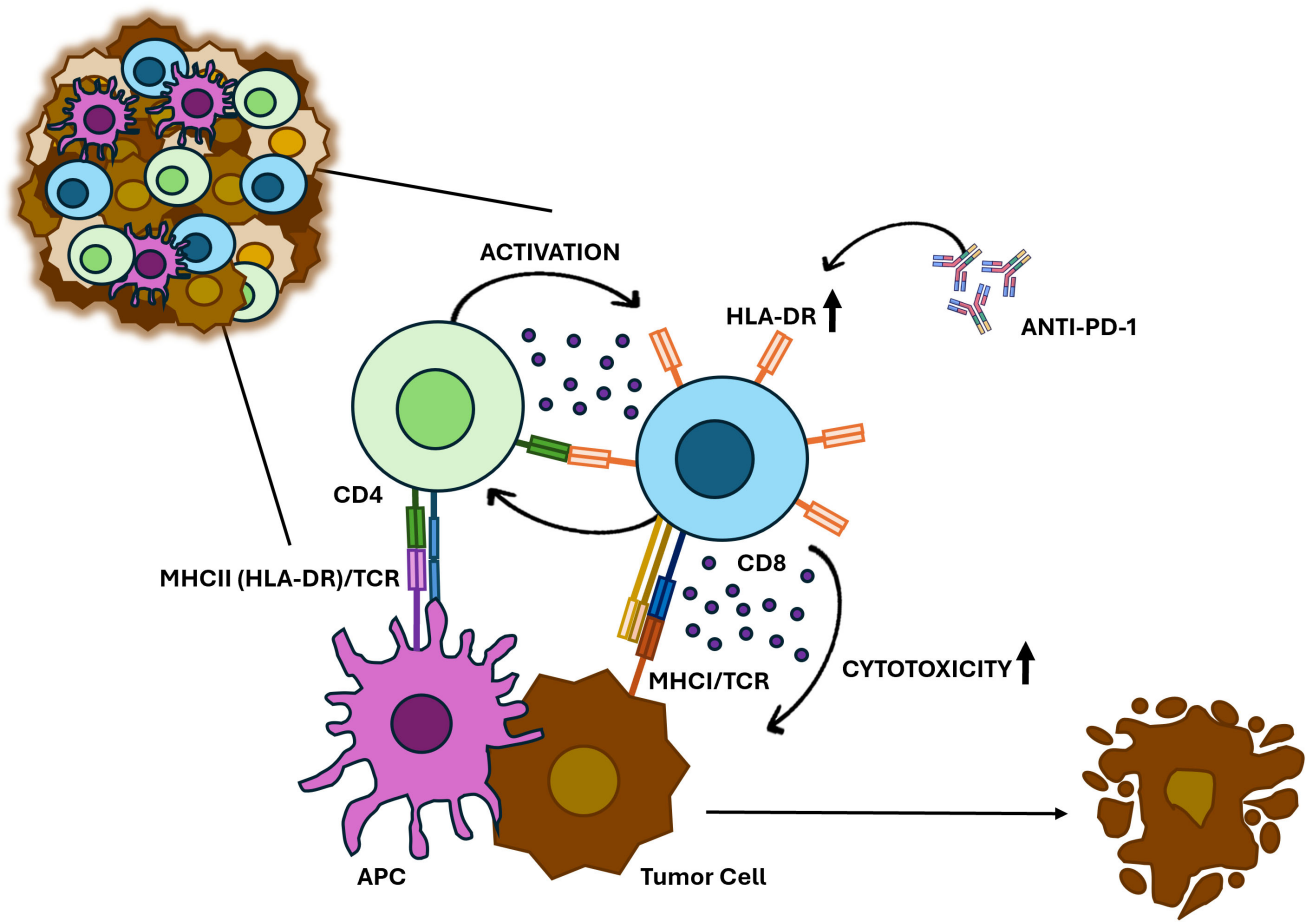
Proposed model for HLA-DR-expressing CTLs in anti-cancer immune response. HLA-DR-mediated antigen presentation primes CTLs to recognize and eliminate BC cells. HLA-DR-expressing CTLs may also interact with CD4+ T cells, which release cytokines such as IFN-γ, reinforcing CTL activation and sustaining a robust immune response against tumors. Additionally, anti-PD-1 treatment increases the HLA-DR expression in CTLs and significantly boosts their cytotoxicity.

We emphasize, however, that our current experiments do not provide direct evidence that CTLs present antigen to CD4+ T cells or that CD4+ T cells directly influence CTL activity. Alternative mechanisms—such as indirect effects mediated by dendritic cells—cannot be excluded. Indeed, recent work by Lei and colleagues (52) showed that activated CD4+ T cells can produce IFN-β, which enhances antigen presentation by certain dendritic cells and thereby improves CTL priming and antitumor activity. Such findings highlight that CD4^+^ T cell help may reinforce CTL responses both directly, through potential T–T antigen presentation, and indirectly, through modulation of professional APCs (52). Thus, while we hypothesize a T–T antigen presentation model (**Figure 6**), supported by prior reports of T cell–T cell synapses (47, 53), this suggestion requires further experimental validation.

Importantly, similar forms of immune regulation have been described in chronic viral infections (e.g., HIV, CMV, EBV) and autoimmunity, where they may amplify immune responses when professional APCs are limited (54). Collectively, our results suggest that HLA-DR^+^ CTLs may serve as noncanonical antigen-presenting cells, with CD4^+^ T cell help - either direct or indirect - acting as a crucial amplifier of their cytotoxic function against BC cells. Nevertheless, future studies employing cell-type–specific HLA-DR blockade, conditional knockout models, and live-cell imaging of CTL–CD4^+^ T cell synapses will be required to dissect this mechanism in greater detail and to clarify its relevance within the specific immunological context reported here.

Recent studies have been exploring strategies to enhance tumor immunity against cancer cells. For instance, it was demonstrated in pre-clinical models that targeting IL-2 to CD8+ T cells promotes robust effector T cell responses and potent anti-tumor immunity (55). Given our results, in this work we also investigated ways to expand the population of HLA-DR-expressing CTLs.

The expression of HLA-DR in CTLs has been associated with prolonged antigen exposure, increased cytotoxic potential, and immune regulation, making it a valuable biomarker for disease progression and therapeutic targeting. Consistent with previous studies (40), including our own (10, 11), which have shown that HLA-DR+CTLs exhibit elevated levels of key effector molecules (e.g., IFN-γ, TNF-α, granzyme B, and perforin), our findings further underscore that HLA-DR is not merely a marker of T cell activation but also a reflection of sustained immune engagement and functionality. This suggests an enhanced cytotoxic role for HLA-DR+CTLs, potentially driven by a feedback loop mechanism.

Because HLA-DR expression peaks early during culture (around 4–5 days) and declines thereafter, even as total PBMC numbers continue to increase, we then compared the cytotoxicity of CTLs under short- versus long-term expansion conditions. This comparison allowed us to assess whether the frequency of HLA-DR+ CTLs correlates with functional anti-tumor activity. Our results indicate that short-term expansion, together with IL-2 and IL-12 supplementation, enriches for HLA-DR+ CTLs, yielding PBMCs with superior cytotoxicity against BC cells, even at lower effector-to-target ratios. In contrast, prolonged PBMCs stimulation increases the total number of cells but results in diminished HLA-DR-expressing CTLs and do not affect tumor cells viability. Although we did not perform phenotypic analysis of exhaustion or dysfunction and thus cannot exclude additional factors influencing long-term expanded cells, these findings suggest that short-term expansion produces lymphocytes with higher functional potential for potential T cell–based therapies.

Notably, the addition of Nivolumab, an anti-PD-1 antibody, synergistically increased the percentage of HLA-DR expression in CTLs and boosted the ability of PBMCs population to eliminate tumor cells. This suggests that treating PBMCs with anti-PD-1 could enhance CTL-mediated anti-tumor responses, not only because this antibody blocks inhibitory signals from PD-1/PD-L1 interactions, but also because it upregulates HLA-DR expression, probably promoting a positive feedback loop of cytokine release and sustained activation (**Figure 6**).

Both HLA-DR and PD-1 have been extensively studied in tumor biology contexts (40, 46, 47, 55); however, little is known about HLA-DR role in tumor-infiltrating CTLs, or regarding the link between both molecules. For instance, a study by Heng Yu et al. (56) on laryngeal squamous cell carcinoma found that high expression of tumor HLA-DR is associated with improved response to anti-PD-1 therapy (56), but this research focused on HLA-DR expressed by tumor cells and not specifically on CTLs.

Although it is known that upon anti-PD-1 treatment, PD-1 expression typically increases in lymphocytes as part of the normal activation process (57, 58), the interplay between this molecule and HLA-DR, which also rises in lymphocytes under stimulatory conditions, remains unclear.

Therefore, aiming at investigating shared regulatory pathways between *HLA-DR* and *PD1* genes, we conducted a data analysis using scRNA-seq data. Thirty-four genes were identified that showed a positive correlation with both genes. A noteworthy finding was the presence of lncRNAs in this list. lncRNAs are transcripts longer than 500 nucleotides (59), which are now recognized for their potential to encode small functional peptides (60, 61). Specifically, the lncRNAs MIR155HG and KCNQ1OT1 showed positive correlations with both *HLA-DR* and *PD1*, exhibiting relatively high correlation values. Although these data do not establish causality, they may indicate a potential association between these lncRNAs and the regulatory axis involving HLA-DR and PD-1 expression. Validation experiments are beyond the scope of the current study, but these findings highlight an interesting avenue for future research.

Overall, our study underscores the crucial role of HLA-DR expression in CTLs for the elimination of BC cells. We hypothesize that manipulating HLA-DR expression, through combined approaches, could significantly augment CTLs-mediated tumor killing, particularly for BC patients with lower HLA-DR expression in CTLs, potentially applied across all the BC subtypes.

Despite the promising results, it is also important to acknowledge the limitations of this study, particularly the use of 3D co-culture experiments employing allogeneic models, which might yield different results to autologous systems where both BC and immune cells are derived from the same patient. Indeed, allogeneic T cells are not primed for specific recognition of tumor antigens presented by the recipient’s MHC, leading to limited modeling of antigen-specific responses.

Thus, while it is widely accepted that isolating and expanding tumor-specific T cells is a critical goal in cancer immunotherapy - something our results did not specifically achieved - future studies should focus on enriching these cells while ensuring the preservation of HLA-DR expression to maximize therapeutic efficacy.

Furthermore, determining the minimal effective percentage of HLA-DR+CTLs required for robust anti-tumor responses will necessitate further titration experiments and functional assays, which we also recognize as a limitation of our current study.

Importantly, the rationale of our proposed anti-PD-1 strategy does not depend on tumor PD-L1 expression, since anti-PD-1 is for *ex vivo* treatment aiming to enhance the cytotoxicity of T lymphocytes for potential T cell–based therapies. Therefore, PD-L1 negativity does not limit the applicability of this approach.

Nonetheless, while further investigation is needed, these findings pave the way for the development of improved personalized therapeutic interventions with patients’ own immune cells. Ultimately, the proposed approach holds the potential to reshape the landscape of BC treatments, thereby contributing to increased BC-free survival rates.

## Data Availability

The original contributions presented in the study are included in the article/Supplementary Material, further inquiries can be directed to the corresponding author/s.
