# Supplementary material for: HLA-DR expression in cytotoxic T lymphocytes: a key to boost the therapeutic potential of T cell-based strategies for breast cancer
Source: Front Immunol. 2025 Sep 19;16:1653970. doi: 10.3389/fimmu.2025.1653970 (PMC12491829; doi:10.3389/fimmu.2025.1653970)
Supplement: Supplementary file 1 [file DataSheet1.docx]

***Supplementary Material***

**
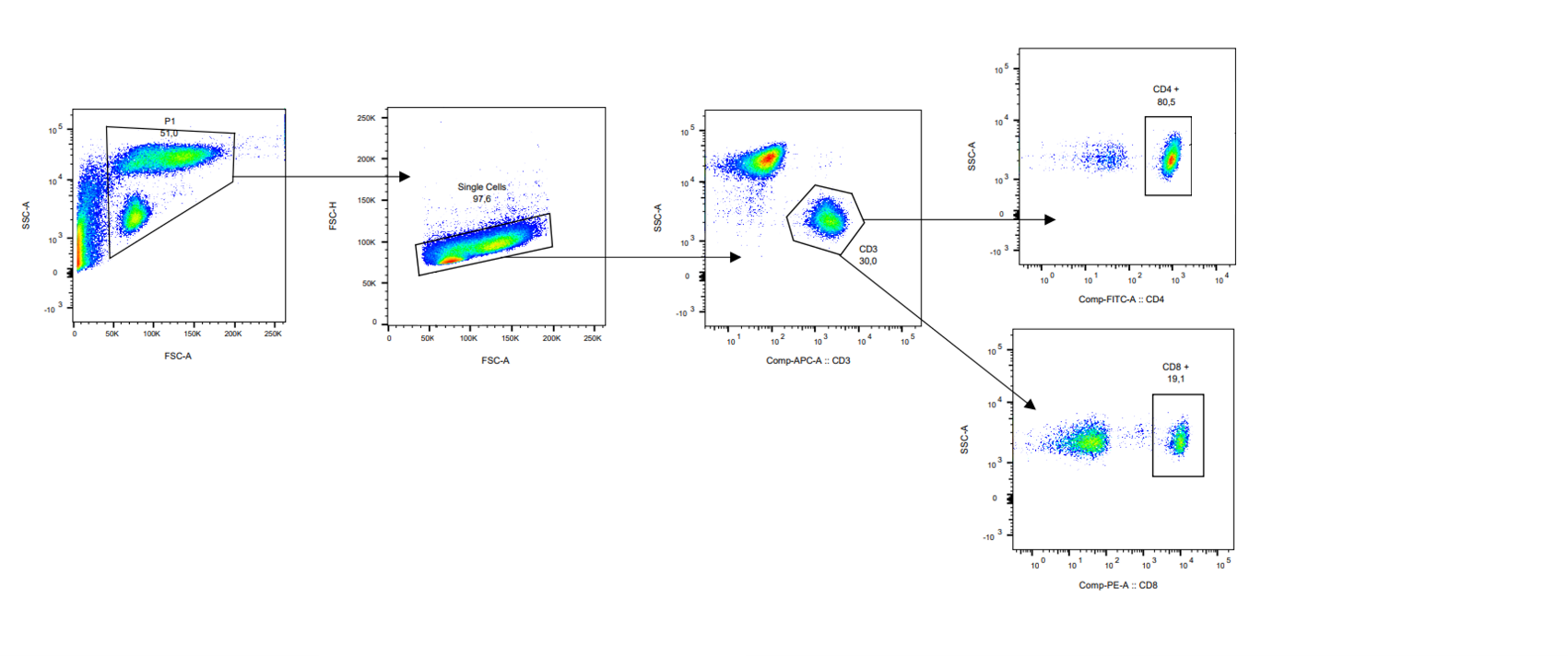
**

**Figure S1 - Gating strategy for immunophenotyping of blood and biopsy samples using monoclonal antibodies (mAbs).** The analysis identifies immune cell populations, including cytotoxic T lymphocytes (CD3+/CD8+) and CD4+ T cells (CD3+/CD4+). Positive and negative populations were always selected taken into consideration the negative (unstained) sample.

**
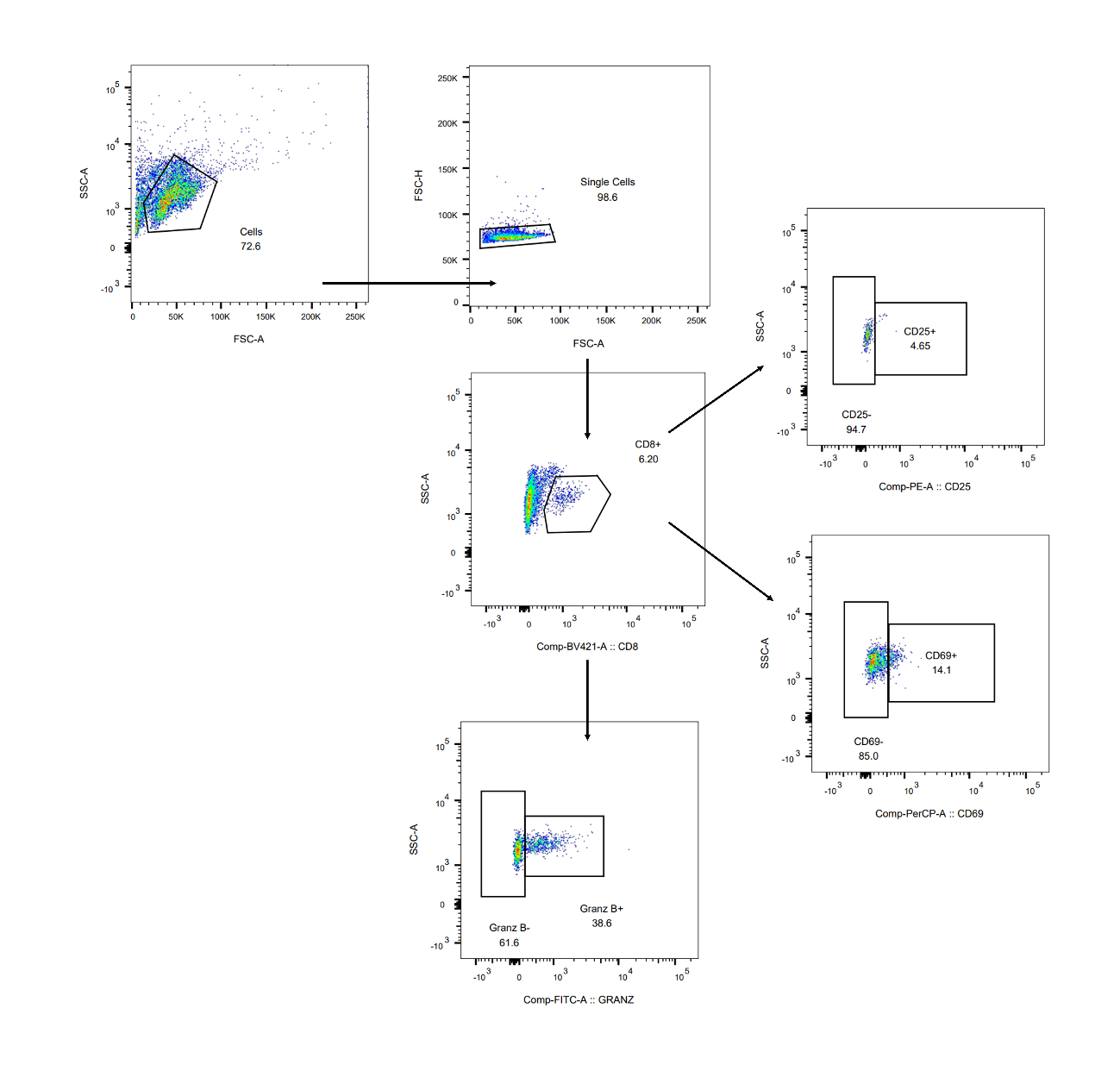
Figure S2 - Gating strategy for CTLs activation and cytotoxic function post HLA-DR blockade.** This figure illustrates the flow cytometry gating strategy used to evaluate CTLs activation (CD69, CD25) and cytotoxic potential (Granzyme B) after a 72-hour culture of PBMCs under unstimulated, stimulated, and HLA-DR-blocked conditions.
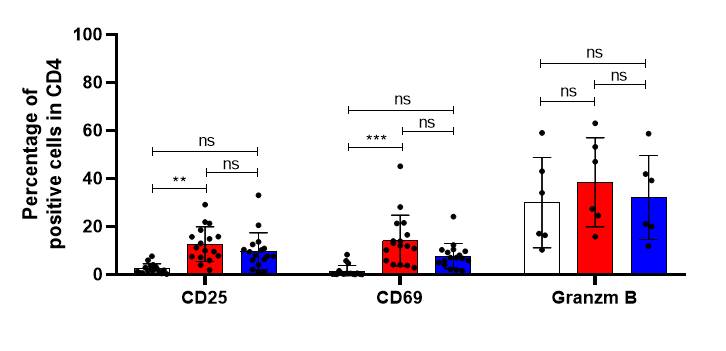


**Figure S3 - CD4 T cell activation and cytotoxicity remain unchanged upon HLA-DR blockade during stimulation.** CD4 T cells activation and cytotoxicity following stimulation, through CD3/CD28 co-stimulation, were assessed by measuring the percentage of CD25⁺ (n=17), CD69⁺ (n=17), and Granzyme B⁺ (n=6) cells within this population. PBMCs were either stimulated (red bars) or left unstimulated (white bars) to evaluate the effect on CD4+ T cells. An additional condition included stimulation in the presence of HLA-DR blockade (blue bars), to assess its potential influence on activation and cytotoxicity. Data are presented as mean ± SD. ***p < 0.01, *p < 0.001, ns = not significant*.


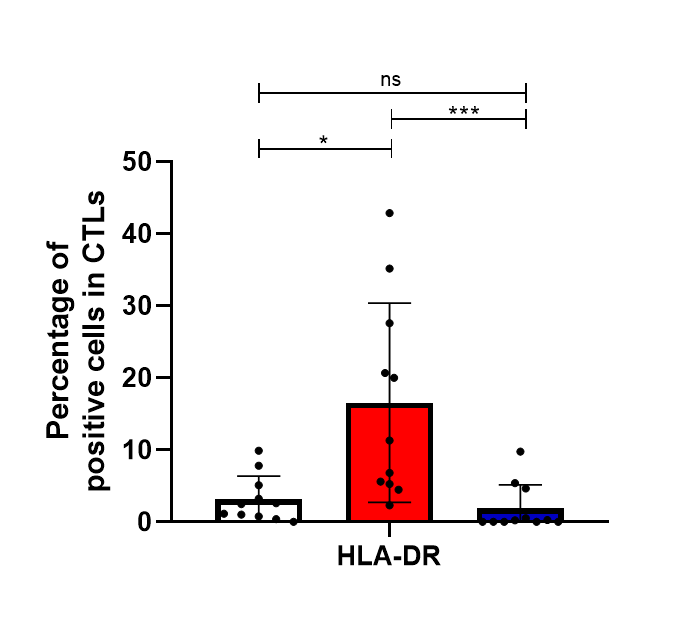


**Figure S4 – HLA-DR blockade reduces the frequency of HLA-DR+CTLs.** PBMCs unstimulated (n=11, white bar), stimulated via CD3/CD28 co-stimulation (n=11, red bar), or stimulated in the presence of an HLA-DR blocking antibody (n=11, blue bar). Data are represented as mean ± SD, **p < 0.05, ***p < 0.001, ns= non-statistical.*

**Figure S**
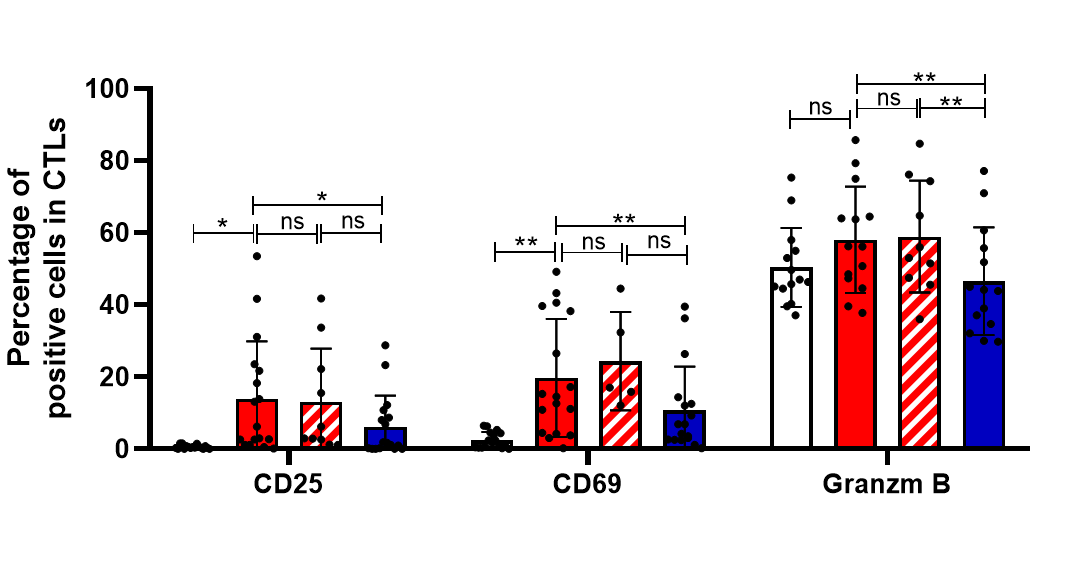
**5 -** **Isotype control assessment confirms the specificity of anti-HLA-DR antibody effects on T cell activation and cytotoxicity.** CTLs activation following stimulation was evaluated by measuring the percentage of CD69⁺ and CD25⁺ cells within this population. Cytotoxicity was assessed through Granzyme B expression. PBMCs were either stimulated (red bars) or maintained in non-stimulatory conditions (white bars) to evaluate CTL activation and cytotoxicity. Additional conditions included stimulation in the presence of HLA-DR blockade (blue bars) and stimulation with an isotype control antibody (red striped bars). The number of samples analyzed was n=19 for CD25 and CD69, and n=14 for Granzyme B. For the isotype control conditions (red striped bars), the sample size was n=12 for CD25 and granzyme B, and n=5 for CD69. Data are represented as mean ± SD, **p < 0.05, **p < 0.01, ns= non-statistical.*


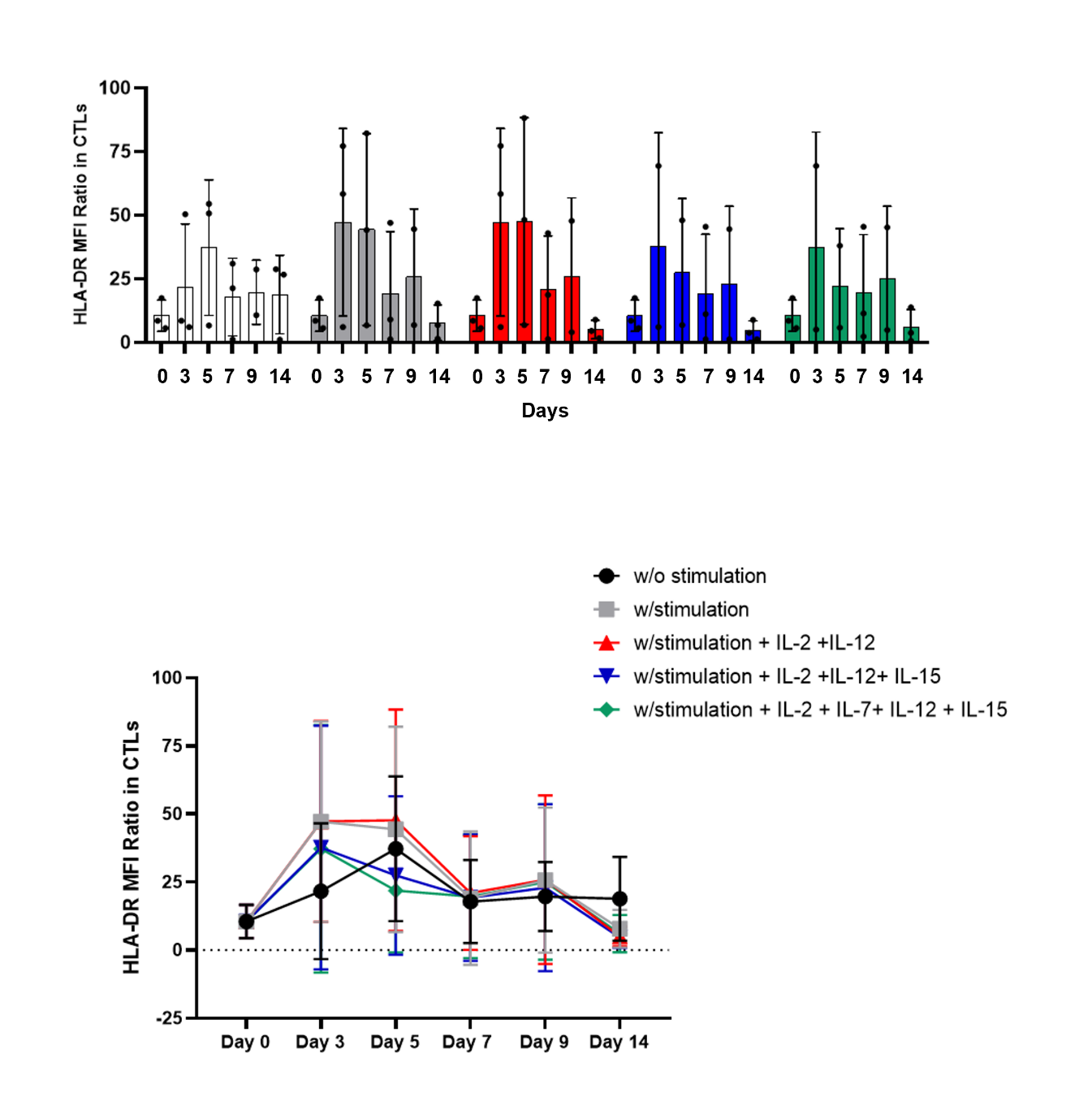


**Figure S6 - HLA-DR expression in CTLs during *ex vivo* PBMCs expansion.** PBMCs were cultured for up to 14 days under different stimulation conditions: unstimulated (black line); anti-CD3 (5 μg/mL) plus anti-CD28 (1 μg/mL) (grey line); stimulation with IL-2, IL-7, IL-12, and IL-15 (green line); IL-2, IL-12, and IL-15 (blue line); and IL-2 plus IL-12 (red line). Data represent n = 3 independent donors. HLA-DR expression in CTLs, assessed by mean fluorescence intensity (MFI), fluctuates over time, showing a tendency to peak around days 4–5, independent of the cytokine combination.

**
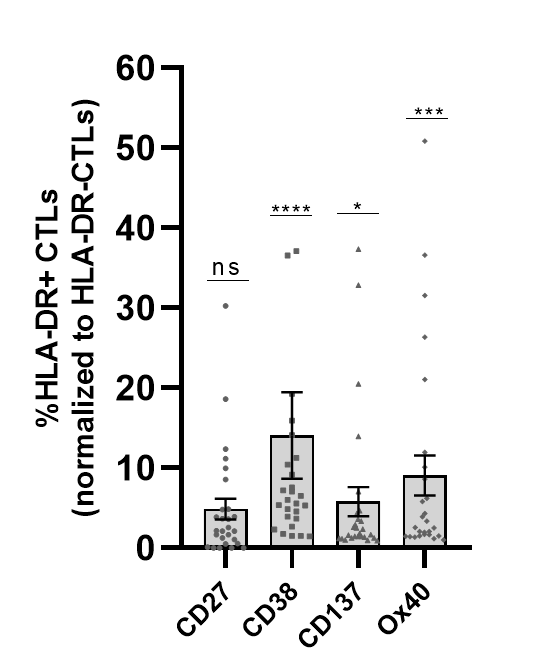
**

**Figure S7 - HLA-DR+ CTLs exhibit a distinct immune profile compared to HLA-DRneg CTLs.** % of HLA-DR+CTLs that also express the stimulatory co-receptors CD27, CD38, CD137 and Ox40, related to the % of HLA-DRneg CTLs that also express those markers (n=27). Data are represented as ± SEM, **p < 0.05,* ****p < 0.001, ****p < 0.0001, ns= non-statistical*

**
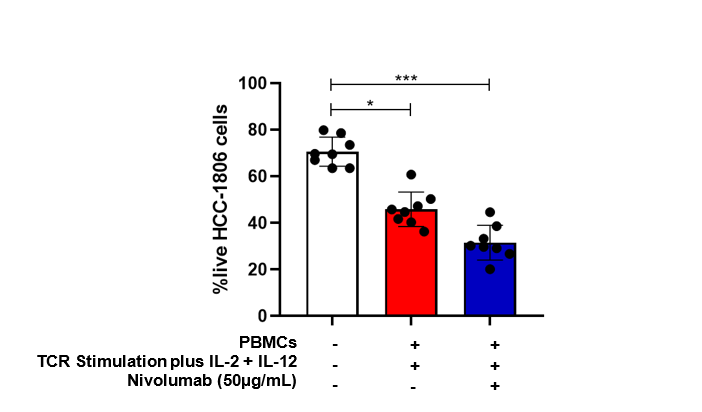
**

**Figure S8 – Anti-PD-1 treatment potentiates the anti-tumor capacity of CTLs against the HCC-1806 BC cell line.** The viability of the HCC-1806 BC cell line in the 3D co-culture alone (white bar, n=8), incubated in the presence of stimulated PBMCs (red bar, n=8, p=0.0486) and incubated in the presence of stimulated PBMCs plus the addition of the anti-PD-1, Nivolumab (blue bar, n=8, p<0.0001). Data are represented as mean ± SD, **p < 0.05, ***p < 0.001.*


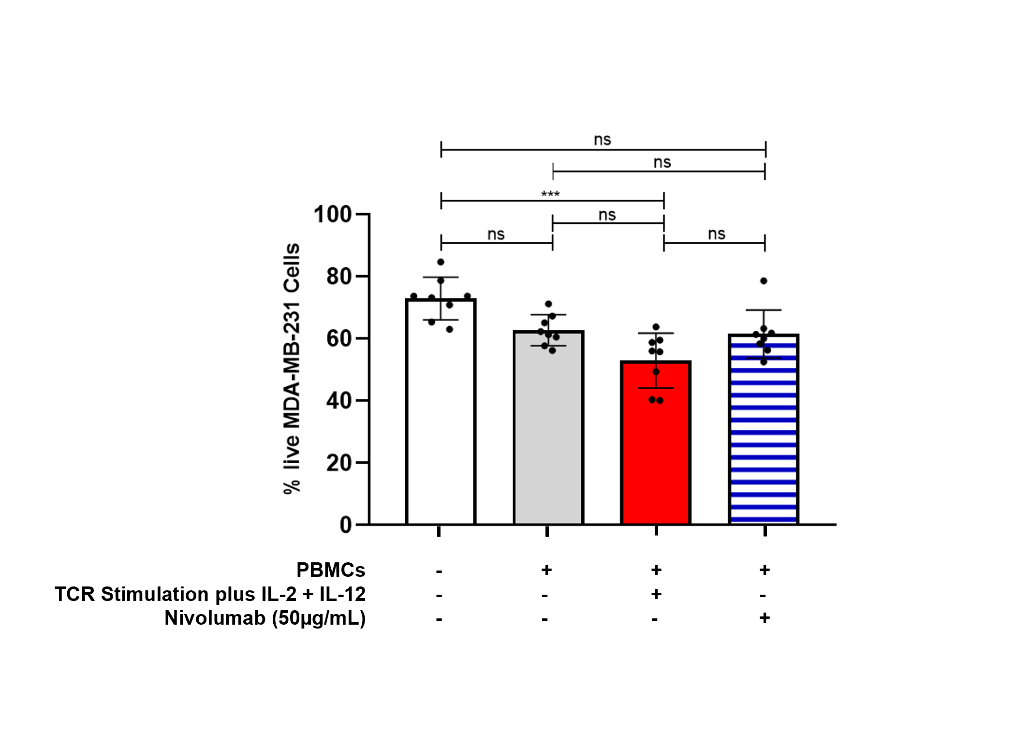


**Figure S9 – Pre-activation of T cells is essential for Nivolumab-mediated cytotoxicity.** The viability of the MDA-MD-231 BC cell line in 3D co-culture was assessed under different conditions: BC cells alone (white bar, n=8), incubated with unstimulated PBMCs (grey bar, n=8), with stimulated PBMCs (red bar, n=8), and with Nivolumab added in the absence of prior PBMCs stimulation (blue striped bar, n=8). Data are represented as mean ± SD, ****p < 0.001, ns= non-statistical.*

**
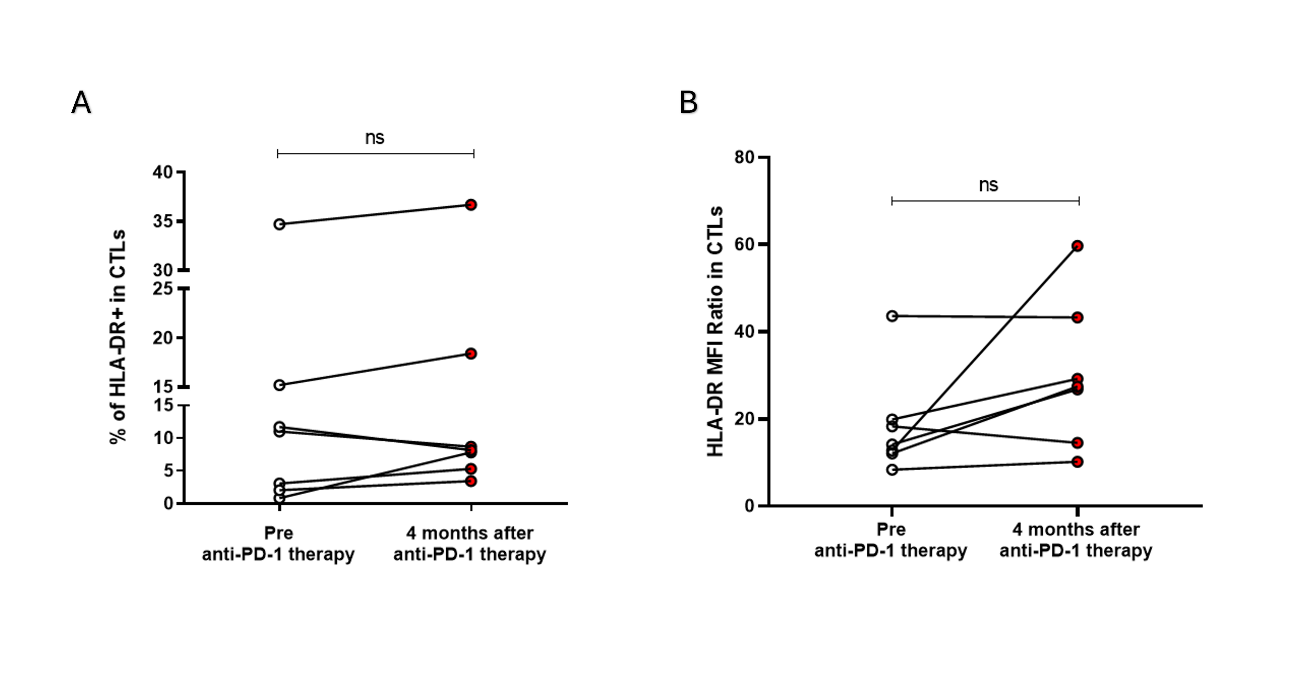
Figure S10 – BC patients undergoing anti-PD-1 therapy exhibited an increased frequency and expression of HLA-DR on CTLs in their blood. (A)** The percentage of CTLs expressing HLA-DR was measured at baseline and 4 months after initiation of anti-PD-1 therapy. **(B)** Mean Fluorescence Intensity (MFI) of HLA-DR expression on CTLs was assessed over the same time points. Each line represents one patient under anti-PD-1 therapy (n=7). *ns= non-statistical*.

**
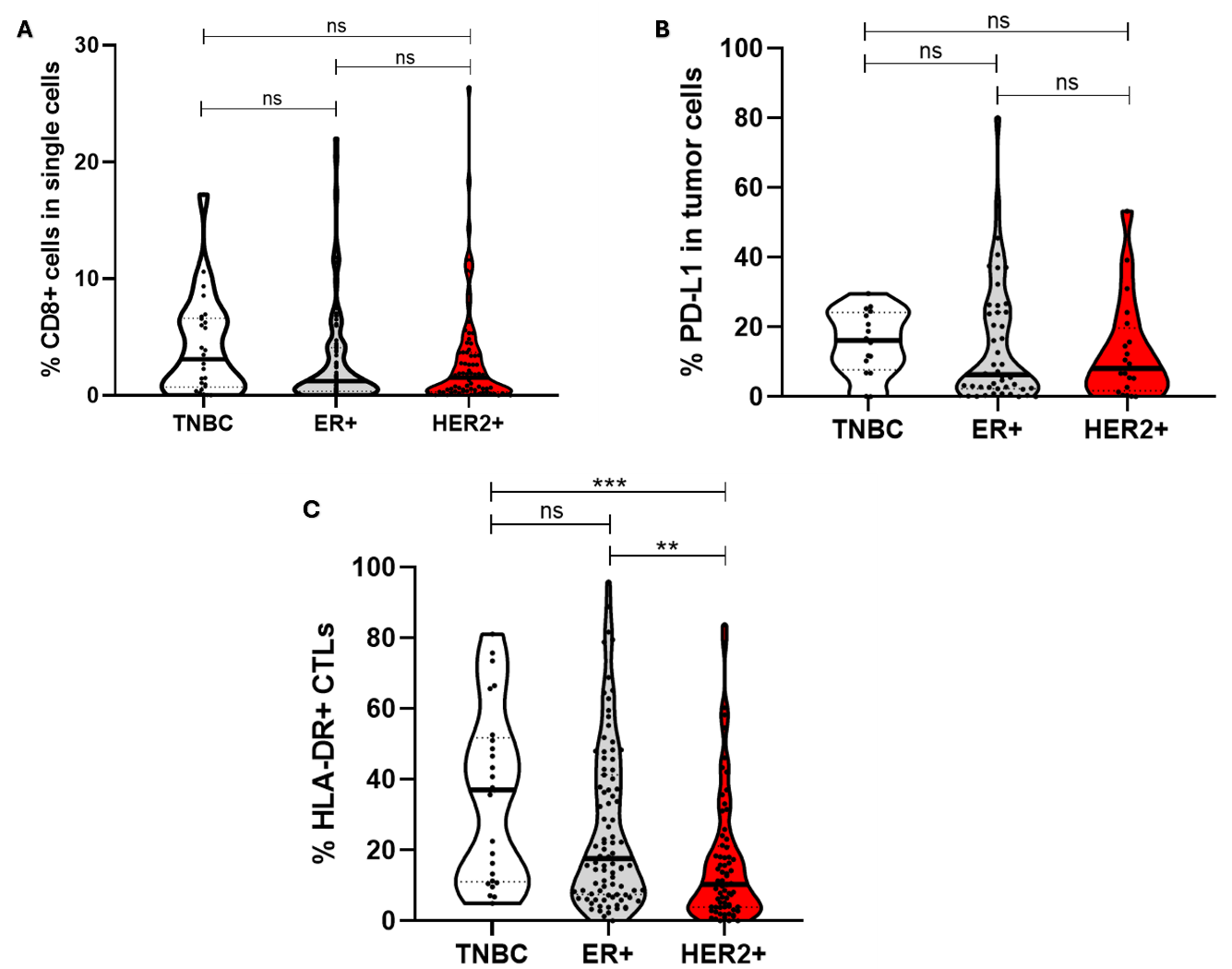
**

**Figure S11 – Similar frequencies of infiltrating CTLs and PD-L1 expression across the three main BC subtypes.** Flow cytometry analysis was performed on biopsies from a cohort of 195 breast cancer patients to evaluate immune parameters. The three main subtypes analyzed were triple-negative breast cancer (TNBC), HER2-positive (HER2+), and Luminal A and B (ER+). (**A)** Percentage of infiltrating CTLs was measured in TNBC (n=26), HER2+ (n=74), and ER+(n=95).  **(B)** Percentage of tumor cells expressing PD-L1 was assessed in TNBC (n=16), HER2+ (n=20) and ER+ (n=47). **(C)** Percentage of HLA-DR-expressing infiltrating CTLs was evaluated in TNBC cases (n=25, p= 0.0003) in comparison with HER2+ (n=69) and ER+ (n=91). Data are represented as mean ± SD, ***p < 0.01, ***p < 0.001,* *ns= non-statistical*.

**Table S1 –** Summary of the drugs tested, and the BC cell lines used in a small-size screening developed in the 3D platform established by our team^22^, aiming to identify candidates that enhance HLA-DR expression in CTLs, and therefore their cytotoxic capacity against the BC cells. Each candidate entry corresponds to a specific agent assessed in the study. The "BC Cell Line" column specifies the particular BC cell line against which agents were tested, evaluating their promise and effectiveness. The “Characteristics of BC cell line” column recaps the representativeness of BC subtype and the expression of PD-L1 in the respective cell lines used in the study.

| BC  cell line | Characteristics of  BC cell line (59) | Candidates: | | | |
| --- | --- | --- | --- | --- | --- |
|  |  | **Nivolumab**  **(Anti PD-1)** | **Bevacizumab**  **(Anti VEGF)** | **Anti CD137** | **Anti CD134** |
| **MDA**  **MB 231** | - Triple-negative BC - High Expression of   PD-L1 | 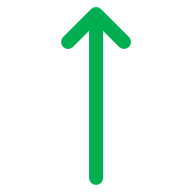 | 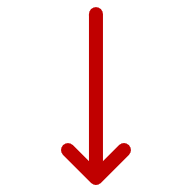 | 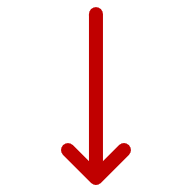 | 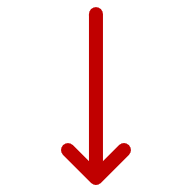 |
| **MCF-7** | - Luminal A (ER+ PR+) - Low Expression of   PD-L1 | 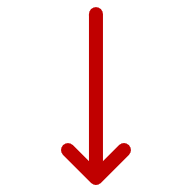 | 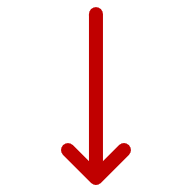 | 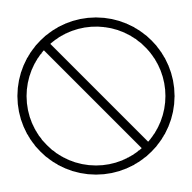 | 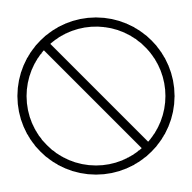 |
| **Hs578T** | - Triple-negative BC - Middle Expression of   PD-L1 | 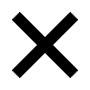 | 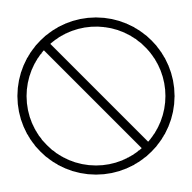 | 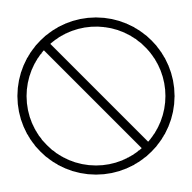 | 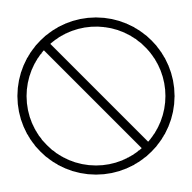 |
| **BT-474** | - HER2+ - Low Expression of   PD-L1 | 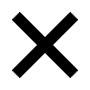 | 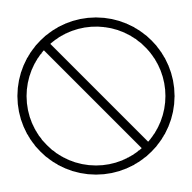 | 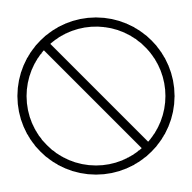 | 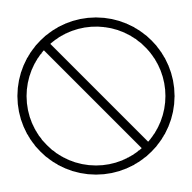 |
| **HCC**  **1806** | - Triple-negative BC - High Expression of   PD-L1 | 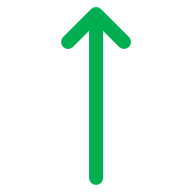 | 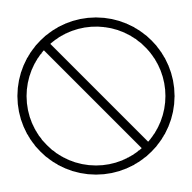 | 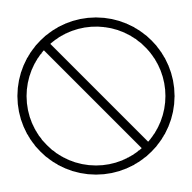 | 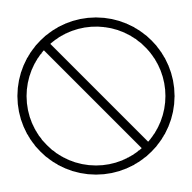 |

Candidate exhibits anti-tumor capabilities.


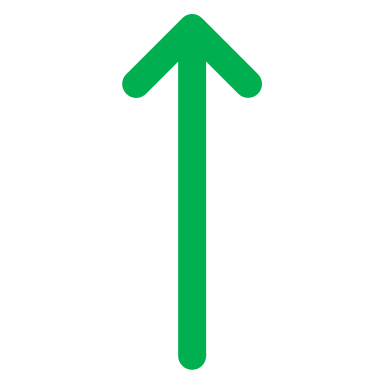

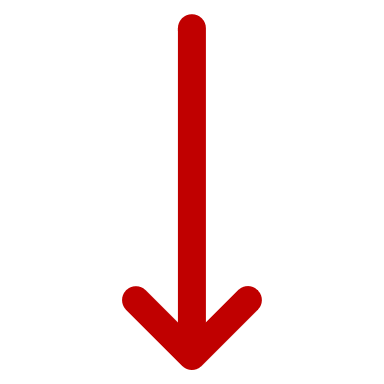

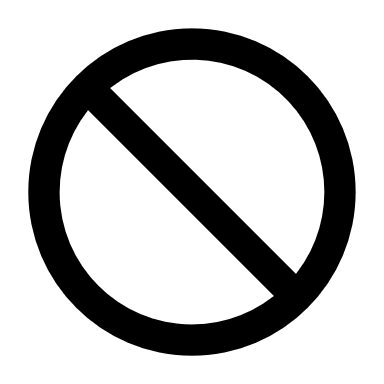

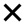


Candidate fails to demonstrate anti-tumor properties.

Candidate's anti-tumor capabilities remain untested.

Low spheroid viability or inadequate immune cell penetration into spheroids hampered the assessment of candidate anti-tumor efficacy.

**Table S2 –** List of genes significantly correlated with HLA-DR from BRCA_GSE150660 database

| Gene | Gene 2 | Cell Type | Correlation value |
| --- | --- | --- | --- |
| HLA-DRA | CD74 | CD8T | 0.778 |
| HLA-DRA | GZMH | CD8T | 0.714 |
| HLA-DRA | CCL4 | CD8T | 0.71 |
| HLA-DRA | CST7 | CD8T | 0.685 |
| HLA-DRA | CCR5 | CD8T | 0.646 |
| HLA-DRA | TMSB4X | CD8T | 0.643 |
| HLA-DRA | CCL5 | CD8T | 0.623 |
| HLA-DRA | EOMES | CD8T | 0.603 |
| HLA-DRA | GZMA | CD8T | 0.597 |
| HLA-DRA | HIGD2A | CD8T | 0.58 |
| HLA-DRA | CD81 | CD8T | 0.574 |
| HLA-DRA | SERF2 | CD8T | 0.567 |
| HLA-DRA | ITGB2 | CD8T | 0.554 |
| HLA-DRA | GZMK | CD8T | 0.549 |
| HLA-DRA | OAZ1 | CD8T | 0.549 |
| HLA-DRA | COTL1 | CD8T | 0.549 |
| HLA-DRA | CD8B | CD8T | 0.542 |
| HLA-DRA | NKG7 | CD8T | 0.53 |
| HLA-DRA | EIF1 | CD8T | 0.521 |
| HLA-DRA | CD8A | CD8T | 0.515 |
| HLA-DRA | PLEK | CD8T | 0.512 |

**Table S3 –** List of genes significantly correlated with HLA-DR from BRCA_GSE110686 database

| Gene | Gene 2 | Cell Type | Correlation value |
| --- | --- | --- | --- |
| HLA-DRA | CD74 | CD8T | 0.884 |
| HLA-DRA | CXCL13 | CD8T | 0.878 |
| HLA-DRA | LSP1 | CD8T | 0.872 |
| HLA-DRA | CCL3 | CD8T | 0.871 |
| HLA-DRA | GZMB | CD8T | 0.866 |
| HLA-DRA | HAVCR2 | CD8T | 0.864 |
| HLA-DRA | CCL4 | CD8T | 0.861 |
| HLA-DRA | TIGIT | CD8T | 0.859 |
| HLA-DRA | FABP5 | CD8T | 0.857 |
| HLA-DRA | DUSP4 | CD8T | 0.854 |
| HLA-DRA | PTMS | CD8T | 0.852 |
| HLA-DRA | GZMH | CD8T | 0.85 |
| HLA-DRA | LAG3 | CD8T | 0.839 |
| HLA-DRA | ANXA5 | CD8T | 0.835 |
| HLA-DRA | PRF1 | CD8T | 0.832 |
| HLA-DRA | ID2 | CD8T | 0.832 |
| HLA-DRA | GAPDH | CD8T | 0.829 |
| HLA-DRA | CXCR6 | CD8T | 0.816 |
| HLA-DRA | AC069363.1 | CD8T | 0.813 |
| HLA-DRA | VCAM1 | CD8T | 0.812 |
| HLA-DRA | FKBP1A | CD8T | 0.806 |
| HLA-DRA | GZMA | CD8T | 0.806 |
| HLA-DRA | PDCD1 | CD8T | 0.801 |
| HLA-DRA | PTTG1 | CD8T | 0.8 |
| HLA-DRA | SIRPG | CD8T | 0.799 |
| HLA-DRA | CD27 | CD8T | 0.798 |
| HLA-DRA | ACP5 | CD8T | 0.797 |
| HLA-DRA | COTL1 | CD8T | 0.796 |
| HLA-DRA | GNLY | CD8T | 0.795 |
| HLA-DRA | DYNLL1 | CD8T | 0.795 |
| HLA-DRA | PARK7 | CD8T | 0.794 |
| HLA-DRA | MIR155HG | CD8T | 0.792 |
| HLA-DRA | NKG7 | CD8T | 0.791 |
| HLA-DRA | YWHAH | CD8T | 0.789 |
| HLA-DRA | FASLG | CD8T | 0.788 |
| HLA-DRA | RABAC1 | CD8T | 0.786 |
| HLA-DRA | IFNG | CD8T | 0.78 |
| HLA-DRA | APOBEC3G | CD8T | 0.778 |
| HLA-DRA | CLEC2B | CD8T | 0.771 |
| HLA-DRA | CCR5 | CD8T | 0.767 |
| HLA-DRA | CST7 | CD8T | 0.767 |
| HLA-DRA | CD8A | CD8T | 0.764 |
| HLA-DRA | RHOA | CD8T | 0.761 |
| HLA-DRA | CSF1 | CD8T | 0.759 |
| HLA-DRA | APOBEC3C | CD8T | 0.758 |
| HLA-DRA | RGS2 | CD8T | 0.757 |
| HLA-DRA | SH2D1A | CD8T | 0.757 |
| HLA-DRA | SIT1 | CD8T | 0.756 |
| HLA-DRA | SUMO2 | CD8T | 0.755 |
| HLA-DRA | PHLDA1 | CD8T | 0.753 |
| HLA-DRA | TNFRSF9 | CD8T | 0.749 |
| HLA-DRA | TRAC | CD8T | 0.749 |
| HLA-DRA | SYNGR2 | CD8T | 0.748 |
| HLA-DRA | ABI3 | CD8T | 0.748 |
| HLA-DRA | AC133644.2 | CD8T | 0.745 |
| HLA-DRA | IDH2 | CD8T | 0.741 |
| HLA-DRA | CLIC1 | CD8T | 0.739 |
| HLA-DRA | IFI27L2 | CD8T | 0.737 |
| HLA-DRA | ALOX5AP | CD8T | 0.73 |
| HLA-DRA | PSMB3 | CD8T | 0.729 |
| HLA-DRA | CCR1 | CD8T | 0.725 |
| HLA-DRA | ITM2A | CD8T | 0.719 |
| HLA-DRA | SCAMP2 | CD8T | 0.718 |
| HLA-DRA | KLRC1 | CD8T | 0.711 |
| HLA-DRA | CD2 | CD8T | 0.709 |
| HLA-DRA | PTPN6 | CD8T | 0.709 |
| HLA-DRA | LITAF | CD8T | 0.709 |
| HLA-DRA | ITGB1 | CD8T | 0.708 |
| HLA-DRA | CD8B | CD8T | 0.707 |
| HLA-DRA | BHLHE40 | CD8T | 0.706 |
| HLA-DRA | UCP2 | CD8T | 0.705 |
| HLA-DRA | IVNS1ABP | CD8T | 0.705 |
| HLA-DRA | CHST12 | CD8T | 0.705 |
| HLA-DRA | DBI | CD8T | 0.704 |
| HLA-DRA | SRI | CD8T | 0.704 |
| HLA-DRA | MT1E | CD8T | 0.699 |
| HLA-DRA | LGALS1 | CD8T | 0.698 |
| HLA-DRA | LAYN | CD8T | 0.694 |
| HLA-DRA | COPZ1 | CD8T | 0.693 |
| HLA-DRA | SUB1 | CD8T | 0.69 |
| HLA-DRA | CAP1 | CD8T | 0.687 |
| HLA-DRA | SH2D2A | CD8T | 0.686 |
| HLA-DRA | EZR | CD8T | 0.682 |
| HLA-DRA | TXNDC17 | CD8T | 0.682 |
| HLA-DRA | CCND2 | CD8T | 0.678 |
| HLA-DRA | PGAM1 | CD8T | 0.678 |
| HLA-DRA | BATF | CD8T | 0.677 |
| HLA-DRA | IFI27 | CD8T | 0.675 |
| HLA-DRA | MYL6 | CD8T | 0.675 |
| HLA-DRA | HERPUD1 | CD8T | 0.674 |
| HLA-DRA | CD63 | CD8T | 0.673 |
| HLA-DRA | NRBP1 | CD8T | 0.672 |
| HLA-DRA | CAPZB | CD8T | 0.67 |
| HLA-DRA | PKM | CD8T | 0.669 |
| HLA-DRA | CKS2 | CD8T | 0.669 |
| HLA-DRA | CMC1 | CD8T | 0.668 |
| HLA-DRA | HNRNPLL | CD8T | 0.668 |
| HLA-DRA | TRBC1 | CD8T | 0.666 |
| HLA-DRA | CTSC | CD8T | 0.666 |
| HLA-DRA | HNRNPA3 | CD8T | 0.664 |
| HLA-DRA | SOX4 | CD8T | 0.664 |
| HLA-DRA | RHOC | CD8T | 0.663 |
| HLA-DRA | ID3 | CD8T | 0.663 |
| HLA-DRA | IGFLR1 | CD8T | 0.661 |
| HLA-DRA | CDK2AP2 | CD8T | 0.659 |
| HLA-DRA | CD3D | CD8T | 0.659 |
| HLA-DRA | TRAPPC1 | CD8T | 0.658 |
| HLA-DRA | ARPC2 | CD8T | 0.658 |
| HLA-DRA | SH3BGRL3 | CD8T | 0.657 |
| HLA-DRA | CARD16 | CD8T | 0.656 |
| HLA-DRA | AIF1 | CD8T | 0.656 |
| HLA-DRA | BUB3 | CD8T | 0.655 |
| HLA-DRA | PRDX6 | CD8T | 0.652 |
| HLA-DRA | COMMD7 | CD8T | 0.649 |
| HLA-DRA | CD82 | CD8T | 0.646 |
| HLA-DRA | HMGB1 | CD8T | 0.641 |
| HLA-DRA | ZBED2 | CD8T | 0.64 |
| HLA-DRA | CD79A | CD8T | 0.64 |
| HLA-DRA | MT1X | CD8T | 0.638 |
| HLA-DRA | SRGN | CD8T | 0.637 |
| HLA-DRA | SERF2 | CD8T | 0.634 |
| HLA-DRA | CCL5 | CD8T | 0.631 |
| HLA-DRA | COX5A | CD8T | 0.631 |
| HLA-DRA | SRP14 | CD8T | 0.63 |
| HLA-DRA | RBX1 | CD8T | 0.629 |
| HLA-DRA | MCM5 | CD8T | 0.629 |
| HLA-DRA | SLA | CD8T | 0.626 |
| HLA-DRA | CD2BP2 | CD8T | 0.626 |
| HLA-DRA | HSPB1 | CD8T | 0.625 |
| HLA-DRA | ENSA | CD8T | 0.623 |
| HLA-DRA | SCP2 | CD8T | 0.623 |
| HLA-DRA | ADGRE5 | CD8T | 0.623 |
| HLA-DRA | GALM | CD8T | 0.622 |
| HLA-DRA | HMGN3 | CD8T | 0.621 |
| HLA-DRA | TPI1 | CD8T | 0.618 |
| HLA-DRA | HSBP1 | CD8T | 0.618 |
| HLA-DRA | HNRNPK | CD8T | 0.616 |
| HLA-DRA | NDUFC1 | CD8T | 0.615 |
| HLA-DRA | DOK2 | CD8T | 0.613 |
| HLA-DRA | BSG | CD8T | 0.613 |
| HLA-DRA | KLRC2 | CD8T | 0.612 |
| HLA-DRA | VCP | CD8T | 0.612 |
| HLA-DRA | TMED9 | CD8T | 0.609 |
| HLA-DRA | PSMB2 | CD8T | 0.608 |
| HLA-DRA | CTLA4 | CD8T | 0.608 |
| HLA-DRA | POMP | CD8T | 0.608 |
| HLA-DRA | UBB | CD8T | 0.607 |
| HLA-DRA | CFL1 | CD8T | 0.607 |
| HLA-DRA | SOD1 | CD8T | 0.606 |
| HLA-DRA | NDUFB3 | CD8T | 0.606 |
| HLA-DRA | LCK | CD8T | 0.605 |
| HLA-DRA | CALM3 | CD8T | 0.603 |
| HLA-DRA | KIF20B | CD8T | 0.602 |
| HLA-DRA | CTSW | CD8T | 0.602 |
| HLA-DRA | KRT86 | CD8T | 0.601 |
| HLA-DRA | BAX | CD8T | 0.6 |
| HLA-DRA | CALM2 | CD8T | 0.599 |
| HLA-DRA | ARPP19 | CD8T | 0.599 |
| HLA-DRA | PSMC4 | CD8T | 0.598 |
| HLA-DRA | RGS1 | CD8T | 0.598 |
| HLA-DRA | BIN1 | CD8T | 0.597 |
| HLA-DRA | CLIC3 | CD8T | 0.595 |
| HLA-DRA | NUDT5 | CD8T | 0.594 |
| HLA-DRA | IL2RG | CD8T | 0.594 |
| HLA-DRA | CARHSP1 | CD8T | 0.593 |
| HLA-DRA | PCMT1 | CD8T | 0.593 |
| HLA-DRA | PYCARD | CD8T | 0.592 |
| HLA-DRA | TNFRSF1B | CD8T | 0.592 |
| HLA-DRA | H2AFZ | CD8T | 0.588 |
| HLA-DRA | OAZ1 | CD8T | 0.588 |
| HLA-DRA | MCL1 | CD8T | 0.587 |
| HLA-DRA | SAMSN1 | CD8T | 0.586 |
| HLA-DRA | NELFCD | CD8T | 0.585 |
| HLA-DRA | SNRPD1 | CD8T | 0.585 |
| HLA-DRA | PSMA7 | CD8T | 0.583 |
| HLA-DRA | PPDPF | CD8T | 0.583 |
| HLA-DRA | B3GNT2 | CD8T | 0.583 |
| HLA-DRA | PRDX5 | CD8T | 0.583 |
| HLA-DRA | CCDC28B | CD8T | 0.582 |
| HLA-DRA | NUSAP1 | CD8T | 0.582 |
| HLA-DRA | SURF4 | CD8T | 0.581 |
| HLA-DRA | AP2S1 | CD8T | 0.58 |
| HLA-DRA | TMEM165 | CD8T | 0.58 |
| HLA-DRA | AIP | CD8T | 0.578 |
| HLA-DRA | SKA2 | CD8T | 0.577 |
| HLA-DRA | ATP6V1E1 | CD8T | 0.576 |
| HLA-DRA | PSMA1 | CD8T | 0.575 |
| HLA-DRA | UBE2A | CD8T | 0.573 |
| HLA-DRA | EOMES | CD8T | 0.573 |
| HLA-DRA | PDIA3 | CD8T | 0.572 |
| HLA-DRA | TANK | CD8T | 0.571 |
| HLA-DRA | CD247 | CD8T | 0.57 |
| HLA-DRA | COX8A | CD8T | 0.57 |
| HLA-DRA | DNAJC8 | CD8T | 0.569 |
| HLA-DRA | ACTB | CD8T | 0.567 |
| HLA-DRA | CLEC2D | CD8T | 0.565 |
| HLA-DRA | UBE2L3 | CD8T | 0.564 |
| HLA-DRA | YWHAQ | CD8T | 0.561 |
| HLA-DRA | FERMT3 | CD8T | 0.56 |
| HLA-DRA | YWHAE | CD8T | 0.558 |
| HLA-DRA | SLBP | CD8T | 0.558 |
| HLA-DRA | EID1 | CD8T | 0.558 |
| HLA-DRA | CORO1A | CD8T | 0.557 |
| HLA-DRA | CD3E | CD8T | 0.556 |
| HLA-DRA | PRR13 | CD8T | 0.555 |
| HLA-DRA | RNF167 | CD8T | 0.554 |
| HLA-DRA | PFN1 | CD8T | 0.554 |
| HLA-DRA | TMCO1 | CD8T | 0.554 |
| HLA-DRA | BRK1 | CD8T | 0.554 |
| HLA-DRA | HSPB11 | CD8T | 0.554 |
| HLA-DRA | NAP1L4 | CD8T | 0.553 |
| HLA-DRA | SEC11A | CD8T | 0.552 |
| HLA-DRA | LARP7 | CD8T | 0.551 |
| HLA-DRA | THYN1 | CD8T | 0.551 |
| HLA-DRA | PDIA6 | CD8T | 0.551 |
| HLA-DRA | HM13 | CD8T | 0.55 |
| HLA-DRA | ARPC3 | CD8T | 0.55 |
| HLA-DRA | PMF1 | CD8T | 0.55 |
| HLA-DRA | DNPH1 | CD8T | 0.548 |
| HLA-DRA | CMTM3 | CD8T | 0.548 |
| HLA-DRA | SRSF9 | CD8T | 0.547 |
| HLA-DRA | PSMB9 | CD8T | 0.547 |
| HLA-DRA | ITM2C | CD8T | 0.547 |
| HLA-DRA | RAB11B | CD8T | 0.546 |
| HLA-DRA | CTSD | CD8T | 0.546 |
| HLA-DRA | ARPC1B | CD8T | 0.545 |
| HLA-DRA | PPP4C | CD8T | 0.544 |
| HLA-DRA | UBL5 | CD8T | 0.544 |
| HLA-DRA | PRKAR1A | CD8T | 0.543 |
| HLA-DRA | LINC01480 | CD8T | 0.543 |
| HLA-DRA | LBH | CD8T | 0.542 |
| HLA-DRA | MRPS16 | CD8T | 0.541 |
| HLA-DRA | HMGN1 | CD8T | 0.541 |
| HLA-DRA | LAMTOR5 | CD8T | 0.54 |
| HLA-DRA | BLOC1S1 | CD8T | 0.539 |
| HLA-DRA | KXD1 | CD8T | 0.538 |
| HLA-DRA | GSTP1 | CD8T | 0.538 |
| HLA-DRA | TMEM179B | CD8T | 0.538 |
| HLA-DRA | AKR1B1 | CD8T | 0.538 |
| HLA-DRA | HSD17B10 | CD8T | 0.537 |
| HLA-DRA | SAP18 | CD8T | 0.537 |
| HLA-DRA | SLC1A5 | CD8T | 0.536 |
| HLA-DRA | PEF1 | CD8T | 0.536 |
| HLA-DRA | MT1F | CD8T | 0.534 |
| HLA-DRA | RHBDD2 | CD8T | 0.531 |
| HLA-DRA | SERPINB9 | CD8T | 0.53 |
| HLA-DRA | ACTG1 | CD8T | 0.53 |
| HLA-DRA | NDUFV2 | CD8T | 0.529 |
| HLA-DRA | ARHGEF1 | CD8T | 0.528 |
| HLA-DRA | HMGN2 | CD8T | 0.528 |
| HLA-DRA | MRPS34 | CD8T | 0.527 |
| HLA-DRA | EIF4E | CD8T | 0.527 |
| HLA-DRA | CYB5B | CD8T | 0.527 |
| HLA-DRA | PSMD4 | CD8T | 0.526 |
| HLA-DRA | ASNA1 | CD8T | 0.526 |
| HLA-DRA | CYCS | CD8T | 0.526 |
| HLA-DRA | PSMB1 | CD8T | 0.523 |
| HLA-DRA | COX6A1 | CD8T | 0.522 |
| HLA-DRA | NDUFA13 | CD8T | 0.522 |
| HLA-DRA | SHKBP1 | CD8T | 0.522 |
| HLA-DRA | PTPN7 | CD8T | 0.522 |
| HLA-DRA | RASAL3 | CD8T | 0.521 |
| HLA-DRA | B2M | CD8T | 0.521 |
| HLA-DRA | AP2M1 | CD8T | 0.521 |
| HLA-DRA | TXN | CD8T | 0.521 |
| HLA-DRA | ARL6IP1 | CD8T | 0.52 |
| HLA-DRA | RNF181 | CD8T | 0.52 |
| HLA-DRA | PSMD8 | CD8T | 0.519 |
| HLA-DRA | FKBP8 | CD8T | 0.519 |
| HLA-DRA | SMC4 | CD8T | 0.519 |
| HLA-DRA | COMMD3 | CD8T | 0.518 |
| HLA-DRA | CALR | CD8T | 0.517 |
| HLA-DRA | PSMD7 | CD8T | 0.517 |
| HLA-DRA | COPE | CD8T | 0.516 |
| HLA-DRA | SNU13 | CD8T | 0.516 |
| HLA-DRA | TMX1 | CD8T | 0.516 |
| HLA-DRA | FDPS | CD8T | 0.514 |
| HLA-DRA | SEC61G | CD8T | 0.513 |
| HLA-DRA | PLEKHJ1 | CD8T | 0.513 |
| HLA-DRA | ITGB2 | CD8T | 0.513 |
| HLA-DRA | ITGB7 | CD8T | 0.513 |
| HLA-DRA | TMEM59 | CD8T | 0.513 |
| HLA-DRA | PHPT1 | CD8T | 0.513 |
| HLA-DRA | HNRNPC | CD8T | 0.513 |
| HLA-DRA | CAPZA1 | CD8T | 0.512 |
| HLA-DRA | TSPO | CD8T | 0.512 |
| HLA-DRA | CYBA | CD8T | 0.511 |

**Table S4 –** List of genes significantly correlated with HLA-DR from BRCA_GSE114727_10X database

| Gene | Gene 2 | Cell Type | Correlation value |
| --- | --- | --- | --- |
| HLA-DRA | GZMH | CD8T | 0.864 |
| HLA-DRA | CTSC | CD8T | 0.84 |
| HLA-DRA | ANXA5 | CD8T | 0.828 |
| HLA-DRA | FABP5 | CD8T | 0.82 |
| HLA-DRA | APOBEC3G | CD8T | 0.819 |
| HLA-DRA | SUB1 | CD8T | 0.817 |
| HLA-DRA | IDH2 | CD8T | 0.81 |
| HLA-DRA | LSP1 | CD8T | 0.808 |
| HLA-DRA | APOBEC3C | CD8T | 0.806 |
| HLA-DRA | PTTG1 | CD8T | 0.803 |
| HLA-DRA | CAP1 | CD8T | 0.794 |
| HLA-DRA | COTL1 | CD8T | 0.788 |
| HLA-DRA | PSMB9 | CD8T | 0.78 |
| HLA-DRA | LAG3 | CD8T | 0.777 |
| HLA-DRA | PTMS | CD8T | 0.773 |
| HLA-DRA | ARPC2 | CD8T | 0.765 |
| HLA-DRA | CAPZB | CD8T | 0.765 |
| HLA-DRA | PPP1CA | CD8T | 0.763 |
| HLA-DRA | CXCR6 | CD8T | 0.761 |
| HLA-DRA | CD74 | CD8T | 0.755 |
| HLA-DRA | EVL | CD8T | 0.753 |
| HLA-DRA | POMP | CD8T | 0.752 |
| HLA-DRA | ALOX5AP | CD8T | 0.751 |
| HLA-DRA | RHOA | CD8T | 0.75 |
| HLA-DRA | CLIC1 | CD8T | 0.744 |
| HLA-DRA | PDCD1 | CD8T | 0.744 |
| HLA-DRA | CARD16 | CD8T | 0.743 |
| HLA-DRA | CTSD | CD8T | 0.739 |
| HLA-DRA | IFNG | CD8T | 0.737 |
| HLA-DRA | TPM3 | CD8T | 0.737 |
| HLA-DRA | PRF1 | CD8T | 0.736 |
| HLA-DRA | HMGB1 | CD8T | 0.734 |
| HLA-DRA | COX5A | CD8T | 0.732 |
| HLA-DRA | set/07 | CD8T | 0.732 |
| HLA-DRA | LCP1 | CD8T | 0.73 |
| HLA-DRA | GZMA | CD8T | 0.728 |
| HLA-DRA | PYCARD | CD8T | 0.726 |
| HLA-DRA | CD2 | CD8T | 0.725 |
| HLA-DRA | DUSP4 | CD8T | 0.725 |
| HLA-DRA | RBCK1 | CD8T | 0.722 |
| HLA-DRA | ZYX | CD8T | 0.722 |
| HLA-DRA | LCK | CD8T | 0.721 |
| HLA-DRA | GAPDH | CD8T | 0.721 |
| HLA-DRA | PSTPIP1 | CD8T | 0.72 |
| HLA-DRA | ANXA6 | CD8T | 0.718 |
| HLA-DRA | CCL3 | CD8T | 0.718 |
| HLA-DRA | PSME2 | CD8T | 0.717 |
| HLA-DRA | UBE2L3 | CD8T | 0.717 |
| HLA-DRA | ACTB | CD8T | 0.716 |
| HLA-DRA | RAB1B | CD8T | 0.714 |
| HLA-DRA | PARK7 | CD8T | 0.714 |
| HLA-DRA | CCR5 | CD8T | 0.714 |
| HLA-DRA | REEP5 | CD8T | 0.712 |
| HLA-DRA | IFI16 | CD8T | 0.71 |
| HLA-DRA | WDR1 | CD8T | 0.708 |
| HLA-DRA | MAP4K1 | CD8T | 0.707 |
| HLA-DRA | ARPC3 | CD8T | 0.706 |
| HLA-DRA | MSC | CD8T | 0.706 |
| HLA-DRA | CFL1 | CD8T | 0.705 |
| HLA-DRA | RABAC1 | CD8T | 0.704 |
| HLA-DRA | DBI | CD8T | 0.703 |
| HLA-DRA | BATF | CD8T | 0.702 |
| HLA-DRA | DYNLRB1 | CD8T | 0.702 |
| HLA-DRA | PSMB3 | CD8T | 0.702 |
| HLA-DRA | SHISA5 | CD8T | 0.702 |
| HLA-DRA | CDK2AP2 | CD8T | 0.702 |
| HLA-DRA | YWHAB | CD8T | 0.701 |
| HLA-DRA | MSN | CD8T | 0.7 |
| HLA-DRA | UBE2L6 | CD8T | 0.7 |
| HLA-DRA | GNG5 | CD8T | 0.7 |
| HLA-DRA | IFI27L2 | CD8T | 0.699 |
| HLA-DRA | BTG3 | CD8T | 0.699 |
| HLA-DRA | ADGRE5 | CD8T | 0.698 |
| HLA-DRA | HSPB11 | CD8T | 0.697 |
| HLA-DRA | PDIA6 | CD8T | 0.697 |
| HLA-DRA | CD3D | CD8T | 0.695 |
| HLA-DRA | GZMB | CD8T | 0.694 |
| HLA-DRA | ITGAE | CD8T | 0.694 |
| HLA-DRA | FERMT3 | CD8T | 0.691 |
| HLA-DRA | LY6E | CD8T | 0.69 |
| HLA-DRA | COPE | CD8T | 0.69 |
| HLA-DRA | GNAS | CD8T | 0.689 |
| HLA-DRA | CALR | CD8T | 0.689 |
| HLA-DRA | MYO1G | CD8T | 0.688 |
| HLA-DRA | B2M | CD8T | 0.687 |
| HLA-DRA | WAS | CD8T | 0.686 |
| HLA-DRA | ENSA | CD8T | 0.686 |
| HLA-DRA | PSMB8 | CD8T | 0.686 |
| HLA-DRA | PSMB10 | CD8T | 0.685 |
| HLA-DRA | ACTR3 | CD8T | 0.685 |
| HLA-DRA | AKIRIN2 | CD8T | 0.685 |
| HLA-DRA | PSMD8 | CD8T | 0.684 |
| HLA-DRA | EZR | CD8T | 0.683 |
| HLA-DRA | JAML | CD8T | 0.683 |
| HLA-DRA | MIR155HG | CD8T | 0.683 |
| HLA-DRA | SH3BP1 | CD8T | 0.683 |
| HLA-DRA | PPIB | CD8T | 0.679 |
| HLA-DRA | LCP2 | CD8T | 0.679 |
| HLA-DRA | ITM2A | CD8T | 0.678 |
| HLA-DRA | CORO1A | CD8T | 0.678 |
| HLA-DRA | ANXA2 | CD8T | 0.678 |
| HLA-DRA | CSK | CD8T | 0.677 |
| HLA-DRA | DYNLL1 | CD8T | 0.677 |
| HLA-DRA | MYH9 | CD8T | 0.676 |
| HLA-DRA | BIN1 | CD8T | 0.676 |
| HLA-DRA | SEC11A | CD8T | 0.676 |
| HLA-DRA | GPI | CD8T | 0.675 |
| HLA-DRA | RALY | CD8T | 0.675 |
| HLA-DRA | MYL6 | CD8T | 0.674 |
| HLA-DRA | COPZ1 | CD8T | 0.674 |
| HLA-DRA | TBCB | CD8T | 0.672 |
| HLA-DRA | CHST12 | CD8T | 0.672 |
| HLA-DRA | CYBA | CD8T | 0.671 |
| HLA-DRA | RAC1 | CD8T | 0.671 |
| HLA-DRA | TAP1 | CD8T | 0.67 |
| HLA-DRA | PKM | CD8T | 0.669 |
| HLA-DRA | PSMA7 | CD8T | 0.669 |
| HLA-DRA | PIN1 | CD8T | 0.669 |
| HLA-DRA | COMMD7 | CD8T | 0.669 |
| HLA-DRA | TWF2 | CD8T | 0.667 |
| HLA-DRA | ASNA1 | CD8T | 0.666 |
| HLA-DRA | HNRNPA3 | CD8T | 0.666 |
| HLA-DRA | PSMA6 | CD8T | 0.666 |
| HLA-DRA | MRPL28 | CD8T | 0.666 |
| HLA-DRA | TXN | CD8T | 0.665 |
| HLA-DRA | RBX1 | CD8T | 0.665 |
| HLA-DRA | IL2RG | CD8T | 0.665 |
| HLA-DRA | PPP4C | CD8T | 0.664 |
| HLA-DRA | CASP4 | CD8T | 0.664 |
| HLA-DRA | CCL4 | CD8T | 0.664 |
| HLA-DRA | YWHAZ | CD8T | 0.662 |
| HLA-DRA | UBE2A | CD8T | 0.662 |
| HLA-DRA | BSG | CD8T | 0.661 |
| HLA-DRA | APBB1IP | CD8T | 0.66 |
| HLA-DRA | YWHAQ | CD8T | 0.659 |
| HLA-DRA | TNFRSF1B | CD8T | 0.658 |
| HLA-DRA | RAB11B | CD8T | 0.657 |
| HLA-DRA | UBE2D3 | CD8T | 0.657 |
| HLA-DRA | ARPC5L | CD8T | 0.657 |
| HLA-DRA | PSMB1 | CD8T | 0.656 |
| HLA-DRA | PLEKHF1 | CD8T | 0.656 |
| HLA-DRA | MDH2 | CD8T | 0.656 |
| HLA-DRA | TLN1 | CD8T | 0.656 |
| HLA-DRA | HCLS1 | CD8T | 0.656 |
| HLA-DRA | CD53 | CD8T | 0.653 |
| HLA-DRA | CD3G | CD8T | 0.653 |
| HLA-DRA | CDCA7 | CD8T | 0.652 |
| HLA-DRA | PSMB6 | CD8T | 0.652 |
| HLA-DRA | TXNDC17 | CD8T | 0.651 |
| HLA-DRA | PPM1G | CD8T | 0.651 |
| HLA-DRA | PDZD11 | CD8T | 0.651 |
| HLA-DRA | DEK | CD8T | 0.651 |
| HLA-DRA | ACP5 | CD8T | 0.65 |
| HLA-DRA | SMC4 | CD8T | 0.649 |
| HLA-DRA | PSMB2 | CD8T | 0.649 |
| HLA-DRA | ARF1 | CD8T | 0.649 |
| HLA-DRA | MT2A | CD8T | 0.649 |
| HLA-DRA | SRGN | CD8T | 0.648 |
| HLA-DRA | PGAM1 | CD8T | 0.645 |
| HLA-DRA | NKG7 | CD8T | 0.645 |
| HLA-DRA | GYG1 | CD8T | 0.644 |
| HLA-DRA | ABI3 | CD8T | 0.644 |
| HLA-DRA | PPM1M | CD8T | 0.643 |
| HLA-DRA | ARHGAP9 | CD8T | 0.643 |
| HLA-DRA | GBP5 | CD8T | 0.641 |
| HLA-DRA | ARHGDIA | CD8T | 0.641 |
| HLA-DRA | PSMA5 | CD8T | 0.641 |
| HLA-DRA | FASLG | CD8T | 0.641 |
| HLA-DRA | TRAPPC1 | CD8T | 0.639 |
| HLA-DRA | MT1F | CD8T | 0.639 |
| HLA-DRA | GNB2 | CD8T | 0.639 |
| HLA-DRA | ARF5 | CD8T | 0.639 |
| HLA-DRA | VAMP8 | CD8T | 0.638 |
| HLA-DRA | RNF167 | CD8T | 0.638 |
| HLA-DRA | CAPN2 | CD8T | 0.637 |
| HLA-DRA | AP2M1 | CD8T | 0.636 |
| HLA-DRA | CD63 | CD8T | 0.636 |
| HLA-DRA | CD99 | CD8T | 0.635 |
| HLA-DRA | PPP1R18 | CD8T | 0.634 |
| HLA-DRA | CALM3 | CD8T | 0.634 |
| HLA-DRA | PARP1 | CD8T | 0.634 |
| HLA-DRA | AC069363.1 | CD8T | 0.633 |
| HLA-DRA | TMEM120A | CD8T | 0.632 |
| HLA-DRA | OTUB1 | CD8T | 0.632 |
| HLA-DRA | CLTB | CD8T | 0.632 |
| HLA-DRA | TMEM50A | CD8T | 0.632 |
| HLA-DRA | RAC2 | CD8T | 0.632 |
| HLA-DRA | MIEN1 | CD8T | 0.632 |
| HLA-DRA | NDUFS6 | CD8T | 0.631 |
| HLA-DRA | PDIA3 | CD8T | 0.631 |
| HLA-DRA | BLOC1S1 | CD8T | 0.63 |
| HLA-DRA | SSBP4 | CD8T | 0.629 |
| HLA-DRA | SUMO2 | CD8T | 0.629 |
| HLA-DRA | FKBP8 | CD8T | 0.629 |
| HLA-DRA | BUB3 | CD8T | 0.628 |
| HLA-DRA | PPP1R7 | CD8T | 0.627 |
| HLA-DRA | NDUFA6 | CD8T | 0.627 |
| HLA-DRA | RARRES3 | CD8T | 0.627 |
| HLA-DRA | RNF213 | CD8T | 0.626 |
| HLA-DRA | ZCRB1 | CD8T | 0.625 |
| HLA-DRA | CTSW | CD8T | 0.625 |
| HLA-DRA | TBC1D10C | CD8T | 0.624 |
| HLA-DRA | SLC7A5 | CD8T | 0.624 |
| HLA-DRA | CLEC2B | CD8T | 0.623 |
| HLA-DRA | IL32 | CD8T | 0.623 |
| HLA-DRA | EWSR1 | CD8T | 0.622 |
| HLA-DRA | GTF3C6 | CD8T | 0.622 |
| HLA-DRA | VDAC1 | CD8T | 0.619 |
| HLA-DRA | AP2S1 | CD8T | 0.619 |
| HLA-DRA | RPN1 | CD8T | 0.619 |
| HLA-DRA | SRI | CD8T | 0.617 |
| HLA-DRA | ARPP19 | CD8T | 0.616 |
| HLA-DRA | TMEM59 | CD8T | 0.616 |
| HLA-DRA | NDUFB3 | CD8T | 0.616 |
| HLA-DRA | SH3BGRL | CD8T | 0.616 |
| HLA-DRA | ACTG1 | CD8T | 0.615 |
| HLA-DRA | F2R | CD8T | 0.613 |
| HLA-DRA | RNF181 | CD8T | 0.612 |
| HLA-DRA | MFSD10 | CD8T | 0.611 |
| HLA-DRA | SPCS2 | CD8T | 0.61 |
| HLA-DRA | PRDX1 | CD8T | 0.61 |
| HLA-DRA | SLC9A3R1 | CD8T | 0.609 |
| HLA-DRA | SURF4 | CD8T | 0.609 |
| HLA-DRA | UBE2N | CD8T | 0.609 |
| HLA-DRA | ITGB7 | CD8T | 0.609 |
| HLA-DRA | GLIPR1 | CD8T | 0.608 |
| HLA-DRA | ITGB2 | CD8T | 0.607 |
| HLA-DRA | SH3BGRL3 | CD8T | 0.607 |
| HLA-DRA | RGS1 | CD8T | 0.606 |
| HLA-DRA | CD52 | CD8T | 0.606 |
| HLA-DRA | RNF187 | CD8T | 0.606 |
| HLA-DRA | NUDT21 | CD8T | 0.605 |
| HLA-DRA | SP100 | CD8T | 0.605 |
| HLA-DRA | CALCOCO2 | CD8T | 0.605 |
| HLA-DRA | BRK1 | CD8T | 0.605 |
| HLA-DRA | RER1 | CD8T | 0.604 |
| HLA-DRA | CCDC167 | CD8T | 0.604 |
| HLA-DRA | FKBP1A | CD8T | 0.604 |
| HLA-DRA | EID1 | CD8T | 0.604 |
| HLA-DRA | ZBP1 | CD8T | 0.604 |
| HLA-DRA | LAMTOR5 | CD8T | 0.604 |
| HLA-DRA | SCAMP2 | CD8T | 0.603 |
| HLA-DRA | ORMDL2 | CD8T | 0.603 |
| HLA-DRA | DCTN3 | CD8T | 0.603 |
| HLA-DRA | ZNHIT1 | CD8T | 0.603 |
| HLA-DRA | AIP | CD8T | 0.603 |
| HLA-DRA | HSBP1 | CD8T | 0.602 |
| HLA-DRA | BUD31 | CD8T | 0.602 |
| HLA-DRA | GSDMD | CD8T | 0.602 |
| HLA-DRA | DRAP1 | CD8T | 0.602 |
| HLA-DRA | UBC | CD8T | 0.601 |
| HLA-DRA | SASH3 | CD8T | 0.601 |
| HLA-DRA | TPI1 | CD8T | 0.601 |
| HLA-DRA | HNRNPK | CD8T | 0.601 |
| HLA-DRA | UBB | CD8T | 0.601 |
| HLA-DRA | PSMC4 | CD8T | 0.601 |
| HLA-DRA | BST2 | CD8T | 0.601 |
| HLA-DRA | MYL12B | CD8T | 0.6 |
| HLA-DRA | MDH1 | CD8T | 0.6 |
| HLA-DRA | CHMP2A | CD8T | 0.6 |
| HLA-DRA | CPNE7 | CD8T | 0.598 |
| HLA-DRA | RNF7 | CD8T | 0.597 |
| HLA-DRA | PPP2R1A | CD8T | 0.597 |
| HLA-DRA | SLA | CD8T | 0.596 |
| HLA-DRA | TBCD | CD8T | 0.595 |
| HLA-DRA | ARL6IP5 | CD8T | 0.595 |
| HLA-DRA | MAGOH | CD8T | 0.595 |
| HLA-DRA | SEC11C | CD8T | 0.594 |
| HLA-DRA | VASP | CD8T | 0.594 |
| HLA-DRA | MEA1 | CD8T | 0.594 |
| HLA-DRA | ADRM1 | CD8T | 0.594 |
| HLA-DRA | DCTN2 | CD8T | 0.593 |
| HLA-DRA | ANP32A | CD8T | 0.593 |
| HLA-DRA | SLC1A5 | CD8T | 0.593 |
| HLA-DRA | GNAI2 | CD8T | 0.593 |
| HLA-DRA | IAH1 | CD8T | 0.593 |
| HLA-DRA | LPXN | CD8T | 0.592 |
| HLA-DRA | SSNA1 | CD8T | 0.592 |
| HLA-DRA | GUK1 | CD8T | 0.592 |
| HLA-DRA | COX6A1 | CD8T | 0.592 |
| HLA-DRA | SERPINB1 | CD8T | 0.592 |
| HLA-DRA | GSTP1 | CD8T | 0.592 |
| HLA-DRA | PSMA4 | CD8T | 0.592 |
| HLA-DRA | UBE2I | CD8T | 0.591 |
| HLA-DRA | AURKAIP1 | CD8T | 0.591 |
| HLA-DRA | KIF5B | CD8T | 0.591 |
| HLA-DRA | NELFCD | CD8T | 0.591 |
| HLA-DRA | JOSD2 | CD8T | 0.59 |
| HLA-DRA | CHMP1A | CD8T | 0.59 |
| HLA-DRA | TSPO | CD8T | 0.59 |
| HLA-DRA | ANAPC11 | CD8T | 0.59 |
| HLA-DRA | SHKBP1 | CD8T | 0.59 |
| HLA-DRA | PSMC3 | CD8T | 0.59 |
| HLA-DRA | UCP2 | CD8T | 0.589 |
| HLA-DRA | C11orf58 | CD8T | 0.589 |
| HLA-DRA | TMEM258 | CD8T | 0.589 |
| HLA-DRA | AP1S1 | CD8T | 0.589 |
| HLA-DRA | TNFRSF9 | CD8T | 0.588 |
| HLA-DRA | SRSF9 | CD8T | 0.588 |
| HLA-DRA | DR1 | CD8T | 0.588 |
| HLA-DRA | TYMP | CD8T | 0.587 |
| HLA-DRA | COMMD8 | CD8T | 0.587 |
| HLA-DRA | RASAL3 | CD8T | 0.586 |
| HLA-DRA | PSMD4 | CD8T | 0.586 |
| HLA-DRA | NDUFS7 | CD8T | 0.586 |
| HLA-DRA | PSME1 | CD8T | 0.586 |
| HLA-DRA | GBP2 | CD8T | 0.585 |
| HLA-DRA | OPTN | CD8T | 0.585 |
| HLA-DRA | SCAND1 | CD8T | 0.585 |
| HLA-DRA | POLD4 | CD8T | 0.584 |
| HLA-DRA | CCL5 | CD8T | 0.584 |
| HLA-DRA | ATP6V0E1 | CD8T | 0.584 |
| HLA-DRA | COX7A2 | CD8T | 0.584 |
| HLA-DRA | COX7B | CD8T | 0.583 |
| HLA-DRA | MPG | CD8T | 0.583 |
| HLA-DRA | CXCL13 | CD8T | 0.583 |
| HLA-DRA | MRPS36 | CD8T | 0.583 |
| HLA-DRA | RBM42 | CD8T | 0.583 |
| HLA-DRA | SNRPB | CD8T | 0.583 |
| HLA-DRA | GLUD1 | CD8T | 0.582 |
| HLA-DRA | CD2BP2 | CD8T | 0.582 |
| HLA-DRA | SNRPB2 | CD8T | 0.582 |
| HLA-DRA | PRR13 | CD8T | 0.582 |
| HLA-DRA | BAX | CD8T | 0.581 |
| HLA-DRA | VAMP5 | CD8T | 0.581 |
| HLA-DRA | CCL4L2 | CD8T | 0.58 |
| HLA-DRA | TSTA3 | CD8T | 0.58 |
| HLA-DRA | SIT1 | CD8T | 0.58 |
| HLA-DRA | TMEM179B | CD8T | 0.579 |
| HLA-DRA | FIBP | CD8T | 0.579 |
| HLA-DRA | TIGIT | CD8T | 0.579 |
| HLA-DRA | NDUFA1 | CD8T | 0.578 |
| HLA-DRA | SNRPG | CD8T | 0.576 |
| HLA-DRA | PUF60 | CD8T | 0.575 |
| HLA-DRA | WDR83OS | CD8T | 0.575 |
| HLA-DRA | NDUFB10 | CD8T | 0.575 |
| HLA-DRA | PGK1 | CD8T | 0.574 |
| HLA-DRA | SUMO1 | CD8T | 0.574 |
| HLA-DRA | TALDO1 | CD8T | 0.574 |
| HLA-DRA | LAMTOR2 | CD8T | 0.574 |
| HLA-DRA | SNX6 | CD8T | 0.574 |
| HLA-DRA | GOLGA7 | CD8T | 0.573 |
| HLA-DRA | COX8A | CD8T | 0.573 |
| HLA-DRA | POLR2G | CD8T | 0.573 |
| HLA-DRA | LGALS1 | CD8T | 0.572 |
| HLA-DRA | SELPLG | CD8T | 0.572 |
| HLA-DRA | TIMM8B | CD8T | 0.572 |
| HLA-DRA | VPS28 | CD8T | 0.572 |
| HLA-DRA | CASP1 | CD8T | 0.571 |
| HLA-DRA | NAA38 | CD8T | 0.571 |
| HLA-DRA | SEC61B | CD8T | 0.571 |
| HLA-DRA | ITGB1BP1 | CD8T | 0.57 |
| HLA-DRA | CMTM3 | CD8T | 0.57 |
| HLA-DRA | PFN1 | CD8T | 0.57 |
| HLA-DRA | TCIRG1 | CD8T | 0.569 |
| HLA-DRA | MIIP | CD8T | 0.569 |
| HLA-DRA | UBL5 | CD8T | 0.569 |
| HLA-DRA | PET100 | CD8T | 0.568 |
| HLA-DRA | IL2RB | CD8T | 0.567 |
| HLA-DRA | YWHAH | CD8T | 0.567 |
| HLA-DRA | SF3B6 | CD8T | 0.567 |
| HLA-DRA | TMEM14A | CD8T | 0.566 |
| HLA-DRA | XRCC5 | CD8T | 0.566 |
| HLA-DRA | CTLA4 | CD8T | 0.566 |
| HLA-DRA | C4orf48 | CD8T | 0.566 |
| HLA-DRA | CALM2 | CD8T | 0.566 |
| HLA-DRA | ANXA11 | CD8T | 0.564 |
| HLA-DRA | PDCD6 | CD8T | 0.564 |
| HLA-DRA | GABARAPL1 | CD8T | 0.564 |
| HLA-DRA | TADA3 | CD8T | 0.563 |
| HLA-DRA | TPM4 | CD8T | 0.563 |
| HLA-DRA | LSM2 | CD8T | 0.562 |
| HLA-DRA | ILK | CD8T | 0.562 |
| HLA-DRA | TRAC | CD8T | 0.562 |
| HLA-DRA | HCST | CD8T | 0.562 |
| HLA-DRA | ARRDC1 | CD8T | 0.562 |
| HLA-DRA | TMX4 | CD8T | 0.562 |
| HLA-DRA | HMGN2 | CD8T | 0.561 |
| HLA-DRA | CDC42 | CD8T | 0.56 |
| HLA-DRA | CKS2 | CD8T | 0.56 |
| HLA-DRA | SEC61G | CD8T | 0.56 |
| HLA-DRA | HSD17B10 | CD8T | 0.56 |
| HLA-DRA | MOB1A | CD8T | 0.559 |
| HLA-DRA | CST7 | CD8T | 0.559 |
| HLA-DRA | OASL | CD8T | 0.558 |
| HLA-DRA | LSM8 | CD8T | 0.558 |
| HLA-DRA | SAMD9L | CD8T | 0.557 |
| HLA-DRA | MPC2 | CD8T | 0.556 |
| HLA-DRA | CD8A | CD8T | 0.556 |
| HLA-DRA | ROMO1 | CD8T | 0.556 |
| HLA-DRA | MAD2L2 | CD8T | 0.556 |
| HLA-DRA | PAIP2 | CD8T | 0.555 |
| HLA-DRA | RAB7A | CD8T | 0.555 |
| HLA-DRA | IFI35 | CD8T | 0.555 |
| HLA-DRA | COX6B1 | CD8T | 0.555 |
| HLA-DRA | MAP1LC3B | CD8T | 0.555 |
| HLA-DRA | ISG15 | CD8T | 0.554 |
| HLA-DRA | SAP18 | CD8T | 0.554 |
| HLA-DRA | ITGB1 | CD8T | 0.554 |
| HLA-DRA | CFLAR | CD8T | 0.553 |
| HLA-DRA | ARHGAP30 | CD8T | 0.553 |
| HLA-DRA | SF3B2 | CD8T | 0.553 |
| HLA-DRA | DUSP23 | CD8T | 0.552 |
| HLA-DRA | NABP2 | CD8T | 0.552 |
| HLA-DRA | SDF2L1 | CD8T | 0.552 |
| HLA-DRA | NDUFAB1 | CD8T | 0.551 |
| HLA-DRA | SH2D2A | CD8T | 0.551 |
| HLA-DRA | RAP1B | CD8T | 0.551 |
| HLA-DRA | CAPZA1 | CD8T | 0.55 |
| HLA-DRA | CD3E | CD8T | 0.55 |
| HLA-DRA | APOBEC3H | CD8T | 0.55 |
| HLA-DRA | PTPN7 | CD8T | 0.549 |
| HLA-DRA | EIF6 | CD8T | 0.549 |
| HLA-DRA | LIME1 | CD8T | 0.549 |
| HLA-DRA | NDUFS3 | CD8T | 0.549 |
| HLA-DRA | TRIM22 | CD8T | 0.548 |
| HLA-DRA | AC133644.2 | CD8T | 0.548 |
| HLA-DRA | WIPF1 | CD8T | 0.547 |
| HLA-DRA | PSMC1 | CD8T | 0.546 |
| HLA-DRA | COX17 | CD8T | 0.546 |
| HLA-DRA | NUDT5 | CD8T | 0.545 |
| HLA-DRA | SLTM | CD8T | 0.545 |
| HLA-DRA | SLC25A5 | CD8T | 0.545 |
| HLA-DRA | CHCHD2 | CD8T | 0.545 |
| HLA-DRA | NDUFB7 | CD8T | 0.544 |
| HLA-DRA | ENO1 | CD8T | 0.544 |
| HLA-DRA | S100A6 | CD8T | 0.544 |
| HLA-DRA | GSTK1 | CD8T | 0.543 |
| HLA-DRA | PHPT1 | CD8T | 0.543 |
| HLA-DRA | PRDX5 | CD8T | 0.543 |
| HLA-DRA | CCNDBP1 | CD8T | 0.543 |
| HLA-DRA | ARPC5 | CD8T | 0.543 |
| HLA-DRA | LDHA | CD8T | 0.542 |
| HLA-DRA | KDELR2 | CD8T | 0.542 |
| HLA-DRA | KRTCAP2 | CD8T | 0.542 |
| HLA-DRA | NDUFS8 | CD8T | 0.542 |
| HLA-DRA | ACTR2 | CD8T | 0.541 |
| HLA-DRA | NDUFA2 | CD8T | 0.541 |
| HLA-DRA | BTN3A2 | CD8T | 0.541 |
| HLA-DRA | C9orf16 | CD8T | 0.541 |
| HLA-DRA | BCL2L1 | CD8T | 0.541 |
| HLA-DRA | NDUFB2 | CD8T | 0.541 |
| HLA-DRA | TCEA1 | CD8T | 0.541 |
| HLA-DRA | GSTO1 | CD8T | 0.541 |
| HLA-DRA | PLEKHJ1 | CD8T | 0.54 |
| HLA-DRA | TMX1 | CD8T | 0.539 |
| HLA-DRA | NDUFC1 | CD8T | 0.539 |
| HLA-DRA | ITPA | CD8T | 0.539 |
| HLA-DRA | RHOG | CD8T | 0.539 |
| HLA-DRA | PSMA3 | CD8T | 0.538 |
| HLA-DRA | RAD21 | CD8T | 0.538 |
| HLA-DRA | DBNL | CD8T | 0.538 |
| HLA-DRA | ATP6V1F | CD8T | 0.538 |
| HLA-DRA | UQCR11 | CD8T | 0.538 |
| HLA-DRA | COX5B | CD8T | 0.538 |
| HLA-DRA | HNRNPF | CD8T | 0.538 |
| HLA-DRA | ATP6V0B | CD8T | 0.537 |
| HLA-DRA | DAD1 | CD8T | 0.536 |
| HLA-DRA | ATP6V0C | CD8T | 0.536 |
| HLA-DRA | UBE2D2 | CD8T | 0.536 |
| HLA-DRA | CHCHD5 | CD8T | 0.535 |
| HLA-DRA | FMNL1 | CD8T | 0.535 |
| HLA-DRA | ECHS1 | CD8T | 0.535 |
| HLA-DRA | GABARAP | CD8T | 0.534 |
| HLA-DRA | SYNGR2 | CD8T | 0.533 |
| HLA-DRA | LGALS3 | CD8T | 0.533 |
| HLA-DRA | CARHSP1 | CD8T | 0.533 |
| HLA-DRA | THRAP3 | CD8T | 0.533 |
| HLA-DRA | P4HB | CD8T | 0.533 |
| HLA-DRA | PLP2 | CD8T | 0.533 |
| HLA-DRA | YIPF3 | CD8T | 0.533 |
| HLA-DRA | CCND2 | CD8T | 0.532 |
| HLA-DRA | SIRPG | CD8T | 0.532 |
| HLA-DRA | HERPUD1 | CD8T | 0.532 |
| HLA-DRA | DEF6 | CD8T | 0.532 |
| HLA-DRA | MRPL41 | CD8T | 0.532 |
| HLA-DRA | FLNA | CD8T | 0.532 |
| HLA-DRA | VIM | CD8T | 0.532 |
| HLA-DRA | PRDX6 | CD8T | 0.532 |
| HLA-DRA | HNRNPLL | CD8T | 0.532 |
| HLA-DRA | EMC7 | CD8T | 0.532 |
| HLA-DRA | CISD3 | CD8T | 0.531 |
| HLA-DRA | H2AFZ | CD8T | 0.53 |
| HLA-DRA | CYB561D2 | CD8T | 0.53 |
| HLA-DRA | OAZ1 | CD8T | 0.529 |
| HLA-DRA | IDH3G | CD8T | 0.529 |
| HLA-DRA | H3F3B | CD8T | 0.529 |
| HLA-DRA | TMEM160 | CD8T | 0.528 |
| HLA-DRA | LAMTOR1 | CD8T | 0.528 |
| HLA-DRA | ELOVL1 | CD8T | 0.528 |
| HLA-DRA | ISG20 | CD8T | 0.528 |
| HLA-DRA | NUDT1 | CD8T | 0.528 |
| HLA-DRA | SNRPD3 | CD8T | 0.527 |
| HLA-DRA | TEX264 | CD8T | 0.527 |
| HLA-DRA | APOL6 | CD8T | 0.526 |
| HLA-DRA | CBX3 | CD8T | 0.526 |
| HLA-DRA | METTL9 | CD8T | 0.526 |
| HLA-DRA | FGFR1OP2 | CD8T | 0.526 |
| HLA-DRA | RHOF | CD8T | 0.526 |

**Table S5 –** List of genes significantly correlated with HLA-DR from BRCA_GSE161529 database

| Gene | Gene 2 | Cell Type | Correlation value |
| --- | --- | --- | --- |
| HLA-DRA | CD74 | CD8T | 0.961 |
| HLA-DRA | LAPTM5 | CD8T | 0.876 |
| HLA-DRA | CD53 | CD8T | 0.851 |
| HLA-DRA | EVI2B | CD8T | 0.832 |
| HLA-DRA | GPSM3 | CD8T | 0.823 |
| HLA-DRA | HCLS1 | CD8T | 0.813 |
| HLA-DRA | CTSS | CD8T | 0.808 |
| HLA-DRA | RGS1 | CD8T | 0.801 |
| HLA-DRA | NCF1 | CD8T | 0.796 |
| HLA-DRA | CYBB | CD8T | 0.787 |
| HLA-DRA | CD83 | CD8T | 0.786 |
| HLA-DRA | CD37 | CD8T | 0.786 |
| HLA-DRA | ITGB2 | CD8T | 0.783 |
| HLA-DRA | CTSH | CD8T | 0.78 |
| HLA-DRA | PLEK | CD8T | 0.778 |
| HLA-DRA | RNASE6 | CD8T | 0.777 |
| HLA-DRA | TYROBP | CD8T | 0.776 |
| HLA-DRA | AIF1 | CD8T | 0.773 |
| HLA-DRA | GMFG | CD8T | 0.772 |
| HLA-DRA | GPR183 | CD8T | 0.772 |
| HLA-DRA | C1orf162 | CD8T | 0.77 |
| HLA-DRA | FERMT3 | CD8T | 0.77 |
| HLA-DRA | LCP1 | CD8T | 0.768 |
| HLA-DRA | SPI1 | CD8T | 0.768 |
| HLA-DRA | LGALS9 | CD8T | 0.765 |
| HLA-DRA | HCST | CD8T | 0.761 |
| HLA-DRA | LST1 | CD8T | 0.761 |
| HLA-DRA | PTPN6 | CD8T | 0.76 |
| HLA-DRA | TAGAP | CD8T | 0.758 |
| HLA-DRA | CARD16 | CD8T | 0.756 |
| HLA-DRA | ARHGDIB | CD8T | 0.755 |
| HLA-DRA | PTPRC | CD8T | 0.755 |
| HLA-DRA | FCER1G | CD8T | 0.75 |
| HLA-DRA | LYZ | CD8T | 0.749 |
| HLA-DRA | MS4A6A | CD8T | 0.747 |
| HLA-DRA | IL10RA | CD8T | 0.744 |
| HLA-DRA | CD84 | CD8T | 0.744 |
| HLA-DRA | MNDA | CD8T | 0.744 |
| HLA-DRA | SRGN | CD8T | 0.742 |
| HLA-DRA | CD48 | CD8T | 0.74 |
| HLA-DRA | LAT2 | CD8T | 0.74 |
| HLA-DRA | FYB1 | CD8T | 0.738 |
| HLA-DRA | LPXN | CD8T | 0.736 |
| HLA-DRA | LAIR1 | CD8T | 0.733 |
| HLA-DRA | FCGR2B | CD8T | 0.732 |
| HLA-DRA | C1QA | CD8T | 0.732 |
| HLA-DRA | GPR65 | CD8T | 0.732 |
| HLA-DRA | THEMIS2 | CD8T | 0.731 |
| HLA-DRA | LILRB1 | CD8T | 0.729 |
| HLA-DRA | RNASET2 | CD8T | 0.726 |
| HLA-DRA | PSMB9 | CD8T | 0.726 |
| HLA-DRA | C1QB | CD8T | 0.722 |
| HLA-DRA | CD4 | CD8T | 0.722 |
| HLA-DRA | LILRB4 | CD8T | 0.72 |
| HLA-DRA | IGSF6 | CD8T | 0.72 |
| HLA-DRA | ARRB2 | CD8T | 0.719 |
| HLA-DRA | LCP2 | CD8T | 0.715 |
| HLA-DRA | CASP1 | CD8T | 0.713 |
| HLA-DRA | C1QC | CD8T | 0.712 |
| HLA-DRA | CCL3 | CD8T | 0.71 |
| HLA-DRA | CYTH4 | CD8T | 0.71 |
| HLA-DRA | EVI2A | CD8T | 0.707 |
| HLA-DRA | ABI3 | CD8T | 0.705 |
| HLA-DRA | TNFSF13B | CD8T | 0.705 |
| HLA-DRA | LYL1 | CD8T | 0.705 |
| HLA-DRA | CTSZ | CD8T | 0.703 |
| HLA-DRA | SLC15A3 | CD8T | 0.703 |
| HLA-DRA | CD68 | CD8T | 0.701 |
| HLA-DRA | CD14 | CD8T | 0.7 |
| HLA-DRA | CCL4 | CD8T | 0.699 |
| HLA-DRA | FCGR3A | CD8T | 0.698 |
| HLA-DRA | FCGR2A | CD8T | 0.698 |
| HLA-DRA | ALOX5AP | CD8T | 0.697 |
| HLA-DRA | GNAI2 | CD8T | 0.693 |
| HLA-DRA | CCR1 | CD8T | 0.693 |
| HLA-DRA | CORO1A | CD8T | 0.692 |
| HLA-DRA | RGS19 | CD8T | 0.692 |
| HLA-DRA | CXCR4 | CD8T | 0.691 |
| HLA-DRA | FCGR1A | CD8T | 0.69 |
| HLA-DRA | RASSF4 | CD8T | 0.688 |
| HLA-DRA | CSF1R | CD8T | 0.686 |
| HLA-DRA | SNX10 | CD8T | 0.686 |
| HLA-DRA | TPP1 | CD8T | 0.684 |
| HLA-DRA | CD300A | CD8T | 0.681 |
| HLA-DRA | MPEG1 | CD8T | 0.681 |
| HLA-DRA | FPR3 | CD8T | 0.68 |
| HLA-DRA | ACP5 | CD8T | 0.68 |
| HLA-DRA | DOK2 | CD8T | 0.68 |
| HLA-DRA | C3AR1 | CD8T | 0.678 |
| HLA-DRA | AP1S2 | CD8T | 0.678 |
| HLA-DRA | SAMHD1 | CD8T | 0.678 |
| HLA-DRA | CCL4L2 | CD8T | 0.678 |
| HLA-DRA | ITGAX | CD8T | 0.676 |
| HLA-DRA | LY96 | CD8T | 0.676 |
| HLA-DRA | IL18 | CD8T | 0.675 |
| HLA-DRA | GIMAP4 | CD8T | 0.675 |
| HLA-DRA | NFKBID | CD8T | 0.674 |
| HLA-DRA | OLR1 | CD8T | 0.672 |
| HLA-DRA | BCL2A1 | CD8T | 0.67 |
| HLA-DRA | MPP1 | CD8T | 0.669 |
| HLA-DRA | TNFAIP8L2 | CD8T | 0.667 |
| HLA-DRA | CSF2RA | CD8T | 0.667 |
| HLA-DRA | SELPLG | CD8T | 0.662 |
| HLA-DRA | LRRC25 | CD8T | 0.662 |
| HLA-DRA | CLEC4A | CD8T | 0.66 |
| HLA-DRA | CYBA | CD8T | 0.659 |
| HLA-DRA | FAM49B | CD8T | 0.659 |
| HLA-DRA | GBP4 | CD8T | 0.657 |
| HLA-DRA | SAMD9L | CD8T | 0.657 |
| HLA-DRA | IL2RG | CD8T | 0.655 |
| HLA-DRA | RHOG | CD8T | 0.655 |
| HLA-DRA | KCTD12 | CD8T | 0.655 |
| HLA-DRA | FGR | CD8T | 0.653 |
| HLA-DRA | LILRB2 | CD8T | 0.653 |
| HLA-DRA | SIGLEC10 | CD8T | 0.652 |
| HLA-DRA | NCF2 | CD8T | 0.652 |
| HLA-DRA | APOC1 | CD8T | 0.651 |
| HLA-DRA | IFI30 | CD8T | 0.65 |
| HLA-DRA | UNC93B1 | CD8T | 0.65 |
| HLA-DRA | CSK | CD8T | 0.649 |
| HLA-DRA | IRF8 | CD8T | 0.648 |
| HLA-DRA | TREM2 | CD8T | 0.648 |
| HLA-DRA | FGL2 | CD8T | 0.646 |
| HLA-DRA | PARP14 | CD8T | 0.646 |
| HLA-DRA | SLAMF8 | CD8T | 0.644 |
| HLA-DRA | STAT1 | CD8T | 0.644 |
| HLA-DRA | CNPY3 | CD8T | 0.641 |
| HLA-DRA | SLA | CD8T | 0.64 |
| HLA-DRA | MS4A4A | CD8T | 0.639 |
| HLA-DRA | CXorf21 | CD8T | 0.638 |
| HLA-DRA | CD52 | CD8T | 0.637 |
| HLA-DRA | IL4I1 | CD8T | 0.637 |
| HLA-DRA | SERPINB9 | CD8T | 0.633 |
| HLA-DRA | VSIG4 | CD8T | 0.632 |
| HLA-DRA | SP110 | CD8T | 0.632 |
| HLA-DRA | EFHD2 | CD8T | 0.632 |
| HLA-DRA | LIMD2 | CD8T | 0.631 |
| HLA-DRA | RNASE1 | CD8T | 0.629 |
| HLA-DRA | TGFB1 | CD8T | 0.629 |
| HLA-DRA | CYTIP | CD8T | 0.626 |
| HLA-DRA | CD163 | CD8T | 0.625 |
| HLA-DRA | ADAM8 | CD8T | 0.625 |
| HLA-DRA | GRN | CD8T | 0.624 |
| HLA-DRA | SH2B3 | CD8T | 0.623 |
| HLA-DRA | FCGR1B | CD8T | 0.622 |
| HLA-DRA | TCIRG1 | CD8T | 0.62 |
| HLA-DRA | CTSD | CD8T | 0.617 |
| HLA-DRA | SPP1 | CD8T | 0.617 |
| HLA-DRA | CALHM6 | CD8T | 0.616 |
| HLA-DRA | APOBR | CD8T | 0.616 |
| HLA-DRA | TNFRSF14 | CD8T | 0.615 |
| HLA-DRA | OSCAR | CD8T | 0.614 |
| HLA-DRA | IFI44L | CD8T | 0.614 |
| HLA-DRA | IGKC | CD8T | 0.614 |
| HLA-DRA | LAP3 | CD8T | 0.613 |
| HLA-DRA | MYO1G | CD8T | 0.613 |
| HLA-DRA | CCRL2 | CD8T | 0.612 |
| HLA-DRA | C5AR1 | CD8T | 0.612 |
| HLA-DRA | STK4 | CD8T | 0.611 |
| HLA-DRA | ARHGAP9 | CD8T | 0.61 |
| HLA-DRA | XAF1 | CD8T | 0.608 |
| HLA-DRA | SGK1 | CD8T | 0.605 |
| HLA-DRA | LSP1 | CD8T | 0.603 |
| HLA-DRA | CSF2RB | CD8T | 0.601 |
| HLA-DRA | HHEX | CD8T | 0.6 |
| HLA-DRA | SIGLEC1 | CD8T | 0.597 |
| HLA-DRA | MOB1A | CD8T | 0.597 |
| HLA-DRA | ACTR2 | CD8T | 0.597 |
| HLA-DRA | GPR34 | CD8T | 0.596 |
| HLA-DRA | CLEC7A | CD8T | 0.596 |
| HLA-DRA | SAMSN1 | CD8T | 0.594 |
| HLA-DRA | TLR7 | CD8T | 0.592 |
| HLA-DRA | SLC16A3 | CD8T | 0.59 |
| HLA-DRA | IL1B | CD8T | 0.59 |
| HLA-DRA | PPP1R18 | CD8T | 0.589 |
| HLA-DRA | CXCL10 | CD8T | 0.587 |
| HLA-DRA | VSIR | CD8T | 0.587 |
| HLA-DRA | LILRB3 | CD8T | 0.586 |
| HLA-DRA | SLAMF7 | CD8T | 0.585 |
| HLA-DRA | CCL3L1 | CD8T | 0.583 |
| HLA-DRA | OTUD1 | CD8T | 0.583 |
| HLA-DRA | ADGRE5 | CD8T | 0.582 |
| HLA-DRA | PLA2G7 | CD8T | 0.582 |
| HLA-DRA | GBP5 | CD8T | 0.581 |
| HLA-DRA | CD40 | CD8T | 0.581 |
| HLA-DRA | TNFRSF1B | CD8T | 0.58 |
| HLA-DRA | GPR84 | CD8T | 0.579 |
| HLA-DRA | PTGER4 | CD8T | 0.578 |
| HLA-DRA | IL10 | CD8T | 0.576 |
| HLA-DRA | SMIM25 | CD8T | 0.576 |
| HLA-DRA | CLEC4E | CD8T | 0.575 |
| HLA-DRA | PYCARD | CD8T | 0.574 |
| HLA-DRA | B2M | CD8T | 0.574 |
| HLA-DRA | IFI35 | CD8T | 0.572 |
| HLA-DRA | APOBEC3C | CD8T | 0.572 |
| HLA-DRA | set/06 | CD8T | 0.571 |
| HLA-DRA | RAC2 | CD8T | 0.57 |
| HLA-DRA | CTSC | CD8T | 0.57 |
| HLA-DRA | NR4A2 | CD8T | 0.567 |
| HLA-DRA | REL | CD8T | 0.567 |
| HLA-DRA | RNF213 | CD8T | 0.566 |
| HLA-DRA | OSM | CD8T | 0.565 |
| HLA-DRA | IL18BP | CD8T | 0.564 |
| HLA-DRA | STAB1 | CD8T | 0.563 |
| HLA-DRA | P2RY13 | CD8T | 0.563 |
| HLA-DRA | CCR5 | CD8T | 0.563 |
| HLA-DRA | GIMAP1 | CD8T | 0.562 |
| HLA-DRA | IFIT3 | CD8T | 0.56 |
| HLA-DRA | ADORA3 | CD8T | 0.56 |
| HLA-DRA | JAML | CD8T | 0.558 |
| HLA-DRA | RGS2 | CD8T | 0.558 |
| HLA-DRA | STK17B | CD8T | 0.558 |
| HLA-DRA | CAPG | CD8T | 0.557 |
| HLA-DRA | SLC37A2 | CD8T | 0.557 |
| HLA-DRA | RENBP | CD8T | 0.555 |
| HLA-DRA | MRC1 | CD8T | 0.554 |
| HLA-DRA | OAS1 | CD8T | 0.553 |
| HLA-DRA | VMO1 | CD8T | 0.552 |
| HLA-DRA | TMEM273 | CD8T | 0.551 |
| HLA-DRA | LINC01857 | CD8T | 0.55 |
| HLA-DRA | SUCNR1 | CD8T | 0.549 |
| HLA-DRA | GAPT | CD8T | 0.549 |
| HLA-DRA | NPC2 | CD8T | 0.548 |
| HLA-DRA | ADAMDEC1 | CD8T | 0.546 |
| HLA-DRA | SLC11A1 | CD8T | 0.545 |
| HLA-DRA | LGMN | CD8T | 0.541 |
| HLA-DRA | RILPL2 | CD8T | 0.541 |
| HLA-DRA | POU2F2 | CD8T | 0.539 |
| HLA-DRA | GBP1 | CD8T | 0.538 |
| HLA-DRA | MX1 | CD8T | 0.535 |
| HLA-DRA | LMO2 | CD8T | 0.535 |
| HLA-DRA | SPN | CD8T | 0.535 |
| HLA-DRA | GAL3ST4 | CD8T | 0.534 |
| HLA-DRA | AKR1B1 | CD8T | 0.533 |
| HLA-DRA | IGLC2 | CD8T | 0.533 |
| HLA-DRA | TXNIP | CD8T | 0.533 |
| HLA-DRA | CD69 | CD8T | 0.533 |
| HLA-DRA | NLRP3 | CD8T | 0.532 |
| HLA-DRA | CXCL9 | CD8T | 0.53 |
| HLA-DRA | CTSB | CD8T | 0.53 |
| HLA-DRA | CLEC2B | CD8T | 0.53 |
| HLA-DRA | GLRX | CD8T | 0.528 |
| HLA-DRA | RNASE2 | CD8T | 0.528 |
| HLA-DRA | TGFBI | CD8T | 0.528 |
| HLA-DRA | ARPC1B | CD8T | 0.527 |
| HLA-DRA | HAMP | CD8T | 0.526 |
| HLA-DRA | PARP9 | CD8T | 0.525 |
| HLA-DRA | C2 | CD8T | 0.525 |
| HLA-DRA | IFIT2 | CD8T | 0.524 |
| HLA-DRA | IGLC3 | CD8T | 0.524 |
| HLA-DRA | SDS | CD8T | 0.523 |
| HLA-DRA | CSF3R | CD8T | 0.521 |
| HLA-DRA | UCP2 | CD8T | 0.52 |
| HLA-DRA | APOE | CD8T | 0.52 |
| HLA-DRA | MIR155HG | CD8T | 0.52 |
| HLA-DRA | PPT1 | CD8T | 0.52 |
| HLA-DRA | COTL1 | CD8T | 0.519 |
| HLA-DRA | MMP9 | CD8T | 0.519 |
| HLA-DRA | ANKRD22 | CD8T | 0.518 |
| HLA-DRA | NR1H3 | CD8T | 0.517 |

**Table S6 –** List of genes significantly correlated with HLA-DR from BRCA_EMTAB8107 database

| Gene | Gene 2 | Cell Type | Correlation value |
| --- | --- | --- | --- |
| HLA-DRA | CD74 | CD8T | 0.794 |
| HLA-DRA | LAG3 | CD8T | 0.763 |
| HLA-DRA | FASLG | CD8T | 0.742 |
| HLA-DRA | IFNG | CD8T | 0.727 |
| HLA-DRA | PTMS | CD8T | 0.717 |
| HLA-DRA | ALOX5AP | CD8T | 0.701 |
| HLA-DRA | APOBEC3C | CD8T | 0.695 |
| HLA-DRA | CCL3 | CD8T | 0.69 |
| HLA-DRA | GZMH | CD8T | 0.684 |
| HLA-DRA | FABP5 | CD8T | 0.679 |
| HLA-DRA | OASL | CD8T | 0.678 |
| HLA-DRA | AC069363.1 | CD8T | 0.677 |
| HLA-DRA | GZMB | CD8T | 0.673 |
| HLA-DRA | CXCR6 | CD8T | 0.665 |
| HLA-DRA | CTSC | CD8T | 0.661 |
| HLA-DRA | IDH2 | CD8T | 0.647 |
| HLA-DRA | MT2A | CD8T | 0.644 |
| HLA-DRA | GBP5 | CD8T | 0.643 |
| HLA-DRA | ANXA5 | CD8T | 0.643 |
| HLA-DRA | COTL1 | CD8T | 0.636 |
| HLA-DRA | PDCD1 | CD8T | 0.631 |
| HLA-DRA | PRF1 | CD8T | 0.629 |
| HLA-DRA | CCL4 | CD8T | 0.624 |
| HLA-DRA | PSMB9 | CD8T | 0.622 |
| HLA-DRA | APOBEC3G | CD8T | 0.622 |
| HLA-DRA | ARPC2 | CD8T | 0.618 |
| HLA-DRA | MT1E | CD8T | 0.613 |
| HLA-DRA | ID2 | CD8T | 0.608 |
| HLA-DRA | TAP1 | CD8T | 0.607 |
| HLA-DRA | MT1F | CD8T | 0.605 |
| HLA-DRA | JAML | CD8T | 0.604 |
| HLA-DRA | GAPDH | CD8T | 0.603 |
| HLA-DRA | TIGIT | CD8T | 0.603 |
| HLA-DRA | MIR155HG | CD8T | 0.598 |
| HLA-DRA | VCAM1 | CD8T | 0.595 |
| HLA-DRA | CD2 | CD8T | 0.591 |
| HLA-DRA | LGALS1 | CD8T | 0.59 |
| HLA-DRA | PTPN7 | CD8T | 0.586 |
| HLA-DRA | GZMA | CD8T | 0.583 |
| HLA-DRA | KRT86 | CD8T | 0.58 |
| HLA-DRA | PLPP1 | CD8T | 0.578 |
| HLA-DRA | IFI16 | CD8T | 0.578 |
| HLA-DRA | LY6E | CD8T | 0.575 |
| HLA-DRA | IFI35 | CD8T | 0.572 |
| HLA-DRA | CCND2 | CD8T | 0.571 |
| HLA-DRA | PLSCR1 | CD8T | 0.567 |
| HLA-DRA | CCR5 | CD8T | 0.566 |
| HLA-DRA | ACP5 | CD8T | 0.565 |
| HLA-DRA | SUB1 | CD8T | 0.562 |
| HLA-DRA | CDKN2A | CD8T | 0.558 |
| HLA-DRA | IFI27L2 | CD8T | 0.556 |
| HLA-DRA | DUSP4 | CD8T | 0.555 |
| HLA-DRA | ABI3 | CD8T | 0.555 |
| HLA-DRA | SAMD9L | CD8T | 0.555 |
| HLA-DRA | CTSD | CD8T | 0.551 |
| HLA-DRA | TNFRSF9 | CD8T | 0.549 |
| HLA-DRA | RBCK1 | CD8T | 0.547 |
| HLA-DRA | CXCL13 | CD8T | 0.544 |
| HLA-DRA | ISG15 | CD8T | 0.543 |
| HLA-DRA | PLEKHF1 | CD8T | 0.542 |
| HLA-DRA | ADGRE5 | CD8T | 0.542 |
| HLA-DRA | ACTN4 | CD8T | 0.541 |
| HLA-DRA | IFITM3 | CD8T | 0.54 |
| HLA-DRA | CCL4L2 | CD8T | 0.537 |
| HLA-DRA | PTTG1 | CD8T | 0.537 |
| HLA-DRA | CAPZB | CD8T | 0.536 |
| HLA-DRA | UBE2L6 | CD8T | 0.536 |
| HLA-DRA | POMP | CD8T | 0.535 |
| HLA-DRA | set/07 | CD8T | 0.53 |
| HLA-DRA | RHOA | CD8T | 0.528 |
| HLA-DRA | LSP1 | CD8T | 0.528 |
| HLA-DRA | TYMP | CD8T | 0.526 |
| HLA-DRA | ITM2A | CD8T | 0.526 |
| HLA-DRA | MTHFD2 | CD8T | 0.525 |
| HLA-DRA | CST7 | CD8T | 0.523 |
| HLA-DRA | GBP1 | CD8T | 0.521 |
| HLA-DRA | ANXA2 | CD8T | 0.521 |
| HLA-DRA | BST2 | CD8T | 0.521 |
| HLA-DRA | PARK7 | CD8T | 0.52 |
| HLA-DRA | LCP2 | CD8T | 0.52 |
| HLA-DRA | CAPZA1 | CD8T | 0.519 |
| HLA-DRA | SRGN | CD8T | 0.519 |
| HLA-DRA | CSF1 | CD8T | 0.519 |
| HLA-DRA | CAPN2 | CD8T | 0.517 |
| HLA-DRA | SHISA5 | CD8T | 0.515 |
| HLA-DRA | DRAP1 | CD8T | 0.515 |
| HLA-DRA | CLEC2D | CD8T | 0.514 |
| HLA-DRA | PDIA6 | CD8T | 0.513 |
| HLA-DRA | LGALS3 | CD8T | 0.512 |
| HLA-DRA | ACTB | CD8T | 0.511 |
| HLA-DRA | DYNLL1 | CD8T | 0.51 |

**Table S7 –** List of genes significantly correlated with PD1 from BRCA_GSE110686 database

| Gene | Gene 2 | Cell Type | Correlation value |
| --- | --- | --- | --- |
| PDCD1 | TIGIT | CD8T | 0.889 |
| PDCD1 | PHLDA1 | CD8T | 0.881 |
| PDCD1 | CCL3 | CD8T | 0.879 |
| PDCD1 | TNFRSF9 | CD8T | 0.865 |
| PDCD1 | FABP5 | CD8T | 0.854 |
| PDCD1 | GAPDH | CD8T | 0.845 |
| PDCD1 | CXCL13 | CD8T | 0.843 |
| PDCD1 | VCAM1 | CD8T | 0.838 |
| PDCD1 | HAVCR2 | CD8T | 0.834 |
| PDCD1 | BHLHE40 | CD8T | 0.83 |
| PDCD1 | GZMB | CD8T | 0.822 |
| PDCD1 | DUSP4 | CD8T | 0.821 |
| PDCD1 | IFNG | CD8T | 0.82 |
| PDCD1 | BATF | CD8T | 0.815 |
| PDCD1 | RGS2 | CD8T | 0.813 |
| PDCD1 | SYNGR2 | CD8T | 0.812 |
| PDCD1 | YWHAH | CD8T | 0.809 |
| PDCD1 | PKM | CD8T | 0.806 |
| PDCD1 | PTMS | CD8T | 0.805 |
| PDCD1 | LAG3 | CD8T | 0.805 |
| PDCD1 | MIR155HG | CD8T | 0.799 |
| PDCD1 | ANXA5 | CD8T | 0.798 |
| PDCD1 | SH2D2A | CD8T | 0.797 |
| PDCD1 | AC069363.1 | CD8T | 0.797 |
| PDCD1 | ACP5 | CD8T | 0.795 |
| PDCD1 | SIRPG | CD8T | 0.793 |
| PDCD1 | FASLG | CD8T | 0.793 |
| PDCD1 | ID2 | CD8T | 0.792 |
| PDCD1 | FKBP1A | CD8T | 0.791 |
| PDCD1 | CXCR6 | CD8T | 0.787 |
| PDCD1 | AC133644.2 | CD8T | 0.786 |
| PDCD1 | CCND2 | CD8T | 0.776 |
| PDCD1 | RHOA | CD8T | 0.775 |
| PDCD1 | LSP1 | CD8T | 0.773 |
| PDCD1 | CD27 | CD8T | 0.771 |
| PDCD1 | ITM2A | CD8T | 0.767 |
| PDCD1 | SIT1 | CD8T | 0.766 |
| PDCD1 | CD82 | CD8T | 0.764 |
| PDCD1 | PARK7 | CD8T | 0.761 |
| PDCD1 | ID3 | CD8T | 0.76 |
| PDCD1 | TNFRSF1B | CD8T | 0.75 |
| PDCD1 | CCL4 | CD8T | 0.748 |
| PDCD1 | HSPB1 | CD8T | 0.747 |
| PDCD1 | DYNLL1 | CD8T | 0.746 |
| PDCD1 | CD74 | CD8T | 0.738 |
| PDCD1 | SCAMP2 | CD8T | 0.735 |
| PDCD1 | PGAM1 | CD8T | 0.733 |
| PDCD1 | CKS2 | CD8T | 0.73 |
| PDCD1 | HSBP1 | CD8T | 0.727 |
| PDCD1 | CTLA4 | CD8T | 0.726 |
| PDCD1 | DNPH1 | CD8T | 0.723 |
| PDCD1 | PRDX6 | CD8T | 0.721 |
| PDCD1 | COPZ1 | CD8T | 0.721 |
| PDCD1 | LITAF | CD8T | 0.72 |
| PDCD1 | NR4A2 | CD8T | 0.717 |
| PDCD1 | PRF1 | CD8T | 0.71 |
| PDCD1 | KLRC1 | CD8T | 0.709 |
| PDCD1 | GNLY | CD8T | 0.708 |
| PDCD1 | SRI | CD8T | 0.706 |
| PDCD1 | ALOX5AP | CD8T | 0.706 |
| PDCD1 | SOX4 | CD8T | 0.705 |
| PDCD1 | SNRPD1 | CD8T | 0.704 |
| PDCD1 | ZBED2 | CD8T | 0.703 |
| PDCD1 | NDUFA13 | CD8T | 0.698 |
| PDCD1 | SLA | CD8T | 0.696 |
| PDCD1 | SUMO2 | CD8T | 0.696 |
| PDCD1 | CD63 | CD8T | 0.694 |
| PDCD1 | CSF1 | CD8T | 0.693 |
| PDCD1 | DBI | CD8T | 0.689 |
| PDCD1 | TRAPPC1 | CD8T | 0.688 |
| PDCD1 | CD2 | CD8T | 0.685 |
| PDCD1 | LAYN | CD8T | 0.684 |
| PDCD1 | GALM | CD8T | 0.684 |
| PDCD1 | SAMSN1 | CD8T | 0.681 |
| PDCD1 | RABAC1 | CD8T | 0.68 |
| PDCD1 | GZMH | CD8T | 0.68 |
| PDCD1 | PTTG1 | CD8T | 0.679 |
| PDCD1 | SOD1 | CD8T | 0.677 |
| PDCD1 | VCP | CD8T | 0.676 |
| PDCD1 | COX8A | CD8T | 0.673 |
| PDCD1 | TRAC | CD8T | 0.673 |
| PDCD1 | CDK2AP2 | CD8T | 0.671 |
| PDCD1 | IFI27L2 | CD8T | 0.671 |
| PDCD1 | CALM3 | CD8T | 0.669 |
| PDCD1 | EZR | CD8T | 0.667 |
| PDCD1 | PRDM1 | CD8T | 0.667 |
| PDCD1 | CHST12 | CD8T | 0.667 |
| PDCD1 | CLEC2D | CD8T | 0.66 |
| PDCD1 | PTPN6 | CD8T | 0.659 |
| PDCD1 | IGFLR1 | CD8T | 0.658 |
| PDCD1 | CCR5 | CD8T | 0.655 |
| PDCD1 | APOBEC3C | CD8T | 0.655 |
| PDCD1 | SUB1 | CD8T | 0.653 |
| PDCD1 | SRGN | CD8T | 0.651 |
| PDCD1 | PSMB3 | CD8T | 0.65 |
| PDCD1 | CD8A | CD8T | 0.649 |
| PDCD1 | CD79A | CD8T | 0.648 |
| PDCD1 | CARHSP1 | CD8T | 0.647 |
| PDCD1 | CLEC2B | CD8T | 0.646 |
| PDCD1 | SDC4 | CD8T | 0.645 |
| PDCD1 | MYL6 | CD8T | 0.643 |
| PDCD1 | CAP1 | CD8T | 0.642 |
| PDCD1 | ADGRE5 | CD8T | 0.642 |
| PDCD1 | PDIA6 | CD8T | 0.641 |
| PDCD1 | TPI1 | CD8T | 0.638 |
| PDCD1 | IL2RG | CD8T | 0.637 |
| PDCD1 | TSPO | CD8T | 0.635 |
| PDCD1 | TXNDC17 | CD8T | 0.632 |
| PDCD1 | HNRNPK | CD8T | 0.631 |
| PDCD1 | CYCS | CD8T | 0.63 |
| PDCD1 | MCM5 | CD8T | 0.628 |
| PDCD1 | CD2BP2 | CD8T | 0.628 |
| PDCD1 | PRDX5 | CD8T | 0.628 |
| PDCD1 | SERPINB9 | CD8T | 0.627 |
| PDCD1 | ARPP19 | CD8T | 0.627 |
| PDCD1 | KLRC2 | CD8T | 0.627 |
| PDCD1 | NRBP1 | CD8T | 0.626 |
| PDCD1 | SRP14 | CD8T | 0.626 |
| PDCD1 | UCP2 | CD8T | 0.623 |
| PDCD1 | IFI27 | CD8T | 0.622 |
| PDCD1 | CLIC1 | CD8T | 0.622 |
| PDCD1 | EID1 | CD8T | 0.621 |
| PDCD1 | MCL1 | CD8T | 0.62 |
| PDCD1 | MT1E | CD8T | 0.619 |
| PDCD1 | HERPUD1 | CD8T | 0.618 |
| PDCD1 | CCR1 | CD8T | 0.618 |
| PDCD1 | PRDX1 | CD8T | 0.617 |
| PDCD1 | TRBC1 | CD8T | 0.617 |
| PDCD1 | HNRNPLL | CD8T | 0.616 |
| PDCD1 | UBB | CD8T | 0.616 |
| PDCD1 | SLC1A5 | CD8T | 0.615 |
| PDCD1 | TMED9 | CD8T | 0.615 |
| PDCD1 | ARPC2 | CD8T | 0.615 |
| PDCD1 | NKG7 | CD8T | 0.613 |
| PDCD1 | RGS1 | CD8T | 0.613 |
| PDCD1 | PHPT1 | CD8T | 0.613 |
| PDCD1 | SNU13 | CD8T | 0.612 |
| PDCD1 | APOBEC3G | CD8T | 0.61 |
| PDCD1 | NDUFB3 | CD8T | 0.609 |
| PDCD1 | NUSAP1 | CD8T | 0.607 |
| PDCD1 | COTL1 | CD8T | 0.607 |
| PDCD1 | LGALS1 | CD8T | 0.606 |
| PDCD1 | LINC01480 | CD8T | 0.606 |
| PDCD1 | NDUFC1 | CD8T | 0.605 |
| PDCD1 | KRT86 | CD8T | 0.604 |
| PDCD1 | SURF4 | CD8T | 0.601 |
| PDCD1 | FDPS | CD8T | 0.6 |
| PDCD1 | PMF1 | CD8T | 0.6 |
| PDCD1 | CTSC | CD8T | 0.599 |
| PDCD1 | LAMTOR5 | CD8T | 0.599 |
| PDCD1 | NUDT5 | CD8T | 0.598 |
| PDCD1 | KIF20B | CD8T | 0.598 |
| PDCD1 | HMGN3 | CD8T | 0.597 |
| PDCD1 | CD3D | CD8T | 0.596 |
| PDCD1 | GZMA | CD8T | 0.595 |
| PDCD1 | BUB3 | CD8T | 0.593 |
| PDCD1 | HNRNPA3 | CD8T | 0.591 |
| PDCD1 | DCTN3 | CD8T | 0.59 |
| PDCD1 | MOB1A | CD8T | 0.589 |
| PDCD1 | SLC25A3 | CD8T | 0.588 |
| PDCD1 | SLC25A11 | CD8T | 0.588 |
| PDCD1 | HMGN1 | CD8T | 0.588 |
| PDCD1 | ITGB1 | CD8T | 0.586 |
| PDCD1 | DEF6 | CD8T | 0.585 |
| PDCD1 | RHOC | CD8T | 0.584 |
| PDCD1 | CCDC28B | CD8T | 0.584 |
| PDCD1 | COX5A | CD8T | 0.583 |
| PDCD1 | U2AF1L4 | CD8T | 0.583 |
| PDCD1 | MRPS34 | CD8T | 0.581 |
| PDCD1 | MT1X | CD8T | 0.58 |
| PDCD1 | IVNS1ABP | CD8T | 0.579 |
| PDCD1 | GTF3C6 | CD8T | 0.578 |
| PDCD1 | PSMC4 | CD8T | 0.575 |
| PDCD1 | ANP32A | CD8T | 0.575 |
| PDCD1 | SH2D1A | CD8T | 0.575 |
| PDCD1 | SDF2L1 | CD8T | 0.574 |
| PDCD1 | SKA2 | CD8T | 0.574 |
| PDCD1 | CCNDBP1 | CD8T | 0.573 |
| PDCD1 | AIF1 | CD8T | 0.57 |
| PDCD1 | DNAJC8 | CD8T | 0.569 |
| PDCD1 | COMMD7 | CD8T | 0.569 |
| PDCD1 | PSMC3 | CD8T | 0.568 |
| PDCD1 | ABI3 | CD8T | 0.568 |
| PDCD1 | CFL1 | CD8T | 0.567 |
| PDCD1 | CYB5B | CD8T | 0.567 |
| PDCD1 | PFDN2 | CD8T | 0.567 |
| PDCD1 | CARD16 | CD8T | 0.567 |
| PDCD1 | CAPZB | CD8T | 0.566 |
| PDCD1 | SAP18 | CD8T | 0.566 |
| PDCD1 | AP2S1 | CD8T | 0.565 |
| PDCD1 | CST7 | CD8T | 0.565 |
| PDCD1 | SERF2 | CD8T | 0.564 |
| PDCD1 | GTF2A2 | CD8T | 0.564 |
| PDCD1 | HSD17B10 | CD8T | 0.564 |
| PDCD1 | ARL6IP1 | CD8T | 0.562 |
| PDCD1 | COX6C | CD8T | 0.562 |
| PDCD1 | B3GNT2 | CD8T | 0.561 |
| PDCD1 | MSN | CD8T | 0.559 |
| PDCD1 | ARHGEF1 | CD8T | 0.559 |
| PDCD1 | TMCO1 | CD8T | 0.558 |
| PDCD1 | MRPS11 | CD8T | 0.558 |
| PDCD1 | EIF4E2 | CD8T | 0.558 |
| PDCD1 | COX7A2 | CD8T | 0.557 |
| PDCD1 | TRAPPC4 | CD8T | 0.557 |
| PDCD1 | TXN | CD8T | 0.556 |
| PDCD1 | EIF4H | CD8T | 0.554 |
| PDCD1 | ENSA | CD8T | 0.554 |
| PDCD1 | TMEM179B | CD8T | 0.553 |
| PDCD1 | MRPL51 | CD8T | 0.552 |
| PDCD1 | ITGB7 | CD8T | 0.552 |
| PDCD1 | PSMA7 | CD8T | 0.552 |
| PDCD1 | NDUFS6 | CD8T | 0.552 |
| PDCD1 | UBE2A | CD8T | 0.551 |
| PDCD1 | KPNA2 | CD8T | 0.55 |
| PDCD1 | YIF1A | CD8T | 0.55 |
| PDCD1 | PTPN7 | CD8T | 0.548 |
| PDCD1 | CD8B | CD8T | 0.547 |
| PDCD1 | RBX1 | CD8T | 0.547 |
| PDCD1 | MLF2 | CD8T | 0.547 |
| PDCD1 | KXD1 | CD8T | 0.546 |
| PDCD1 | GSTO1 | CD8T | 0.545 |
| PDCD1 | HSPA5 | CD8T | 0.544 |
| PDCD1 | ROMO1 | CD8T | 0.544 |
| PDCD1 | UQCR10 | CD8T | 0.544 |
| PDCD1 | PYCARD | CD8T | 0.543 |
| PDCD1 | OAZ1 | CD8T | 0.542 |
| PDCD1 | CMTM6 | CD8T | 0.541 |
| PDCD1 | RNF181 | CD8T | 0.541 |
| PDCD1 | DDA1 | CD8T | 0.539 |
| PDCD1 | LSM8 | CD8T | 0.537 |
| PDCD1 | RHBDD2 | CD8T | 0.537 |
| PDCD1 | LCK | CD8T | 0.536 |
| PDCD1 | PPP4C | CD8T | 0.535 |
| PDCD1 | PSME2 | CD8T | 0.535 |
| PDCD1 | H2AFZ | CD8T | 0.534 |
| PDCD1 | MAP2K3 | CD8T | 0.534 |
| PDCD1 | TALDO1 | CD8T | 0.534 |
| PDCD1 | GGCT | CD8T | 0.534 |
| PDCD1 | LBH | CD8T | 0.534 |
| PDCD1 | ARPC1B | CD8T | 0.534 |
| PDCD1 | CAPZA1 | CD8T | 0.534 |
| PDCD1 | EDF1 | CD8T | 0.533 |
| PDCD1 | SMC4 | CD8T | 0.532 |
| PDCD1 | SLC16A3 | CD8T | 0.532 |
| PDCD1 | DNAJB11 | CD8T | 0.53 |
| PDCD1 | YWHAQ | CD8T | 0.53 |
| PDCD1 | IDH2 | CD8T | 0.53 |
| PDCD1 | BAX | CD8T | 0.53 |
| PDCD1 | HSP90B1 | CD8T | 0.53 |
| PDCD1 | COX6A1 | CD8T | 0.529 |
| PDCD1 | PSMA2 | CD8T | 0.527 |
| PDCD1 | KCNN4 | CD8T | 0.527 |
| PDCD1 | SEC11A | CD8T | 0.526 |
| PDCD1 | PSMA1 | CD8T | 0.526 |
| PDCD1 | TANK | CD8T | 0.526 |
| PDCD1 | RPS26 | CD8T | 0.525 |
| PDCD1 | AP2M1 | CD8T | 0.525 |
| PDCD1 | GTF2H5 | CD8T | 0.525 |
| PDCD1 | PEF1 | CD8T | 0.525 |
| PDCD1 | set/07 | CD8T | 0.524 |
| PDCD1 | TMX1 | CD8T | 0.523 |
| PDCD1 | UQCRFS1 | CD8T | 0.522 |
| PDCD1 | MPC2 | CD8T | 0.52 |
| PDCD1 | SFT2D1 | CD8T | 0.519 |
| PDCD1 | GLOD4 | CD8T | 0.519 |
| PDCD1 | PDIA3 | CD8T | 0.518 |
| PDCD1 | SEC61G | CD8T | 0.518 |
| PDCD1 | CTSW | CD8T | 0.517 |
| PDCD1 | HNRNPC | CD8T | 0.516 |
| PDCD1 | LSM1 | CD8T | 0.516 |

**Table S8 –** List of genes significantly correlated with PD1 from BRCA_GSE114727_10X database

| Gene | Gene 2 | Cell Type | Correlation value |
| --- | --- | --- | --- |
| PDCD1 | MIR155HG | CD8T | 0.851 |
| PDCD1 | CXCL13 | CD8T | 0.835 |
| PDCD1 | CTLA4 | CD8T | 0.827 |
| PDCD1 | FABP5 | CD8T | 0.813 |
| PDCD1 | ITGAE | CD8T | 0.798 |
| PDCD1 | CCL3 | CD8T | 0.796 |
| PDCD1 | ANXA5 | CD8T | 0.795 |
| PDCD1 | AC069363.1 | CD8T | 0.788 |
| PDCD1 | ACP5 | CD8T | 0.764 |
| PDCD1 | SIRPG | CD8T | 0.763 |
| PDCD1 | JAML | CD8T | 0.762 |
| PDCD1 | PTTG1 | CD8T | 0.76 |
| PDCD1 | RGS1 | CD8T | 0.747 |
| PDCD1 | AC133644.2 | CD8T | 0.747 |
| PDCD1 | AKAP5 | CD8T | 0.742 |
| PDCD1 | CXCR6 | CD8T | 0.728 |
| PDCD1 | LAYN | CD8T | 0.719 |
| PDCD1 | IFNG | CD8T | 0.71 |
| PDCD1 | TNFRSF1B | CD8T | 0.709 |
| PDCD1 | PARK7 | CD8T | 0.709 |
| PDCD1 | CTSD | CD8T | 0.707 |
| PDCD1 | TIGIT | CD8T | 0.706 |
| PDCD1 | COMMD7 | CD8T | 0.703 |
| PDCD1 | LSP1 | CD8T | 0.703 |
| PDCD1 | IFI27L2 | CD8T | 0.698 |
| PDCD1 | PTMS | CD8T | 0.695 |
| PDCD1 | GZMB | CD8T | 0.692 |
| PDCD1 | CAPZB | CD8T | 0.689 |
| PDCD1 | TNFRSF9 | CD8T | 0.689 |
| PDCD1 | CARD16 | CD8T | 0.689 |
| PDCD1 | WDR1 | CD8T | 0.684 |
| PDCD1 | ALOX5AP | CD8T | 0.681 |
| PDCD1 | SUB1 | CD8T | 0.681 |
| PDCD1 | ITM2A | CD8T | 0.68 |
| PDCD1 | RALY | CD8T | 0.679 |
| PDCD1 | AC017002.1 | CD8T | 0.678 |
| PDCD1 | PPM1M | CD8T | 0.677 |
| PDCD1 | BATF | CD8T | 0.677 |
| PDCD1 | ITGB1 | CD8T | 0.677 |
| PDCD1 | CD200 | CD8T | 0.672 |
| PDCD1 | LAG3 | CD8T | 0.669 |
| PDCD1 | CLEC2D | CD8T | 0.666 |
| PDCD1 | BTG3 | CD8T | 0.665 |
| PDCD1 | TXNDC17 | CD8T | 0.661 |
| PDCD1 | SEC11A | CD8T | 0.66 |
| PDCD1 | PKM | CD8T | 0.66 |
| PDCD1 | SMC4 | CD8T | 0.66 |
| PDCD1 | CD63 | CD8T | 0.659 |
| PDCD1 | FKBP1A | CD8T | 0.658 |
| PDCD1 | EVL | CD8T | 0.658 |
| PDCD1 | IFI16 | CD8T | 0.658 |
| PDCD1 | APOBEC3C | CD8T | 0.657 |
| PDCD1 | HMGB1 | CD8T | 0.654 |
| PDCD1 | ENSA | CD8T | 0.653 |
| PDCD1 | KRT86 | CD8T | 0.653 |
| PDCD1 | BLOC1S1 | CD8T | 0.65 |
| PDCD1 | MSN | CD8T | 0.649 |
| PDCD1 | CTSC | CD8T | 0.647 |
| PDCD1 | PPM1G | CD8T | 0.647 |
| PDCD1 | PSMD8 | CD8T | 0.646 |
| PDCD1 | LCP2 | CD8T | 0.645 |
| PDCD1 | NR3C1 | CD8T | 0.644 |
| PDCD1 | GAPDH | CD8T | 0.643 |
| PDCD1 | RHOA | CD8T | 0.643 |
| PDCD1 | DUSP4 | CD8T | 0.639 |
| PDCD1 | PDIA6 | CD8T | 0.638 |
| PDCD1 | ARPC2 | CD8T | 0.633 |
| PDCD1 | CARHSP1 | CD8T | 0.633 |
| PDCD1 | APBB1IP | CD8T | 0.632 |
| PDCD1 | TMX4 | CD8T | 0.631 |
| PDCD1 | SLA | CD8T | 0.631 |
| PDCD1 | TMEM120A | CD8T | 0.628 |
| PDCD1 | CD2BP2 | CD8T | 0.627 |
| PDCD1 | POMP | CD8T | 0.626 |
| PDCD1 | COX5A | CD8T | 0.626 |
| PDCD1 | PYCARD | CD8T | 0.625 |
| PDCD1 | DYNLL1 | CD8T | 0.624 |
| PDCD1 | set/07 | CD8T | 0.623 |
| PDCD1 | FIBP | CD8T | 0.622 |
| PDCD1 | GZMH | CD8T | 0.622 |
| PDCD1 | GBP2 | CD8T | 0.62 |
| PDCD1 | LCK | CD8T | 0.618 |
| PDCD1 | MZB1 | CD8T | 0.617 |
| PDCD1 | GNAS | CD8T | 0.617 |
| PDCD1 | ARHGAP9 | CD8T | 0.615 |
| PDCD1 | CDCA7 | CD8T | 0.614 |
| PDCD1 | CSK | CD8T | 0.614 |
| PDCD1 | RAC1 | CD8T | 0.613 |
| PDCD1 | MAP4K1 | CD8T | 0.612 |
| PDCD1 | LINC01480 | CD8T | 0.611 |
| PDCD1 | CAP1 | CD8T | 0.609 |
| PDCD1 | PHYKPL | CD8T | 0.608 |
| PDCD1 | TLN1 | CD8T | 0.608 |
| PDCD1 | CD2 | CD8T | 0.608 |
| PDCD1 | MYL6B | CD8T | 0.608 |
| PDCD1 | CD3D | CD8T | 0.607 |
| PDCD1 | PPP1CA | CD8T | 0.607 |
| PDCD1 | GNAI2 | CD8T | 0.606 |
| PDCD1 | HSBP1 | CD8T | 0.606 |
| PDCD1 | CCND2 | CD8T | 0.605 |
| PDCD1 | EID1 | CD8T | 0.604 |
| PDCD1 | TADA3 | CD8T | 0.603 |
| PDCD1 | DEK | CD8T | 0.603 |
| PDCD1 | TWF2 | CD8T | 0.602 |
| PDCD1 | PSMB8 | CD8T | 0.6 |
| PDCD1 | CDK2AP2 | CD8T | 0.599 |
| PDCD1 | COPZ1 | CD8T | 0.598 |
| PDCD1 | COTL1 | CD8T | 0.597 |
| PDCD1 | PDZD11 | CD8T | 0.597 |
| PDCD1 | BUB3 | CD8T | 0.597 |
| PDCD1 | TSPO | CD8T | 0.596 |
| PDCD1 | PARP1 | CD8T | 0.595 |
| PDCD1 | PRF1 | CD8T | 0.593 |
| PDCD1 | CHST12 | CD8T | 0.593 |
| PDCD1 | PLEKHF1 | CD8T | 0.593 |
| PDCD1 | ACTR3 | CD8T | 0.592 |
| PDCD1 | SLC7A5 | CD8T | 0.592 |
| PDCD1 | GNG5 | CD8T | 0.591 |
| PDCD1 | AP2M1 | CD8T | 0.59 |
| PDCD1 | SH3BP1 | CD8T | 0.589 |
| PDCD1 | DYNLRB1 | CD8T | 0.589 |
| PDCD1 | ARF5 | CD8T | 0.588 |
| PDCD1 | UBE2L3 | CD8T | 0.586 |
| PDCD1 | CSF1 | CD8T | 0.585 |
| PDCD1 | CD3G | CD8T | 0.584 |
| PDCD1 | UCP2 | CD8T | 0.584 |
| PDCD1 | PAQR4 | CD8T | 0.581 |
| PDCD1 | UBE2A | CD8T | 0.58 |
| PDCD1 | RAB1B | CD8T | 0.579 |
| PDCD1 | HNRNPA3 | CD8T | 0.578 |
| PDCD1 | GEM | CD8T | 0.578 |
| PDCD1 | AKIRIN2 | CD8T | 0.578 |
| PDCD1 | PRDX3 | CD8T | 0.578 |
| PDCD1 | CD82 | CD8T | 0.577 |
| PDCD1 | ZCRB1 | CD8T | 0.576 |
| PDCD1 | LAMTOR5 | CD8T | 0.575 |
| PDCD1 | RNF187 | CD8T | 0.574 |
| PDCD1 | PGAM1 | CD8T | 0.574 |
| PDCD1 | WAS | CD8T | 0.574 |
| PDCD1 | IDH2 | CD8T | 0.574 |
| PDCD1 | MRPS36 | CD8T | 0.573 |
| PDCD1 | CMTM6 | CD8T | 0.573 |
| PDCD1 | ASNA1 | CD8T | 0.572 |
| PDCD1 | RABAC1 | CD8T | 0.569 |
| PDCD1 | TCIRG1 | CD8T | 0.569 |
| PDCD1 | PPP4C | CD8T | 0.569 |
| PDCD1 | APOBEC3G | CD8T | 0.569 |
| PDCD1 | EWSR1 | CD8T | 0.568 |
| PDCD1 | SYNGR2 | CD8T | 0.568 |
| PDCD1 | SERPINB1 | CD8T | 0.568 |
| PDCD1 | OTUB1 | CD8T | 0.567 |
| PDCD1 | TPM3 | CD8T | 0.567 |
| PDCD1 | PSMB2 | CD8T | 0.567 |
| PDCD1 | COPE | CD8T | 0.567 |
| PDCD1 | ACTB | CD8T | 0.565 |
| PDCD1 | ETFB | CD8T | 0.564 |
| PDCD1 | NUDT21 | CD8T | 0.564 |
| PDCD1 | MSC | CD8T | 0.563 |
| PDCD1 | MRPL28 | CD8T | 0.563 |
| PDCD1 | MPG | CD8T | 0.562 |
| PDCD1 | NDUFB3 | CD8T | 0.562 |
| PDCD1 | GBP5 | CD8T | 0.562 |
| PDCD1 | TAP1 | CD8T | 0.562 |
| PDCD1 | IL2RG | CD8T | 0.562 |
| PDCD1 | RAB11B | CD8T | 0.561 |
| PDCD1 | PSMB3 | CD8T | 0.561 |
| PDCD1 | PDCD6 | CD8T | 0.56 |
| PDCD1 | SUMO2 | CD8T | 0.559 |
| PDCD1 | PSMB9 | CD8T | 0.558 |
| PDCD1 | SIT1 | CD8T | 0.557 |
| PDCD1 | HCLS1 | CD8T | 0.556 |
| PDCD1 | SDF4 | CD8T | 0.555 |
| PDCD1 | YWHAB | CD8T | 0.554 |
| PDCD1 | AP2S1 | CD8T | 0.554 |
| PDCD1 | BST2 | CD8T | 0.554 |
| PDCD1 | AP1S1 | CD8T | 0.554 |
| PDCD1 | NUDT5 | CD8T | 0.553 |
| PDCD1 | VDAC1 | CD8T | 0.552 |
| PDCD1 | IAH1 | CD8T | 0.551 |
| PDCD1 | DBI | CD8T | 0.551 |
| PDCD1 | MTHFD2 | CD8T | 0.55 |
| PDCD1 | HNRNPLL | CD8T | 0.55 |
| PDCD1 | DUSP23 | CD8T | 0.549 |
| PDCD1 | MAD2L2 | CD8T | 0.548 |
| PDCD1 | LSM2 | CD8T | 0.548 |
| PDCD1 | CD79B | CD8T | 0.548 |
| PDCD1 | SRSF9 | CD8T | 0.547 |
| PDCD1 | ZYX | CD8T | 0.547 |
| PDCD1 | PRDM1 | CD8T | 0.547 |
| PDCD1 | LY6E | CD8T | 0.547 |
| PDCD1 | RBX1 | CD8T | 0.546 |
| PDCD1 | NAP1L4 | CD8T | 0.546 |
| PDCD1 | MDH2 | CD8T | 0.545 |
| PDCD1 | BIN1 | CD8T | 0.544 |
| PDCD1 | CHMP2A | CD8T | 0.544 |
| PDCD1 | PTPN7 | CD8T | 0.543 |
| PDCD1 | UBE2D2 | CD8T | 0.543 |
| PDCD1 | SRGN | CD8T | 0.543 |
| PDCD1 | FASLG | CD8T | 0.542 |
| PDCD1 | GGCT | CD8T | 0.542 |
| PDCD1 | TPI1 | CD8T | 0.541 |
| PDCD1 | CTSB | CD8T | 0.541 |
| PDCD1 | VAMP5 | CD8T | 0.541 |
| PDCD1 | ID2 | CD8T | 0.541 |
| PDCD1 | MIEN1 | CD8T | 0.54 |
| PDCD1 | NDUFA6 | CD8T | 0.54 |
| PDCD1 | ZBED2 | CD8T | 0.54 |
| PDCD1 | CFL1 | CD8T | 0.54 |
| PDCD1 | IL2RB | CD8T | 0.54 |
| PDCD1 | GSTO1 | CD8T | 0.54 |
| PDCD1 | LGALS3 | CD8T | 0.538 |
| PDCD1 | TMEM179B | CD8T | 0.537 |
| PDCD1 | POLR2G | CD8T | 0.537 |
| PDCD1 | HSD17B10 | CD8T | 0.536 |
| PDCD1 | NELFCD | CD8T | 0.536 |
| PDCD1 | MKI67 | CD8T | 0.536 |
| PDCD1 | NDUFS8 | CD8T | 0.535 |
| PDCD1 | MIIP | CD8T | 0.534 |
| PDCD1 | TYMP | CD8T | 0.534 |
| PDCD1 | SLC1A5 | CD8T | 0.534 |
| PDCD1 | NABP2 | CD8T | 0.533 |
| PDCD1 | CKS2 | CD8T | 0.533 |
| PDCD1 | FLII | CD8T | 0.533 |
| PDCD1 | SUMO1 | CD8T | 0.533 |
| PDCD1 | UBE2L6 | CD8T | 0.533 |
| PDCD1 | EZR | CD8T | 0.533 |
| PDCD1 | CLIC1 | CD8T | 0.532 |
| PDCD1 | PIN1 | CD8T | 0.532 |
| PDCD1 | TIMM8B | CD8T | 0.532 |
| PDCD1 | BSG | CD8T | 0.531 |
| PDCD1 | ADGRE5 | CD8T | 0.531 |
| PDCD1 | PRKAR1A | CD8T | 0.531 |
| PDCD1 | GTF3C6 | CD8T | 0.531 |
| PDCD1 | ARPC3 | CD8T | 0.531 |
| PDCD1 | B2M | CD8T | 0.53 |
| PDCD1 | ZNHIT1 | CD8T | 0.53 |
| PDCD1 | CALM3 | CD8T | 0.529 |
| PDCD1 | NDUFC1 | CD8T | 0.529 |
| PDCD1 | MCM5 | CD8T | 0.528 |
| PDCD1 | PSMA4 | CD8T | 0.528 |
| PDCD1 | YWHAQ | CD8T | 0.528 |
| PDCD1 | CCDC167 | CD8T | 0.528 |
| PDCD1 | UBE2N | CD8T | 0.527 |
| PDCD1 | RBCK1 | CD8T | 0.527 |
| PDCD1 | NDUFS6 | CD8T | 0.527 |
| PDCD1 | NCF4 | CD8T | 0.526 |
| PDCD1 | MYL6 | CD8T | 0.526 |
| PDCD1 | CALR | CD8T | 0.526 |
| PDCD1 | TPM4 | CD8T | 0.526 |
| PDCD1 | GPI | CD8T | 0.526 |
| PDCD1 | VASP | CD8T | 0.525 |
| PDCD1 | PSMB6 | CD8T | 0.525 |
| PDCD1 | AIP | CD8T | 0.524 |
| PDCD1 | PPP1R7 | CD8T | 0.524 |
| PDCD1 | TBCB | CD8T | 0.523 |
| PDCD1 | CD151 | CD8T | 0.523 |
| PDCD1 | SCAND1 | CD8T | 0.522 |
| PDCD1 | COMMD8 | CD8T | 0.522 |
| PDCD1 | LAMTOR1 | CD8T | 0.522 |
| PDCD1 | TMX1 | CD8T | 0.522 |
| PDCD1 | CCR5 | CD8T | 0.522 |
| PDCD1 | PRDX6 | CD8T | 0.521 |
| PDCD1 | FERMT3 | CD8T | 0.52 |
| PDCD1 | LPXN | CD8T | 0.52 |
| PDCD1 | REEP5 | CD8T | 0.519 |
| PDCD1 | PSMD4 | CD8T | 0.519 |
| PDCD1 | CLTB | CD8T | 0.519 |
| PDCD1 | SLTM | CD8T | 0.518 |
| PDCD1 | SNRPB2 | CD8T | 0.518 |

**Table S9 –** List of genes significantly correlated with PD1 from BRCA_EMTAB8107 database

| Gene | Gene 2 | Cell Type | Correlation value |
| --- | --- | --- | --- |
| PDCD1 | CXCL13 | CD8T | 0.869 |
| PDCD1 | TIGIT | CD8T | 0.821 |
| PDCD1 | CXCR6 | CD8T | 0.806 |
| PDCD1 | LAG3 | CD8T | 0.798 |
| PDCD1 | PTMS | CD8T | 0.796 |
| PDCD1 | IFNG | CD8T | 0.784 |
| PDCD1 | DUSP4 | CD8T | 0.775 |
| PDCD1 | MIR155HG | CD8T | 0.765 |
| PDCD1 | PHLDA1 | CD8T | 0.76 |
| PDCD1 | AC069363.1 | CD8T | 0.745 |
| PDCD1 | CSF1 | CD8T | 0.737 |
| PDCD1 | RGS1 | CD8T | 0.737 |
| PDCD1 | PLPP1 | CD8T | 0.723 |
| PDCD1 | SIRPG | CD8T | 0.721 |
| PDCD1 | CAMK1 | CD8T | 0.719 |
| PDCD1 | ACP5 | CD8T | 0.717 |
| PDCD1 | LINC01480 | CD8T | 0.704 |
| PDCD1 | MTHFD2 | CD8T | 0.704 |
| PDCD1 | ZBED2 | CD8T | 0.699 |
| PDCD1 | PTPN7 | CD8T | 0.696 |
| PDCD1 | PTTG1 | CD8T | 0.696 |
| PDCD1 | CD200 | CD8T | 0.693 |
| PDCD1 | TYMP | CD8T | 0.691 |
| PDCD1 | GAPDH | CD8T | 0.688 |
| PDCD1 | SUB1 | CD8T | 0.68 |
| PDCD1 | CCND2 | CD8T | 0.676 |
| PDCD1 | FABP5 | CD8T | 0.676 |
| PDCD1 | CD2 | CD8T | 0.663 |
| PDCD1 | ANXA5 | CD8T | 0.663 |
| PDCD1 | TNFRSF1B | CD8T | 0.659 |
| PDCD1 | AC133644.2 | CD8T | 0.659 |
| PDCD1 | IGFLR1 | CD8T | 0.654 |
| PDCD1 | TNFRSF9 | CD8T | 0.651 |
| PDCD1 | CLEC2D | CD8T | 0.649 |
| PDCD1 | PGAM1 | CD8T | 0.645 |
| PDCD1 | PDIA6 | CD8T | 0.642 |
| PDCD1 | ALOX5AP | CD8T | 0.641 |
| PDCD1 | FKBP1A | CD8T | 0.641 |
| PDCD1 | CD74 | CD8T | 0.638 |
| PDCD1 | KRT86 | CD8T | 0.637 |
| PDCD1 | LY6E | CD8T | 0.636 |
| PDCD1 | ITM2A | CD8T | 0.635 |
| PDCD1 | CTLA4 | CD8T | 0.633 |
| PDCD1 | TAP1 | CD8T | 0.632 |
| PDCD1 | CLTA | CD8T | 0.629 |
| PDCD1 | PKM | CD8T | 0.627 |
| PDCD1 | GEM | CD8T | 0.627 |
| PDCD1 | BST2 | CD8T | 0.622 |
| PDCD1 | HMOX1 | CD8T | 0.617 |
| PDCD1 | CCL3 | CD8T | 0.617 |
| PDCD1 | APOBEC3C | CD8T | 0.614 |
| PDCD1 | LAP3 | CD8T | 0.614 |
| PDCD1 | GNG5 | CD8T | 0.612 |
| PDCD1 | IFI16 | CD8T | 0.612 |
| PDCD1 | DYNLL1 | CD8T | 0.611 |
| PDCD1 | MYL6B | CD8T | 0.608 |
| PDCD1 | CD82 | CD8T | 0.607 |
| PDCD1 | OAS1 | CD8T | 0.606 |
| PDCD1 | IFI27L2 | CD8T | 0.606 |
| PDCD1 | IFI6 | CD8T | 0.602 |
| PDCD1 | SH2D2A | CD8T | 0.602 |
| PDCD1 | BTG3 | CD8T | 0.601 |
| PDCD1 | TPI1 | CD8T | 0.6 |
| PDCD1 | CAPZB | CD8T | 0.599 |
| PDCD1 | BATF | CD8T | 0.596 |
| PDCD1 | SRGN | CD8T | 0.595 |
| PDCD1 | CCDC50 | CD8T | 0.594 |
| PDCD1 | ISG15 | CD8T | 0.591 |
| PDCD1 | MT1E | CD8T | 0.591 |
| PDCD1 | ARPC2 | CD8T | 0.589 |
| PDCD1 | PARK7 | CD8T | 0.589 |
| PDCD1 | PSMB9 | CD8T | 0.585 |
| PDCD1 | ITGB1 | CD8T | 0.584 |
| PDCD1 | MT2A | CD8T | 0.584 |
| PDCD1 | ID2 | CD8T | 0.584 |
| PDCD1 | COX5A | CD8T | 0.583 |
| PDCD1 | CTSC | CD8T | 0.583 |
| PDCD1 | GBP5 | CD8T | 0.581 |
| PDCD1 | PSMB8 | CD8T | 0.58 |
| PDCD1 | ARPC1B | CD8T | 0.579 |
| PDCD1 | CORO1B | CD8T | 0.578 |
| PDCD1 | RHOA | CD8T | 0.575 |
| PDCD1 | PPM1M | CD8T | 0.569 |
| PDCD1 | CKS2 | CD8T | 0.569 |
| PDCD1 | MX1 | CD8T | 0.569 |
| PDCD1 | RANBP1 | CD8T | 0.564 |
| PDCD1 | RHOB | CD8T | 0.562 |
| PDCD1 | IFI44 | CD8T | 0.56 |
| PDCD1 | FAM166B | CD8T | 0.56 |
| PDCD1 | POMP | CD8T | 0.559 |
| PDCD1 | CD2BP2 | CD8T | 0.558 |
| PDCD1 | VAMP5 | CD8T | 0.558 |
| PDCD1 | PHPT1 | CD8T | 0.557 |
| PDCD1 | PLSCR1 | CD8T | 0.557 |
| PDCD1 | TXNDC17 | CD8T | 0.555 |
| PDCD1 | MAP1LC3A | CD8T | 0.554 |
| PDCD1 | IFI35 | CD8T | 0.553 |
| PDCD1 | IRF7 | CD8T | 0.553 |
| PDCD1 | UBE2L6 | CD8T | 0.552 |
| PDCD1 | CALM3 | CD8T | 0.549 |
| PDCD1 | NBL1 | CD8T | 0.548 |
| PDCD1 | OASL | CD8T | 0.546 |
| PDCD1 | LCP2 | CD8T | 0.544 |
| PDCD1 | JAML | CD8T | 0.544 |
| PDCD1 | YWHAH | CD8T | 0.541 |
| PDCD1 | ZFP36L1 | CD8T | 0.539 |
| PDCD1 | SH3BGRL3 | CD8T | 0.539 |
| PDCD1 | RBCK1 | CD8T | 0.538 |
| PDCD1 | SUMO2 | CD8T | 0.538 |
| PDCD1 | HSBP1 | CD8T | 0.538 |
| PDCD1 | FASLG | CD8T | 0.538 |
| PDCD1 | TMEM14A | CD8T | 0.537 |
| PDCD1 | SHISA5 | CD8T | 0.537 |
| PDCD1 | PDLIM7 | CD8T | 0.536 |
| PDCD1 | NDUFV2 | CD8T | 0.535 |
| PDCD1 | COTL1 | CD8T | 0.534 |
| PDCD1 | NCF4 | CD8T | 0.534 |
| PDCD1 | SAMD9L | CD8T | 0.534 |
| PDCD1 | ICOS | CD8T | 0.534 |
| PDCD1 | CDKN2A | CD8T | 0.533 |
| PDCD1 | CARD16 | CD8T | 0.533 |
| PDCD1 | SDC4 | CD8T | 0.53 |
| PDCD1 | CSF2 | CD8T | 0.53 |
| PDCD1 | VDAC1 | CD8T | 0.528 |
| PDCD1 | SURF4 | CD8T | 0.527 |
| PDCD1 | CD3D | CD8T | 0.527 |
| PDCD1 | SERF2 | CD8T | 0.526 |
| PDCD1 | COMMD7 | CD8T | 0.525 |
| PDCD1 | TPM4 | CD8T | 0.525 |
| PDCD1 | C4orf48 | CD8T | 0.523 |
| PDCD1 | TNFRSF18 | CD8T | 0.522 |
| PDCD1 | CCR1 | CD8T | 0.522 |
| PDCD1 | LBH | CD8T | 0.522 |
| PDCD1 | RBX1 | CD8T | 0.521 |
| PDCD1 | DEK | CD8T | 0.521 |
| PDCD1 | NPDC1 | CD8T | 0.52 |
| PDCD1 | DRAP1 | CD8T | 0.52 |
| PDCD1 | KLHDC7B | CD8T | 0.519 |
| PDCD1 | B2M | CD8T | 0.518 |
| PDCD1 | MZB1 | CD8T | 0.518 |
| PDCD1 | WDR1 | CD8T | 0.518 |
| PDCD1 | C3orf14 | CD8T | 0.517 |
| PDCD1 | SCAMP2 | CD8T | 0.517 |
| PDCD1 | PPP1CC | CD8T | 0.516 |
| PDCD1 | SNRPG | CD8T | 0.516 |
| PDCD1 | PLEKHF1 | CD8T | 0.514 |

**Table S10 –** List of genes significantly correlated with PD1 from BRCA_GSE161529 database

| Gene | Gene 2 | Cell Type | Correlation value |
| --- | --- | --- | --- |
| PDCD1 | TIGIT | CD8T | 0.758 |
| PDCD1 | CD3D | CD8T | 0.756 |
| PDCD1 | CD2 | CD8T | 0.749 |
| PDCD1 | TRBC1 | CD8T | 0.741 |
| PDCD1 | TRAC | CD8T | 0.721 |
| PDCD1 | LCK | CD8T | 0.712 |
| PDCD1 | CTLA4 | CD8T | 0.698 |
| PDCD1 | IFNG | CD8T | 0.696 |
| PDCD1 | TRBC2 | CD8T | 0.693 |
| PDCD1 | CD3G | CD8T | 0.683 |
| PDCD1 | ZBED2 | CD8T | 0.682 |
| PDCD1 | CD247 | CD8T | 0.679 |
| PDCD1 | GPR171 | CD8T | 0.665 |
| PDCD1 | LAIR2 | CD8T | 0.65 |
| PDCD1 | CXCR6 | CD8T | 0.648 |
| PDCD1 | CD7 | CD8T | 0.644 |
| PDCD1 | SPOCK2 | CD8T | 0.636 |
| PDCD1 | CD3E | CD8T | 0.636 |
| PDCD1 | SH2D2A | CD8T | 0.635 |
| PDCD1 | IL2RB | CD8T | 0.623 |
| PDCD1 | ICOS | CD8T | 0.621 |
| PDCD1 | PTPN7 | CD8T | 0.62 |
| PDCD1 | CLEC2D | CD8T | 0.609 |
| PDCD1 | APOBEC3G | CD8T | 0.607 |
| PDCD1 | SIT1 | CD8T | 0.607 |
| PDCD1 | KLRB1 | CD8T | 0.6 |
| PDCD1 | TBC1D10C | CD8T | 0.591 |
| PDCD1 | LINC01943 | CD8T | 0.568 |
| PDCD1 | GZMM | CD8T | 0.566 |
| PDCD1 | S1PR4 | CD8T | 0.564 |
| PDCD1 | CD8A | CD8T | 0.564 |
| PDCD1 | TNFRSF9 | CD8T | 0.557 |
| PDCD1 | CD27 | CD8T | 0.555 |
| PDCD1 | CD8B | CD8T | 0.553 |
| PDCD1 | CXCR3 | CD8T | 0.552 |
| PDCD1 | GZMA | CD8T | 0.549 |
| PDCD1 | IL2RG | CD8T | 0.549 |
| PDCD1 | RAC2 | CD8T | 0.543 |
| PDCD1 | CTSW | CD8T | 0.537 |
| PDCD1 | CORO1A | CD8T | 0.536 |
| PDCD1 | LAT | CD8T | 0.523 |
